# Context‐Dependent Inverse Association of Glucose–Lipid Metabolic Status With Anemia: Evidence From Multinational Multicohort Analyses

**DOI:** 10.1002/advs.78037

**Published:** 2026-09-29

**Authors:** Guosong Jiang, Xinxing Wang, Lianfeng Xie, Wenna Ding, Yingyong Yin, Mei Yao, Zhibo Cui, Kaiqiang Tang, Lijie Zhong, Zhi Wang, Jia Wang, Lei Lei, Xiaoxiao Qu, Chi‐E. Qiu

**Affiliations:** ^1^ Department of Pulmonary and Critical Care Medicine Northeast Yunnan Central Hospital Zhaotong Yunnan China; ^2^ Kunming Medical University Kunming Yunnan China; ^3^ Department of Clinical Laboratory Chengdu BOE Hospital Chengdu Sichuan China; ^4^ Department of Ophthalmology First Affiliated Hospital of Gannan Medical University Ganzhou Jiangxi China; ^5^ Department of Clinical Nutrition Northeast Yunnan Central Hospital Zhaotong Yunnan China; ^6^ Information Technology Department Northeast Yunnan Central Hospital Zhaotong Yunnan China; ^7^ Department of Radiology Chongqing General Hospital Chongqing University Chongqing China; ^8^ Postgraduates At the First Clinical Medicine of Gannan Medical University Ganzhou Jiangxi China; ^9^ The Affiliated Ganzhou Hospital of Nanchang University Ganzhou Jiangxi China; ^10^ Department of Critical Care Medicine The Second People's Hospital of Zhaotong Zhaotong Yunnan China; ^11^ Hematology Department Northeast Yunnan Central Hospital Zhaotong Yunnan China; ^12^ Department of Clinical Laboratory The Second Affiliated Hospital & Yuying Children's Hospital of Wenzhou Medical University Wenzhou Zhejiang China

**Keywords:** anemia, cardiovascular disease, insulin resistance, multi‐national cohort, systemic inflammation

## Abstract

**Background:**

The hematological implications of systemic glucose–lipid metabolic status remain poorly understood. We investigated its association with anemia across diverse populations.

**Methods:**

Six analytic cohorts comprising 70,350 unique participants from China, the United States, and the United Kingdom were analyzed using cross‐sectional, longitudinal, and extended time‐to‐event approaches. Dose–response patterns, anemia morphology, effect modification, and an exploratory renal‐function‐related pathway were evaluated.

**Results:**

A higher glucose–lipid metabolic index was consistently associated with lower odds of prevalent and incident anemia, with fully adjusted ORs ranging from 0.53 to 0.87 per 1‐unit increase. Extended ELSA follow‐up showed a similar association (HR, 0.61; 95% CI, 0.43–0.87). Longitudinal associations were approximately linear, whereas cross‐sectional relationships flattened at higher levels. The inverse association was evident for microcytic and normocytic anemia, attenuated with greater systemic inflammation, and stronger among participants with heart disease. An exploratory indirect association through cystatin C accounted for 16.33% of the observed association.

**Conclusions:**

Higher glucose–lipid metabolic status was reproducibly associated with lower anemia occurrence, but this relationship varied across inflammatory, cardiovascular, and renal contexts. These findings suggest that metabolic states conventionally linked to vascular risk may carry distinct implications for hematological homeostasis and metabolic reserve.

## Graphical abstract

Across six multinational cohorts, higher triglyceride–glucose index values are consistently associated with lower prevalent and incident anemia, despite their established adverse cardiometabolic significance. This inverse relationship varies across inflammatory, cardiovascular, and renal contexts, suggesting that glucose–lipid metabolic status carries distinct, context‐dependent implications for hematological homeostasis and vascular risk across populations.

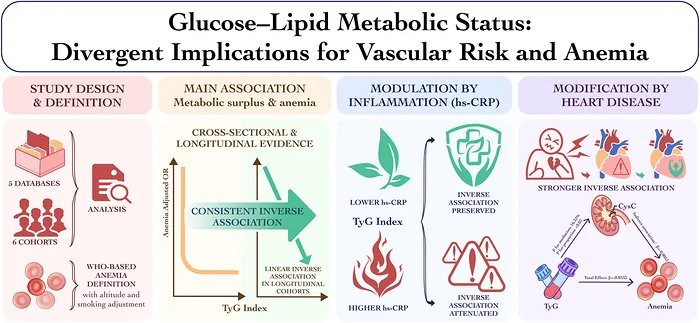

**Abbreviations**
AP: Attributable Proportion
BMI: Body Mass Index
CHARLS: China Health and Retirement Longitudinal Study
CI: Confidence Interval
CKD: Chronic Kidney Disease
DAG: Directed Acyclic Graph
ELSA: English Longitudinal Study of Ageing
EPO: Erythropoietin
Hb: Hemoglobin
hs‐CRP: high‐sensitivity C‐reactive protein
IGF‐1: Insulin‐Like Growth Factor 1
IQR: Interquartile Range
MCV: Mean Corpuscular Volume
METS‐IR: Metabolic Score for Insulin Resistance
MICE: Multiple Imputation by Chained Equations
NCHS: National Center for Health Statistics
NHANES: National Health and Nutrition Examination Survey
OR: Odds Ratio
RCS: Restricted Cubic Splines
RERI: Relative Excess Risk due to Interaction
SD: Standard Deviation
TyG: Triglyceride‐glucose index
VIF: Variance Inflation Factors
WBC: White Blood Cell
WHO: World Health Organization

## 1 Introduction

Anemia is a common manifestation of aging and chronic disease and is associated with substantial functional impairment and adverse clinical outcomes [1, 2]. Its pathogenesis is heterogeneous, with nutritional deficiency, inflammation, and renal dysfunction representing well‐established contributors [1, 3, 4, 5]. Yet erythropoiesis is itself a metabolically demanding process that requires continuous energy, substrate availability, and growth‐factor signaling [6]. Compared with these conventional determinants, the role of systemic metabolic status in maintaining hematological homeostasis has received considerably less attention. This question may be particularly relevant in older adults and patients with cardiorenal disease, in whom metabolic dysfunction, inflammation, renal impairment, and anemia frequently coexist [5, 7].

The triglyceride–glucose (TyG) index provides an intriguing framework for examining this relationship. As a surrogate of insulin resistance, higher TyG has been associated predominantly with adverse cardiovascular, metabolic, and renal outcomes and is therefore conventionally regarded as a metabolic risk marker [8, 9]. However, insulin resistance develops within a broader glucose–lipid metabolic milieu and, particularly during compensatory stages, is often accompanied by hyperinsulinemia [10]. Although TyG does not directly measure circulating insulin or anabolic signaling, insulin and insulin‐like growth factor‐1 signaling have established erythropoiesis‐supporting effects, including promotion of erythroid progenitor proliferation and survival [11, 12, 13]. Thus, the metabolic phenotype captured by TyG may have different implications for hematopoietic homeostasis than for vascular health, raising a paradoxical but clinically relevant question: can a metabolic state associated with vascular risk simultaneously carry information related to hematological reserve?

Emerging epidemiological evidence supports this possibility but remains limited. Obesity and related metabolic phenotypes have been associated with hemoglobin levels [14, 15], and a recent CHARLS analysis reported inverse associations of several insulin‐resistance surrogates, including TyG, with anemia [16]. However, whether this relationship precedes anemia development, reproduces across populations and settings, and varies according to anemia phenotype or inflammatory, cardiovascular, and renal context remains unclear. These gaps leave the proposed concept of a context‐dependent metabolic reserve relevant to erythropoiesis largely untested.

To address these questions, we assembled six analytic cohorts comprising 70,350 unique participants from China, the United States, and the United Kingdom, integrating longitudinal population studies, nationally representative data, and contemporary real‐world clinical cohorts. We evaluated the reproducibility and dose–response characteristics of the TyG–anemia association and examined its variation across anemia phenotypes and inflammatory, cardiovascular, and renal contexts. Given its greater sensitivity to kidney‐function‐related variation and lower dependence on muscle mass than serum creatinine, cystatin C was additionally examined as a renal‐function marker in an exploratory pathway analysis [17, 18]. Alternative insulin‐resistance surrogates and extensive sensitivity analyses were used to assess the robustness and specificity of the findings. Through this multidimensional approach, we sought to determine whether the inverse metabolic–hematological relationship represents a reproducible epidemiological phenomenon and to define the clinical contexts in which it is most evident.

## 2 Methods

### 2.1 Data Source

To ensure robust cross‐population validation, this study integrated data from five databases, constructed six analytic cohorts across China, the United States, and the United Kingdom, and categorized them into population‐based surveys and real‐world hospital‐based registries.

For general population analysis, we leveraged three nationally representative datasets. The China Health and Retirement Longitudinal Study (CHARLS), initiated in 2011, served as the primary prospective cohort and employed multistage probability sampling to represent the Chinese population across 28 provinces. To provide cross‐national perspectives, we incorporated harmonized data from the English Longitudinal Study of Ageing (ELSA), a multidisciplinary study of the English population aged 50 years and older, and the National Health and Nutrition Examination Survey (NHANES), a continuous program conducted by the U.S. National Center for Health Statistics (NCHS), utilizing a complex, stratified probability design [19, 20, 21].

To validate the findings in contemporary clinical settings (2022–2025), we extracted real‐world data from the health checkup centers of two tertiary hospitals in China. The Zhaotong Cohort, which represents a unique high‐altitude population in Southwest China, was obtained from the Northeast Yunnan Central Hospital (Zhaotong, Yunnan Province). The Ganzhou Cohort, representing a large population in southern China, was obtained from the First Affiliated Hospital of Gannan Medical University (Ganzhou, Jiangxi Province).

Ethical approval was obtained from the respective institutional review boards: Peking University for CHARLS (IRB00001052‐11015), the NCHS Research Ethics Review Board for NHANES, and the London Multicentre Research Ethics Committee for ELSA, with all participants providing written informed consent. The protocols for the hospital‐based cohorts were approved by the Ethics Committee of the Northeast Yunnan Central Hospital (No. XSD2026023) and the First Affiliated Hospital of Gannan Medical University (No. LLSL‐2026147). Given the retrospective nature of the clinical analysis, which utilized strictly de‐identified, pre‐existing electronic medical records, with no direct patient contact and re‐identification impossible, the requirement for informed consent was waived by the ethics committee in accordance with the Declaration of Helsinki.

### 2.2 Study Population

This multicohort observational study included six analytical cohorts derived from five independent data sources in China, the United States, and the United Kingdom: the China Health and Retirement Longitudinal Study (CHARLS), the English Longitudinal Study of Ageing (ELSA), the National Health and Nutrition Examination Survey (NHANES), and two contemporary clinical cohorts from Ganzhou and Zhaotong, China. The study integrated longitudinal data from CHARLS (Waves 1–3, 2011–2015) and ELSA (Waves 4–6, 2008–2012), repeated cross‐sectional NHANES data (1999–2020), and clinical data collected during 2022–2025. A harmonized selection framework was applied across cohorts (Figure 1). Participants aged < 45 years, pregnant women, and individuals lacking data required to calculate the TyG index or ascertain hemoglobin status were excluded. In the Ganzhou cohort, a small number of clearly abnormal anthropometric records, including BMI values of approximately 9–10 kg/m^2^ that were highly suggestive of measurement or data‐entry errors, were excluded during data quality control.

**FIGURE 1:**
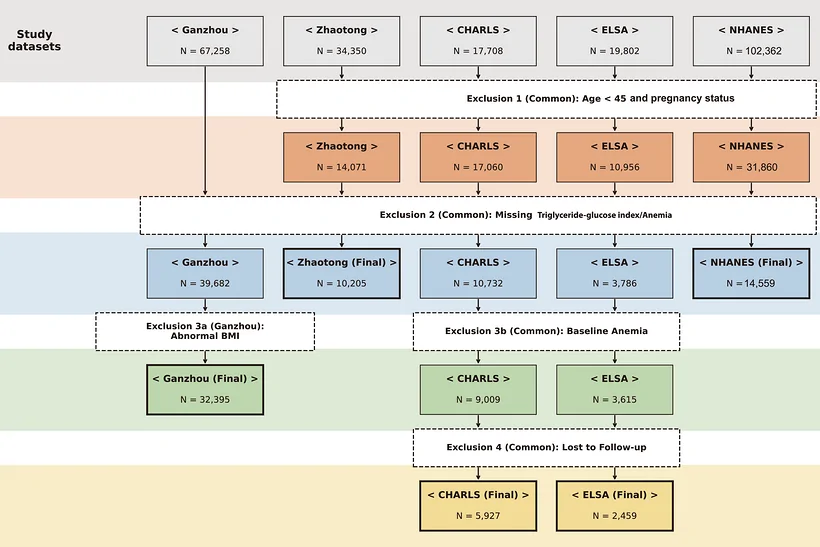
Flowchart of study design and participant selection. The flowchart illustrates the derivation of the final analytical cohorts from five independent datasets: Ganzhou, Zhaotong, CHARLS, ELSA, and NHANES. Eligibility criteria common to the analytic cohorts included age ≥ 45 years and non‐pregnant status. These criteria were applied during cohort selection in Zhaotong, CHARLS, ELSA, and NHANES, whereas the Ganzhou clinical registry source population had already been restricted accordingly before the present analytic workflow. Participants with missing data for the TyG index or baseline anemia status were subsequently excluded as applicable. An additional quality‐control step excluding clearly abnormal BMI records was applied to the Ganzhou clinical registry. For the primary longitudinal analyses, participants with prevalent anemia at baseline or without a valid follow‐up hemoglobin measurement were excluded, yielding final longitudinal samples of *N* = 5,927 for CHARLS and *N* = 2,459 for ELSA. The final cross‐sectional analytical samples comprised Ganzhou (*N* = 32,395), Zhaotong (*N* = 10,205), NHANES (*N* = 14,559), and the baseline CHARLS cohort (*N* = 10,732). CHARLS, China Health and Retirement Longitudinal Study; ELSA, English Longitudinal Study of Ageing; NHANES, National Health and Nutrition Examination Survey; BMI, Body Mass Index.

For longitudinal analyses of incident anemia, participants with anemia at baseline or without valid follow‐up hemoglobin measurements were excluded, yielding 5,927 participants in CHARLS and 2,459 in ELSA. The primary longitudinal analyses used harmonized 4‐year follow‐up windows (CHARLS Waves 1–3 and ELSA Waves 4–6). ELSA follow‐up was additionally extended through Wave 8 for time‐to‐event analyses, with participants retained until the first detected incident anemia or their last anemia‐free hemoglobin assessment. Cross‐sectional analyses included CHARLS (*N* = 10,732), NHANES (*N* = 14,559), Ganzhou (*N* = 32,395), and Zhaotong (*N* = 10,205). Overall, 70,350 unique participants contributed to the study.

### 2.3 Exposure

The primary exposure was glucose–lipid metabolic status, operationalized using the TyG index, a surrogate marker of insulin resistance. The TyG index was calculated as [22]:

$$\begin{array}{c} \mathrm{TyG}\;\mathrm{index}=\ln\left[\frac{\begin{array}{c} \mathrm{Fasting}\;\mathrm{Triglyceride}\;\left(\mathrm{mg}/\mathrm{dL}\right)\\ \;\times \;\mathrm{Fasting}\;\mathrm{Glucose}\;(\mathrm{mg}/\mathrm{dL}) \end{array}}{2}\right]\; \end{array}$$

For longitudinal analyses, TyG was calculated from baseline fasting blood measurements before subsequent anemia ascertainment; for cross‐sectional analyses, TyG and hemoglobin were assessed at the same examination. Glucose and triglyceride concentrations reported in mmol/L in CHARLS, ELSA, Zhaotong, and Ganzhou were converted to mg/dL using conversion factors of 18.02 and 88.57, respectively.

### 2.4 Outcome Assessment: Definition and Adjustment of Anemia

The primary outcome was anemia, defined according to World Health Organization (WHO) criteria. Hemoglobin (Hb) concentrations were measured using automated hematology analyzers across all cohorts. Under standard conditions, anemia was defined as an Hb concentration < 13.0 g/dL (130 g/L) in men and < 12.0 g/dL (120 g/L) in non‐pregnant women [23, 24].

To account for the physiological increase in Hb associated with residence at higher altitude and to reduce potential misclassification of anemia, altitude‐specific corrections were applied to the Zhaotong cohort (mean altitude approximately 2,000 m) and to participants from relevant CHARLS locations. The altitude‐specific adjustment value (ΔHb) was calculated using the WHO‐recommended formula [23, 25]:

$\Delta \mathrm{Hb}\;\left(\frac{g}{L}\right)=0.0056384\times A+0.0000003\times A^{2}$ [23, 25], where A denotes altitude in meters. The calculated adjustment value (g/L) was converted to g/dL and applied to the measured Hb concentration to obtain the corresponding sea‐level‐equivalent value before anemia classification.

Because cigarette smoking may increase measured Hb concentrations and consequently affect anemia classification, smoking‐specific adjustments were also incorporated into the diagnostic criteria. For current smokers, the Hb threshold for defining anemia was increased by 0.3 g/dL; for heavy smokers (>20 cigarettes/day), where quantitative smoking information was available, the threshold was increased by 0.6 g/dL [23]. Thus, anemia status was determined after incorporating sex‐specific WHO thresholds together with applicable altitude‐ and smoking‐related adjustments. In the longitudinal CHARLS and ELSA analyses, incident anemia was defined as newly identified anemia at follow‐up according to these criteria among participants who were free of anemia at baseline.

### 2.5 Covariates and Exploratory Pathway Marker

Covariate selection was informed by a directed acyclic graph (DAG) constructed from established biological and epidemiological evidence (Supplementary Figure S1). Potential confounders were grouped into sociodemographic, lifestyle/anthropometric, and clinical domains. Sociodemographic factors included age, sex, ethnicity, education, marital status, and residence; lifestyle and anthropometric factors included BMI, smoking, and alcohol consumption. Clinical covariates included hypertension, diabetes mellitus, dyslipidemia, cardiovascular disease, chronic kidney disease (CKD), liver disease, cancer, arthritis, and digestive disorders, together with cohort‐specific laboratory measures, nutritional indicators, and medication use where available. Detailed definitions and measurement protocols are provided in the Supplementary Materials.

Cystatin C was evaluated as a renal‐function‐related intermediate marker and was therefore not included as a confounder in the primary adjustment models. Its potential role in the association between the TyG index and anemia was evaluated separately in exploratory pathway analyses rather than interpreted as direct evidence of a specific renal hemodynamic mechanism.

### 2.6 Statistical Analysis

All analyses were performed using R Statistical Software (version 4.2.2) and the Free Statistics Analysis platform (version 2.4.0). Continuous variables are presented as mean ± standard deviation or median (interquartile range), and categorical variables as numbers and percentages. Between‐group comparisons used analysis of variance, the Kruskal–Wallis test, or the chi‐square test, as appropriate. Missing covariate data were handled using multiple imputation by chained equations (MICE), with complete‐case analyses performed as a sensitivity analysis.

For NHANES, all analyses accounted for the complex multistage probability sampling design by incorporating survey weights, strata, and primary sampling units. Because the TyG index required fasting glucose and triglyceride measurements, fasting‐subsample weights were used. The corresponding cycle‐specific weights were harmonized across the pooled 1999–2020 data: WTSAF4YR for 1999–2002, WTSAF2YR for 2003–2016, and WTSAFPRP for the 2017–March 2020 pre‐pandemic cycle. Survey design variables were incorporated throughout the descriptive, regression, and sensitivity analyses to obtain nationally representative estimates and appropriate variance estimates.

Associations between the TyG index and anemia were evaluated using cohort‐specific multivariable logistic regression. In CHARLS and ELSA, the primary longitudinal analyses estimated incident anemia over harmonized 4‐year follow‐up intervals; cross‐sectional analyses assessed prevalent anemia in CHARLS, NHANES, Ganzhou, and Zhaotong. Four sequential models were fitted: Model 1 was unadjusted; Model 2 incorporated sociodemographic and lifestyle factors; Model 3 additionally included clinical comorbidities; and Model 4 further incorporated available cohort‐specific laboratory measures and medications. TyG was examined both continuously and in cohort‐specific quartiles. Multicollinearity and influential observations were assessed using variance inflation factors and Cook's distance, respectively (Supplementary Figures S2–S7). Restricted cubic splines (RCSs) with knots at the fifth, 35th, 65th, and 95th percentiles were used to characterize dose–response patterns; inflection points were estimated when evidence of nonlinearity was present.

Stratified analyses were conducted according to demographic, inflammatory, and clinical characteristics. Heterogeneity in the continuous TyG association across strata was assessed using TyG‐by‐subgroup terms and likelihood‐ratio tests. Separately, selected modifiers, including systemic inflammation and heart disease, were examined using a joint‐exposure framework in which TyG was dichotomized at the cohort‐specific median. Multiplicative interaction was evaluated using the product term between dichotomized TyG and the corresponding binary modifier, whereas additive interaction was quantified using the relative excess risk due to interaction (RERI), attributable proportion due to interaction (AP), and synergy index (SI). Candidate intermediate markers from inflammatory and renal‐function‐related domains were examined in exploratory pathway analyses. Cystatin C was subsequently evaluated as a renal‐function‐related intermediate marker using bootstrap mediation analysis with 1,000 resamples. Because TyG and cystatin C were measured concurrently at baseline, these mediation estimates were interpreted as exploratory rather than causal.

To evaluate the primary longitudinal findings using time‐to‐event methods, ELSA follow‐up was extended from Wave 4 through Wave 8. Because incident anemia was ascertained at discrete study waves, an interval‐censored time‐to‐event approach was used to estimate HRs for incident anemia using the same sequential adjustment framework. Restricted cubic spline analyses were used to characterize the dose–response relationship, and proportional‐hazards assumptions were assessed. A complete‐follow‐up analysis was additionally performed as a sensitivity analysis.

Additional sensitivity analyses examined anemia morphology, exclusion of selected high‐risk subpopulations, extreme‐value handling, alternative TyG categorization, METS‐IR, and E‐values for unmeasured confounding. Where data were available, models were further adjusted for sleep, physical activity, and frailty, as well as dietary and nutritional factors, serum albumin, and depressive symptoms [26, 27, 28, 29], together with cardiometabolic medication use. Exploratory mediation analyses were additionally stratified by baseline CKD status. All tests were two‐sided, with *P* < 0.05 considered statistically significant.

## 3 Results

### 3.1 Baseline Characteristics of the Study Population

Across the six analytic cohorts, 70,350 unique participants contributed data to the study (Table 1). Baseline demographic and clinical characteristics varied across populations. Participants in the contemporary hospital‐based Zhaotong and Ganzhou cohorts (2022–2025) had a mean age of approximately 55 years, compared with approximately 59–63 years in the population‐based CHARLS, ELSA, and NHANES cohorts. Anemia prevalence also differed across cohorts, ranging from 17.7% at baseline in the CHARLS longitudinal cohort to 8.1%–8.3% in the contemporary Zhaotong and Ganzhou cohorts.

**TABLE 1:** Baseline characteristics of participants across six multi‐national cohorts (Data spanning 1999–2025).

Variables	CHARLS‐Longitudinal	ELSA	CHARLS‐Cross‐sectional	NHANES	Zhaotong	Ganzhou
Total *N* = 70,350*	*N* = 5,927	*N* = 2,459	*N* = 10,732	*N* = 14,559	*N* = 10,205	*N* = 32,395
Universal Core Markers						
Age, y	58.8 ± 8.5	63.1 ± 7.0	59.5 ± 9.4	60.19 (0.14)	54.9 ± 7.7	55.3 ± 8.0
Sex, n (%)						
Male	2,732 (46.1)	1,367 (55.6)	4,968 (46.3)	7,187 (47.1)	6,352 (62.2)	17,947 (55.4)
Female	3,195 (53.9)	1,092 (44.4)	5,764 (53.7)	7,372 (53.0)	3,853 (37.8)	14,448 (44.6)
Marital status, n (%)						NA
No	634 (10.7)	707 (28.7)	1,299 (12.1)	5,471 (31.6)	1,054 (12.6)	
Yes	5,293 (89.3)	1,752 (71.3)	9,433 (87.9)	8,971 (68.4)	7,344 (87.4)	
BMI, kg/m2	23.5 (21.2, 26.1)	27.0 (24.7, 30.4)	23.1 (20.8, 25.8)	28.10 (24.66, 32.36)	24.6 (22.5, 26.8)	24.1(22.1, 26.1)
BUN, mmol/L	5.6 ± 1.5	NA	5.6 ± 1.7	5.00 (3.93,6.07)	5.6 ± 1.6	5.0 ± 1.4
Creatinine, µmol/L	68.1 ± 15.4	NA	69.3 ± 21.2	76.02 (63.65, 88.40)	72.3 ± 24.2	75.2 ± 22.5
WBC, ×10^9^ /L	6.3 ± 1.9	6.1 ± 1.9	6.2 ± 2.1	6.40 (5.40,7.70)	5.9 ± 1.6	6.0 ± 1.7
PLT, ×10^9^ /L	212.8 ± 74.4	NA	211.7 ± 74.9	246.08 (0.98)	232.3 ± 58.5	246 ± 61.3
MCV, fL	91.1 ± 7.5	NA	90.6 ± 8.6	90.64 (0.09)	92.6 ± 6.3	88.7 ± 7.6
TyG	8.7 ± 0.7	8.6 ± 0.5	8.7 ± 0.7	8.8 ± 0.7	8.9 ± 0.7	8.7 ± 0.6
Hs‐CRP, mg/L	1.0 (0.6, 2.1)	1.7 (0.8, 3.5)	1.0 (0.6, 2.2)	NA	NA	NA
Glycated hemoglobin, %	5.3 ± 0.8	5.8 ± 0.5	5.3 ± 0.8	NA	NA	NA
Anemia, n (%)						
No	4,879 (82.3)	2,233 (90.8)	9,009 (83.9)	12,843 (91.99)	9,358 (91.7)	29,765 (91.9)
Yes	1,048 (17.7)	226 (9.2)	1,723 (16.1)	1,716 (8.01)	847 (8.3)	2,630 (8.1)
Lifestyle						
Smoking status, n (%)						NA
Never	3,651 (61.6)	1,093 (44.4)	6,605 (61.5)	7,143 (49.0)	7,639 (76.4)	
Ever	2,276 (38.5)	1,366 (55.6)	4,127 (38.5)	7,416 (51.0)	2,359 (23.6)	
Drinking status, n (%)						NA
Never	4,349 (73.4)	169 (7.3)	7,891 (73.6)	1,985 (12.2)	8,505 (85.2)	
Ever	1,574 (26.5)	2,136 (92.7)	2,835 (26.4)	10,983 (87.8)	1,480 (14.8)	
Health Status						
Hypertension, n (%)						NA
No	3,084 (52.2)	1,667 (67.8)	5,696 (53.3)	5,845 (47.2)	7,836 (76.8)	
Yes	2,826 (47.8)	792 (32.2)	4,999 (46.7)	8,711 (53.8)	2,369 (23.2)	
DM, n (%)						NA
No	4,971 (84.7)	2,397 (97.5)	8,977 (84.4)	10,339 (77.8)	9,418 (92.3)	
Yes	901 (15.3)	62 (2.5)	1,658 (15.6)	4,220 (22.2)	787 (7.7)	
Cancer, n (%)					NA	NA
No	5,842 (99.2)	2,308 (93.9)	10,574 (99.1)	12,449 (84.9)		
Yes	50 (0.8)	151 (6.1)	99 (0.9)	2,093 (15.1)		
CPD, n (%)				NA	NA	NA
No	5,333 (90.4)	2,382 (96.9)	9,620 (90.0)			
Yes	569 (9.6)	77 (3.1)	1,064 (10.0)			
Heart disease, n (%)					NA	NA
No	5,119 (87.0)	2,181 (88.8)	9,333 (87.5)	12834 (89.9)		
Yes	766 (13.0)	276 (11.2)	1,335 (12.5)	1717 (10.1)		
Stroke, n (%)					NA	NA
No	5,762 (97.5)	2,416 (98.3)	10,405 (97.3)	13,586 (93.5)		
Yes	145 (2.5)	43 (1.7)	292 (2.7)	952 (6.5)		
Depression, n (%)		NA			NA	NA
No	5,834 (98.8)		10,535 (98.6)	7,599 (77.6)		
Yes	68 (1.2)		148 (1.4)	2,503 (22.4)		
Arthritis, n (%)					NA	NA
No	3,884 (65.7)	1,696 (69.0)	6,970 (65.1)	8,491 (59.5)		
Yes	2,030 (34.3)	7,63 (31.0)	3,733 (34.9)	6,033 (40.4)		
Dyslipidemia, n (%)		NA			NA	NA
No	5,125 (88.3)		9,414 (89.6)	2,653 (17.2)		
Yes	676 (11.7)		1,095 (10.4)	11,906 (82.8)		
Liver Diseases, n (%)		NA		NA	NA	NA
No	5,667 (96.4)		10,263 (96.4)			
Yes	214 (3.6)		385 (3.6)			
CKD, n (%)		NA			NA	NA
No	5,542 (94.3)		10,006 (93.9)	10,493 (79.3)		
Yes	338 (5.7)		652 (6.1)	3,873 (20.7)		
Chronic gastritis, n (%)		NA		NA	NA	NA
No	4,573 (77.5)		8,275 (77.4)			
Yes	1,330 (22.5)		2,420 (22.6)			
Asthma, n (%)		NA			NA	NA
No	5,617 (95.1)		10,197 (95.4)	12,690 (86.8)		
Yes	290 (4.9)		491 (4.6)	1,848 (13.2)		

Data are presented as mean ± standard deviation (SD), median (interquartile range, IQR), or unweighted number (percentage, %), unless otherwise specified. This table primarily presents the universal core variables harmonized across all datasets; cohort‐specific variables are detailed in the respective Supplementary Tables. Smoking and alcohol drinking statuses were uniformly dichotomized into ‘Ever’ (encompassing both current and former users) and ‘Never’. “NA” indicates that the variable was not available or not collected in the respective database. For the NHANES cohort, to account for the complex, multistage probability sampling design, all means and standard errors (SEs) for continuous variables, as well as percentages for categorical variables, were weighted. Statistical evaluations for NHANES utilized the Wilcoxon rank‐sum test for complex survey samples and the chi‐squared test with Rao & Scott's second‐order correction.

*The total pooled sample size (*N* = 70,350) represents the sum of the five non‐overlapping baseline samples (CHARLS cross‐sectional, ELSA, NHANES, Ganzhou, and Zhaotong). To strictly prevent participant duplication, the CHARLS longitudinal cohort (*N* = 5,927) was analyzed as an independent longitudinal validation set and is excluded from this pooled cross‐sectional sum.

BMI, body mass index; BUN, blood urea nitrogen; WBC, white blood cell; PLT, platelet count; MCV, mean corpuscular volume; TyG, triglyceride‐glucose index; hs‐CRP, high‐sensitivity C‐reactive protein; DM, diabetes mellitus; CPD, chronic pulmonary disease; CHD, coronary heart disease; CKD, chronic kidney disease; CHARLS, China Health and Retirement Longitudinal Study; ELSA, English Longitudinal Study of Ageing; NHANES, National Health and Nutrition Examination Survey.

Baseline characteristics according to cohort‐specific TyG quartiles are presented in Supplementary Tables S1–S6. Across cohorts, participants in higher TyG quartiles generally had higher BMI and a greater prevalence of hypertension, diabetes, and dyslipidemia. Higher TyG quartiles were also accompanied by differences in several hematologic, inflammatory, and metabolic laboratory measures, although the distributions varied by cohort.

### 3.2 Association Between the TyG Index and Anemia

In fully adjusted models, higher TyG index values were inversely associated with anemia across all six analytic cohorts (Table 2 and Figure 2). In the longitudinal analyses, each 1‐unit higher baseline TyG index was associated with lower odds of incident anemia in CHARLS (OR, 0.87; 95% CI, 0.77–0.98; *p* = 0.025) and ELSA (OR, 0.62; 95% CI, 0.45–0.86; *p* = 0.005).

**TABLE 2:** Association between the TyG index and anemia across six analytic cohorts.

Variables	CHARLS cross	CHARLS long	NHANES	ELSA	Ganzhou	Zhaotong
Total *N*/ Events *N* (%)	10732/1723 (16.10)	5927/1048 (17.70)	14559/1716 (8.01*)	2459/226 (9.20)	32395/2630 (8.10)	10205/847 (8.30)
TyG Continuous						
Model 4 (Fully adjusted)	0.76 (0.69‐0.84)	0.87 (0.77‐0.98)	0.61 (0.52‐0.71)	0.62 (0.45‐0.86)	0.53 (0.48‐0.58)	0.58 (0.49‐0.68)
*P*	< 0.001	0.025	< 0.001	0.005	< 0.001	< 0.001
TyG Quartiles						
Model 4 (Fully adjusted)	OR (95%CI)	OR (95%CI)	OR (95%CI)	OR (95%CI)	OR (95%CI)	OR (95%CI)
Q1(Ref)	1.00	1.00	1.00	1.00	1.00	1.00
Q2	0.77 (0.67‐0.89)	0.92 (0.76‐1.11)	0.80 (0.63‐1.00)	0.86 (0.59‐1.26)	0.62 (0.54‐0.70)	0.75 (0.60‐0.93)
Q3	0.67 (0.57‐0.78)	0.83 (0.68‐1.01)	0.64 (0.49‐0.82)	0.58 (0.38‐0.88)	0.51 (0.45‐0.59)	0.47 (0.37‐0.61)
Q4	0.63 (0.53‐0.75)	0.77 (0.62‐0.96)	0.44 (0.34‐0.58)	0.58 (0.37‐0.89)	0.39 (0.34‐0.46)	0.46 (0.34‐0.62)
*P* for trend	0.003	0.014	< 0.001	0.004	< 0.001	< 0.001

*In the descriptive row TyG index and anemia across six analytic cohortsLT, platelet count; MCV, mean weighted prevalence of anemia, whereas percentages for other cohorts are unweighted crude rates. Data are presented as Odds Ratios (OR) and 95% Confidence Intervals (CI). Model 4 represents the fully adjusted model; selection was informed by a DAG and tailored to each cohort, broadly encompassing sociodemographic factors, lifestyle habits, and comprehensive clinical comorbidities. For the longitudinal cohorts (CHARLS‐Longitudinal and ELSA), the ORs indicate the likelihood of *incident* anemia during the follow‐up period. For the cross‐sectional cohorts (CHARLS‐Cross‐sectional, NHANES, Ganzhou, and Zhaotong), the ORs indicate the likelihood of *prevalent* anemia. The lowest quartile (Q1) of the TyG index served as the reference group for categorical analyses. The *P* for trend was calculated by modeling the median value of each TyG quartile as a continuous variable.

TyG, Triglyceride‐Glucose; OR, Odds Ratio; CI, Confidence Interval; Ref, Reference; CHARLS, China Health and Retirement Longitudinal Study; ELSA, English Longitudinal Study of Ageing; NHANES, National Health and Nutrition Examination Survey.

**FIGURE 2:**
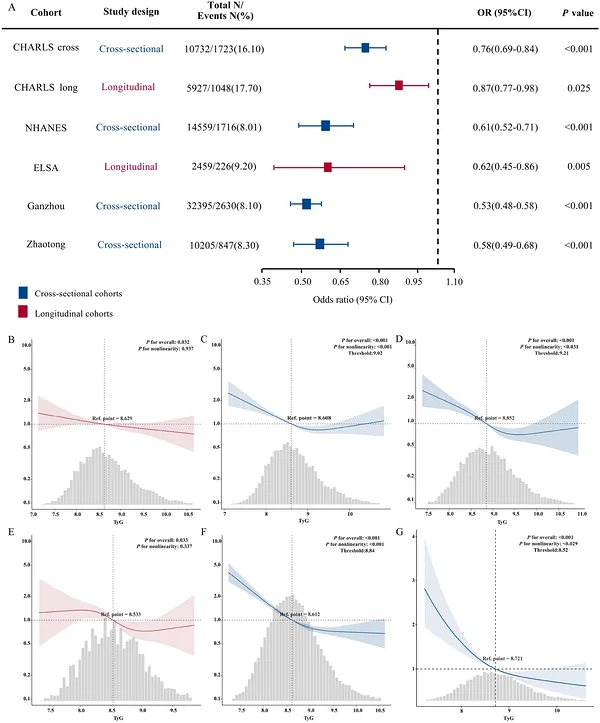
Associations and dose–response relationships between the TyG index and anemia across six analytic cohorts. (A) Forest plot showing the fully adjusted associations of the TyG index with prevalent anemia in the cross‐sectional cohorts and incident anemia in the longitudinal cohorts. Effect estimates are presented as ORs with 95% CIs per 1‐unit increase in the TyG index. (B–G) Restricted cubic spline analyses illustrating the dose–response relationships between the TyG index and anemia in CHARLS longitudinal (B), CHARLS cross‐sectional (C), Zhaotong (D), ELSA (E), Ganzhou (F), and NHANES (G). Splines were fitted using four knots at the fifth, 35th, 65th, and 95th percentiles of the TyG distribution and adjusted for the covariates included in the fully adjusted cohort‐specific models. Solid lines indicate adjusted ORs, shaded areas indicate 95% CIs, and gray histograms show the distribution of the TyG index. The horizontal dashed line represents OR = 1. P values for overall association and nonlinearity are displayed within each panel; estimated inflection points are shown for nonlinear associations. Blue denotes cross‐sectional cohorts and red denotes longitudinal cohorts. CI, confidence interval; CHARLS, China Health and Retirement Longitudinal Study; ELSA, English Longitudinal Study of Ageing; NHANES, National Health and Nutrition Examination Survey; OR, odds ratio; RCS, restricted cubic spline; TyG, triglyceride–glucose index.

Similar inverse associations were observed in the cross‐sectional cohorts, with fully adjusted ORs per 1‐unit higher TyG index of 0.76 (95% CI, 0.69–0.84) in CHARLS, 0.61 (95% CI, 0.52–0.71) in NHANES, 0.53 (95% CI, 0.48–0.58) in Ganzhou, and 0.58 (95% CI, 0.49–0.68) in Zhaotong (all *P* < 0.001). In categorical analyses, the fully adjusted ORs for the highest vs. lowest TyG quartile ranged from 0.39 to 0.77 across the six cohorts, with significant trends across quartiles in each cohort (Table 2; Supplementary Tables S7–S12).

### 3.3 Dose–response Associations

Restricted cubic spline analyses showed distinct dose–response patterns between the longitudinal and cross‐sectional cohorts (Figure 2). In the CHARLS longitudinal cohort (Figure 2B) and ELSA (Figure 2E), the inverse associations between the TyG index and incident anemia were approximately linear, with no evidence of nonlinearity (*P* for non‐linearity = 0.937 and 0.337, respectively).

In contrast, all four cross‐sectional cohorts—CHARLS cross‐sectional (Figure 2C), Zhaotong (Figure 2D), Ganzhou (Figure 2F), and NHANES (Figure 2G)—showed significant nonlinear inverse associations (all *P* for non‐linearity < 0.05). The odds of prevalent anemia decreased more markedly across the lower range of TyG values and subsequently flattened at higher levels. The estimated inflection points were 9.02 in CHARLS, 9.21 in Zhaotong, 8.84 in Ganzhou, and 8.52 in NHANES.

### 3.4 Extended Time‐to‐event Analysis in ElSA

In the extended ELSA follow‐up through Wave 8, 2,459 participants were included in the time‐to‐event analysis, among whom 266 developed incident anemia; cohort derivation and follow‐up status are shown in Supplementary Figure S8. The extended analysis yielded results consistent with the primary 4‐year longitudinal analysis. In the fully adjusted interval‐censored time‐to‐event model, each 1‐unit higher baseline TyG index was associated with a lower hazard of incident anemia (HR, 0.61; 95% CI, 0.43–0.87; *P* = 0.006; Supplementary Table S13). Quartile analyses showed a similar pattern, with HRs of 0.55 and 0.53 for Q3 and Q4, respectively, compared with Q1 (*P* for trend = 0.004). Restricted cubic spline analysis showed no evidence of nonlinearity (*P* for non‐linearity = 0.249; Supplementary Figure S9). The proportional‐hazards assumption was satisfied globally (*P* = 0.628; Supplementary Table S14). Results were also consistent when the analysis was restricted to participants with complete extended follow‐up (HR, 0.64; 95% CI, 0.47–0.88; *P* = 0.006; Supplementary Table S15).

### 3.5 Stratified Analyses

Stratified analyses in the longitudinal CHARLS and ELSA cohorts showed variation in the magnitude of the association between the TyG index and incident anemia across several demographic and clinical strata (Figure 3). In CHARLS, between‐stratum heterogeneity was observed for heart disease status (*P* for heterogeneity = 0.016), with adjusted ORs of 0.65 (95% CI, 0.43–1.00) among participants with heart disease and 0.90 (95% CI, 0.79–1.02) among those without heart disease. Heterogeneity was also observed across age strata (*P* for heterogeneity = 0.003), with ORs of 0.69 (95% CI, 0.54–0.88) among participants aged ≥ 65 years and 1.00 (95% CI, 0.87–1.16) among those aged < 65 years.

**FIGURE 3:**
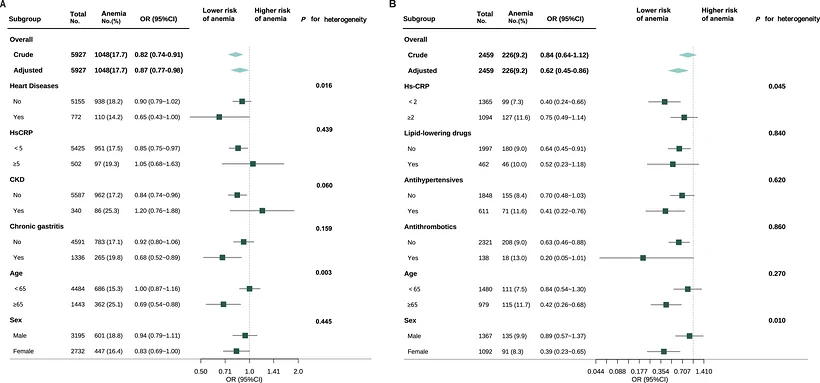
Stratified associations between the TyG index and incident anemia in the longitudinal CHARLS and ELSA cohorts. Forest plots show the adjusted associations of the TyG index with incident anemia across demographic and clinical strata in CHARLS (A) and ELSA (B). Effect estimates are presented as ORs with 95% CIs per 1‐unit increase in the TyG index. Models were adjusted for the covariates included in the fully adjusted cohort‐specific models, excluding the corresponding stratification variable where applicable. TyG was modeled continuously in these analyses, and between‐stratum heterogeneity was assessed using likelihood‐ratio tests comparing models with and without the corresponding continuous TyG‐by‐subgroup term. These analyses therefore evaluate heterogeneity in the per‐1‐unit TyG association and complement the formal interaction analyses based on median‐dichotomized TyG presented in Table 3 and Figure 4.

In the stratified analyses with TyG modeled continuously, the inverse association was observed among participants with hs‐CRP < 5 mg/L (OR, 0.85; 95% CI, 0.75–0.97) but not among those with hs‐CRP ≥ 5 mg/L (OR, 1.05; 95% CI, 0.68–1.63), although the between‐stratum heterogeneity test was not statistically significant (*P* = 0.439). In ELSA, the association differed across hs‐CRP strata (*P* for heterogeneity = 0.045), with ORs of 0.40 (95% CI, 0.24–0.66) for hs‐CRP < 2 mg/L and 0.75 (95% CI, 0.49–1.14) for hs‐CRP ≥ 2 mg/L. Differences in association magnitude were also observed across sex strata in ELSA (*P* for heterogeneity = 0.010), with ORs of 0.39 (95% CI, 0.23–0.65) in women and 0.89 (95% CI, 0.57–1.37) in men.

Complementary stratified analyses in the four cross‐sectional cohorts are presented in Supplementary Figure S10. The inverse association was generally preserved across the examined strata, although differences in association magnitude were observed for selected characteristics, including MCV in Ganzhou and age in Zhaotong.

Selected modifiers showing notable between‐stratum differences were subsequently evaluated using formal interaction analyses on both multiplicative and additive scales.

### 3.6 Analysis of Effect Modification

Systemic inflammation and heart disease were further examined using formal multiplicative and additive interaction analyses based on joint‐exposure models in which TyG was dichotomized at the cohort‐specific median (Table 3 and Figure 4).

**TABLE 3:** Formal interaction analyses of the TyG index with systemic inflammation and heart disease for incident anemia.

	CHARLS		ELSA
	hs‐CRP	Heart diseases	Hs‐CRP
Multiplicative interaction			
Multiplicative scale	1.71(1.06∼2.77)	0.61(0.39‐0.95)	1.88 (1.03‐3.46)
*P*	0.030	0.030	0.035
Additive interaction			
RERI (95% CI)	0.51 (0.05‐0.98)	−0.30(‐0.65‐0.04)	0.33 (‐0.17‐0.83)
AP (95% CI)	0.44 (0.12‐0.76)	−0.61(‐1.38‐0.16)	0.34 (‐0.17‐0.85)
SI (95% CI)	NA	NA	NA

For the formal interaction analyses, TyG was dichotomized at the cohort‐specific median and combined with the corresponding binary effect modifier to construct joint‐exposure categories. Multiplicative interaction was assessed using the product term between dichotomized TyG and the binary modifier in the fully adjusted logistic regression model. Additive interaction was evaluated using the relative excess risk due to interaction (RERI), attributable proportion due to interaction (AP), and synergy index (SI). Statistical support for RERI and AP was defined by a 95% CI excluding 0; no separate *p* value for additive interaction was calculated. SI could not be estimated for the reported comparisons and is therefore presented as NA. These analyses used a different exposure parameterization from the stratified analyses in Figure 3, in which TyG was modeled continuously.

hs‐CRP, high‐sensitivity C‐reactive protein; RERI, relative excess risk due to interaction; AP, attributable proportion due to interaction; SI, synergy index; CHARLS, China Health and Retirement Longitudinal Study; ELSA, English Longitudinal Study of Ageing

**FIGURE 4:**
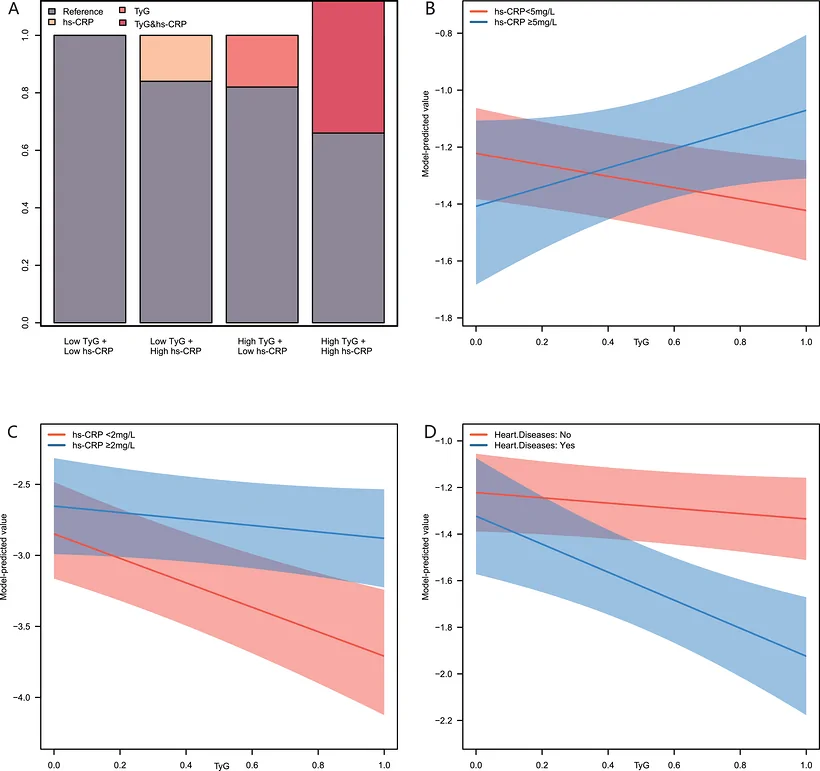
Interaction of the TyG index with systemic inflammation and heart disease in relation to incident anemia.(A) Joint associations of median‐dichotomized TyG and systemic inflammation with incident anemia in CHARLS, corresponding to the formal multiplicative and additive interaction analyses reported in Table 3. (B–C) Model‐based predicted values according to dichotomized TyG and hs‐CRP categories in CHARLS (B; < 5 vs ≥ 5 mg/L) and ELSA (C; < 2 vs ≥ 2 mg/L), illustrating the pattern of effect modification. (D) Model‐based predicted values according to dichotomized TyG and heart disease status in CHARLS. Formal multiplicative and additive interaction estimates were derived from the median‐dichotomized joint‐exposure models and are reported in Table 3. All models were adjusted for the covariates included in the fully adjusted cohort‐specific analyses. Lines represent model‐based predicted values. CHARLS, China Health and Retirement Longitudinal Study; CI, confidence interval; ELSA, English Longitudinal Study of Ageing; hs‐CRP, high‐sensitivity C‐reactive protein; TyG, triglyceride–glucose index.

For systemic inflammation, evidence of interaction was observed in CHARLS on both scales. The multiplicative interaction estimate for TyG and hs‐CRP was 1.71 (95% CI, 1.06–2.77; *P* = 0.030), while the additive interaction measures were RERI = 0.51 (95% CI, 0.05–0.98) and AP = 0.44 (95% CI, 0.12–0.76). In ELSA, a multiplicative interaction was also observed (1.88; 95% CI, 1.03–3.46; *p* = 0.035), whereas the corresponding additive interaction estimates were not statistically supported (RERI, 0.33; 95% CI, −0.17 to 0.83; AP, 0.34; 95% CI, −0.17 to 0.85). The joint‐exposure estimates underlying the additive interaction analyses are shown in Figure 4A. Consistent with these results, the predicted association curves showed attenuation of the inverse TyG–anemia association at higher hs‐CRP levels in both CHARLS and ELSA (Figure 4B,C).

For heart disease in CHARLS, interaction was supported on both multiplicative and additive scales. The multiplicative interaction estimate was 0.61 (95% CI, 0.39–0.95; *P* = 0.030), with a RERI of −0.30 (95% CI, −0.65 to −0.04) and an AP of −0.61 (95% CI, −1.38 to −0.16). The predicted association between TyG and incident anemia was more strongly inverse among participants with heart disease than among those without heart disease (Figure 4D).

### 3.7 Exploratory Pathway Analysis

Exploratory analyses were conducted in the CHARLS longitudinal cohort to evaluate potential intermediate pathways linking the TyG index with incident anemia (Figure 5 and Table S16). Candidate markers included cystatin C, hs‐CRP, WBC count, and CKD status. No statistically supported indirect association was observed for hs‐CRP, WBC count, or CKD status, with the corresponding 95% CIs for the indirect effects including zero. In contrast, the indirect association through cystatin C was supported (indirect effect, −0.0053; 95% CI, −0.0098 to −0.0011), statistically accounting for 16.33% of the observed association between the TyG index and incident anemia.

**FIGURE 5:**
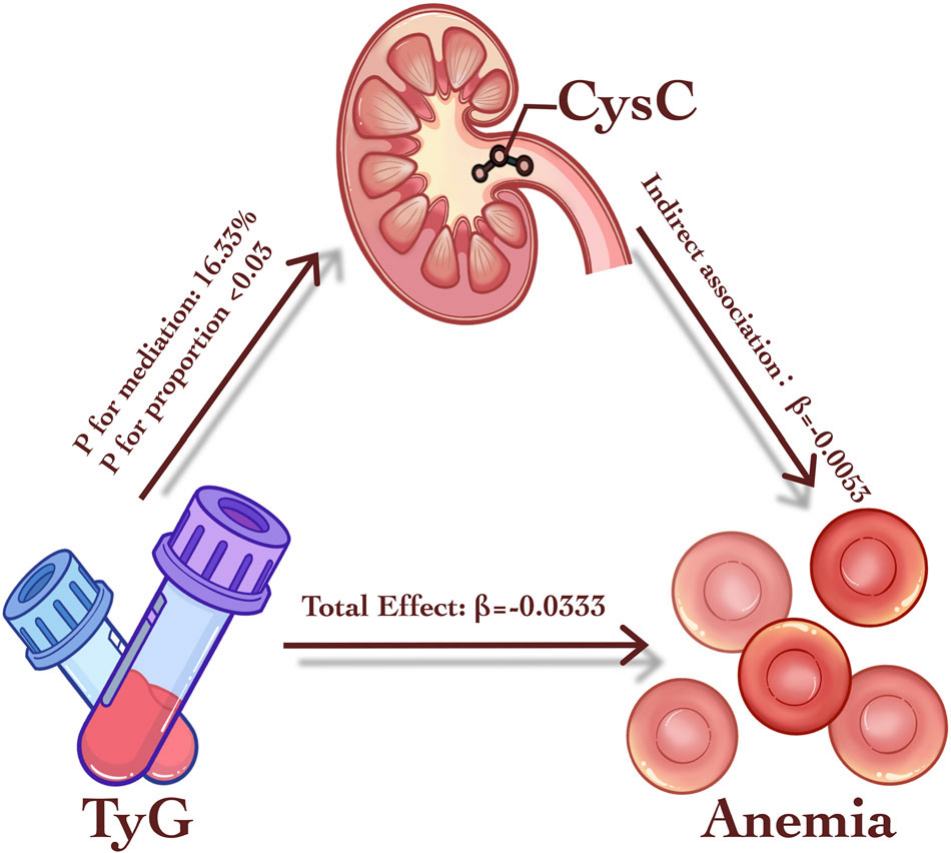
Exploratory mediation analysis of cystatin C in the association between the TyG index and incident anemia. The diagram shows the estimated total, direct, and indirect associations between the TyG index and incident anemia through cystatin C in the longitudinal CHARLS cohort. The indirect association through cystatin C was −0.0053 (95% CI, −0.0098 to −0.0011), corresponding statistically to 16.33% of the observed total association. Estimates were derived from models adjusted for the covariates included in the fully adjusted analysis. Because the TyG index and cystatin C were measured concurrently at baseline, this analysis does not establish temporal or causal mediation and should be interpreted as an exploratory pathway analysis. CHARLS, China Health and Retirement Longitudinal Study; CI, confidence interval; CysC, cystatin C; TyG, triglyceride–glucose index.

The estimated direct association remained inverse after accounting for cystatin C (direct effect, approximately −0.028), indicating that most of the observed TyG–anemia association was not statistically accounted for by cystatin C (Figure 5). Because the TyG index and cystatin C were measured concurrently at baseline, these findings were considered exploratory and do not establish a temporal or causal mediation pathway.

### 3.8 Sensitivity Analyses

When anemia was stratified by erythrocyte morphology, the inverse association with the TyG index was observed for microcytic (MCV < 80 fL) and normocytic anemia, whereas the association was not statistically supported for macrocytic anemia (MCV >100 fL) in either CHARLS or NHANES (Supplementary Tables S17–S18).

The longitudinal associations remained similar after excluding participants with underweight status, poor self‐rated health, or selected baseline comorbidities. In CHARLS, the fully adjusted ORs per 1‐unit higher TyG ranged from 0.86 to 0.88 across these restricted analyses (*P* = 0.024–0.041), while the corresponding ORs in ELSA ranged from 0.55 to 0.62 (*P* = 0.003–0.006; Supplementary Tables S19–S20).

The estimates were also similar across alternative data‐processing strategies. After excluding participants with TyG values in the upper and lower 0.5% of the cohort‐specific distributions, the fully adjusted ORs ranged from 0.51 to 0.85 across the six cohorts (all *P* ≤ 0.015; Supplementary Table S21). In complete‐case analyses, the corresponding ORs ranged from 0.48 to 0.87 (all *P* ≤ 0.041; Supplementary Table S22). When TyG was alternatively categorized into tertiles, inverse associations were again observed across all six cohorts, with *P* for trend ranging from < 0.001 to 0.011 (Supplementary Table S23). E‐values for the highest vs. lowest TyG quartiles ranged from 1.54 to 4.57 across the six cohorts (Supplementary Table S27).

Using METS‐IR as an alternative surrogate marker of insulin resistance yielded a similar inverse association with incident anemia in CHARLS (OR per 1‐unit increase, 0.98; 95% CI, 0.97–0.99; *P* = 0.001; Supplementary Table S24). However, METS‐IR did not show statistically supported interaction with hs‐CRP on either the multiplicative or additive scale, and no cystatin C‐related indirect association was observed (Supplementary Tables S25–S26).

In exploratory analyses stratified by baseline CKD status, the cystatin C‐related indirect association was supported among participants without CKD (indirect effect, −0.0052; 95% CI, −0.0094 to −0.0016), whereas it was not statistically supported among participants with CKD (indirect effect, −0.0082; 95% CI, −0.0262 to 0.0004; Supplementary Table S28). Because the total TyG–anemia association was close to null in the CKD subgroup (total effect, −0.0013), the proportion mediated was not interpreted for this subgroup.

Finally, additional adjustment for potential lifestyle, functional, nutritional, psychological, and treatment‐related confounders yielded estimates similar to those from the primary models. These analyses accounted, where available, for sleep and physical activity (Supplementary Tables S29–S31), frailty (Supplementary Table S32), dietary and nutritional factors and serum albumin (Supplementary Table S33), depressive symptoms (Supplementary Table S34), and cardiometabolic medication use (Supplementary Table S35).

## 4 Discussion

In this multinational multicohort study of 70,350 unique participants, a higher TyG index was consistently associated with lower odds of prevalent and incident anemia across six analytic cohorts—a direction notably different from the predominantly adverse prognostic significance attributed to elevated TyG in the cardiometabolic literature [8, 9]. This inverse association was reproduced across cross‐sectional and longitudinal designs, different observation periods, geographically distinct settings, and populations from China, the United States, and the United Kingdom, providing temporal, geographic, and population‐level epidemiological triangulation. Dose–response analyses showed approximately linear inverse associations in the two longitudinal cohorts but nonlinear associations that flattened at higher TyG levels in all four cross‐sectional cohorts. The association was evident for microcytic and normocytic anemia, attenuated at higher systemic inflammation, and stronger among participants with heart disease. Exploratory analyses further identified a cystatin C–related indirect association, particularly among participants without CKD. Collectively, these findings suggest that the physiological implications of a higher glucose–lipid metabolic state may be outcome‐ and context‐dependent rather than uniformly adverse.

This directional contrast with the broader TyG literature is particularly noteworthy. TyG was originally developed as a surrogate of insulin resistance and has subsequently been used predominantly as a marker of adverse cardiometabolic risk [8, 9, 22]. Our findings do not challenge this established role, but suggest that the metabolic phenotype captured by TyG may have different implications across physiological systems. Erythropoiesis is an energy‐ and growth‐factor‐dependent process, and insulin and IGF‐1 signaling have established erythropoiesis‐supporting effects [11, 12, 13]. Consistent with this concept, previous studies have linked obesity or metabolic syndrome with hemoglobin levels [14, 15], and a recent CHARLS study reported inverse associations of several insulin‐resistance surrogates with anemia [16]. Our study extends these observations through longitudinal and multinational replication. In this context, TyG may be viewed not merely as a numerical surrogate of insulin resistance, but as an integrative marker of the prevailing glucose–lipid metabolic phenotype.

The dose–response patterns add another dimension to this association. The longitudinal cohorts showed approximately linear relationships, whereas the cross‐sectional cohorts consistently exhibited nonlinear curves that flattened at higher TyG levels. This difference may partly reflect the distinct temporal information captured by the two designs: cross‐sectional analyses represent the contemporaneous metabolic–hematological state and are therefore more susceptible to current nutritional status, disease burden, treatment, and reverse causation, whereas longitudinal analyses relate baseline metabolic status to subsequent anemia development. Biologically, the cross‐sectional flattening may be compatible with diminishing marginal relevance of additional glucose–lipid availability once metabolic substrate and anabolic signaling are no longer major constraints on erythropoiesis, consistent with the erythropoiesis‐supporting effects of insulin and IGF‐1 signaling [11, 12, 13]. Nevertheless, these curves should be interpreted as epidemiological dose–response patterns rather than evidence of a physiological threshold or an optimal TyG range.

The association also varied according to anemia phenotype. The inverse relationship with TyG was consistently observed for microcytic and normocytic anemia, whereas it was substantially attenuated for macrocytic anemia. This pattern is clinically relevant because anemia represents a heterogeneous group of disorders rather than a single biological entity. Microcytic and normocytic phenotypes are commonly encountered in iron‐restricted states, chronic disease, inflammation, and renal dysfunction [3, 4, 5, 6, 7], whereas macrocytosis more often reflects distinct mechanisms such as vitamin B12 or folate deficiency, marrow disorders, or other abnormalities less directly linked to glucose–lipid metabolism [1]. The phenotype‐specific pattern therefore suggests that the TyG–anemia association may be more relevant to forms of anemia arising within metabolic, inflammatory, or cardiorenal contexts than to anemia driven by fundamentally different hematopoietic mechanisms. However, MCV alone cannot establish anemia etiology, and these findings should be interpreted as evidence of clinical heterogeneity rather than etiological specificity.

The context dependence of the TyG–anemia association was particularly evident for systemic inflammation and heart disease. Higher hs‐CRP levels were associated with attenuation of the inverse relationship, with formal interaction analyses providing additional support for effect modification. This pattern is biologically plausible because systemic inflammation can impair erythropoiesis through hepcidin‐mediated iron restriction, reduced iron availability, and diminished erythropoietic responsiveness [3, 30]. Under such conditions, the contribution of metabolic substrate availability and anabolic signaling to erythropoiesis may become less prominent. Importantly, our findings indicate attenuation rather than a true reversal of the association. In contrast, the inverse association was stronger among participants with heart disease in CHARLS. Cardiovascular disease frequently coexists with anemia, renal dysfunction, and chronic catabolic stress within the cardiorenal continuum [5, 6, 7], potentially increasing the relevance of metabolic state to hematopoietic homeostasis. Nevertheless, these findings should be interpreted as evidence of clinical effect modification rather than proof of a compensatory biological mechanism.

The exploratory cystatin C analysis provides a further clue to a potential renal‐function‐related pathway. Cystatin C is a sensitive marker of kidney function and may capture clinically relevant variation in filtration status beyond that reflected by serum creatinine alone [17, 18]. In the overall CHARLS cohort, an indirect association through cystatin C was observed, and this pattern remained evident among participants without baseline CKD but was not statistically supported in those with CKD. Given the close physiological link between renal function and erythropoiesis, particularly through renal erythropoietin production, this finding provides a plausible basis for further investigation [5, 7, 31]. However, cystatin C is not a direct measure of glomerular hyperfiltration, renal hemodynamics, or erythropoietin activity, and TyG and cystatin C were measured concurrently at baseline. Therefore, the temporal sequence required for causal mediation cannot be established. These results should consequently be interpreted as evidence supporting a possible renal‐function‐related pathway rather than proof of renal hemodynamic mediation. The differing pattern by CKD status further suggests that the metabolic–hematological association may depend on underlying renal context, but does not establish that preserved renal function is required for the association to occur.

### 4.1 Strengths of the Study

This study has several strengths. First, the primary association was evaluated in 70,350 unique participants across six analytic cohorts encompassing population‐based surveys and contemporary clinical populations from China, the United States, and the United Kingdom. The combination of cross‐sectional and longitudinal designs, different observation periods, and geographically and demographically distinct populations provided temporal, geographic, and population‐level triangulation of the findings. Second, the longitudinal evidence was supported by both harmonized follow‐up analyses and an extended time‐to‐event analysis, reducing dependence on a single analytical framework. Third, the consistency and boundaries of the association were examined across multiple complementary dimensions, including dose–response patterns, anemia morphology, systemic inflammation, cardiovascular and renal contexts, and an alternative insulin‐resistance surrogate. Finally, extensive sensitivity analyses addressing reverse causation, missing data, extreme values, lifestyle factors, frailty, nutritional status, depressive symptoms, medication use, and unmeasured confounding yielded findings broadly consistent with the primary analyses.

### 4.2 Limitations

Several limitations should be considered. First, although the longitudinal and extended time‐to‐event analyses established temporal ordering between baseline TyG and subsequent anemia, the observational nature of the study does not allow causal relationships to be established. Second, TyG is a surrogate of insulin resistance and an integrative marker of glucose–lipid metabolism rather than a direct measure of insulin action or “metabolic reserve”; the latter therefore remains a conceptual interpretation. Third, detailed anemia etiology was not uniformly available across cohorts. Iron indices, vitamin B12, folate, reticulocyte measures, and other hematological assessments were incomplete or unavailable in several datasets, and MCV classification cannot establish etiology. Fourth, covariate availability differed across cohorts. Although major lifestyle, nutritional, functional, psychological, and medication‐related factors were additionally evaluated where possible, residual confounding remains possible. Fifth, TyG and cystatin C were measured concurrently at baseline, and glomerular hyperfiltration, renal hemodynamics, erythropoietin, and related renal mechanisms were not directly assessed; the cystatin C findings should therefore be regarded as exploratory pathway evidence rather than causal mediation. Finally, anemia was assessed at discrete study waves, and the cross‐sectional analyses remain susceptible to reverse causation and contemporaneous disease effects.

## 5 Conclusions

Across six multinational cohorts, higher TyG was consistently associated with a lower likelihood of prevalent and incident anemia, revealing a metabolic–hematological relationship that contrasts with the well‐established adverse cardiovascular implications of elevated TyG. The association was modified by anemia phenotype, systemic inflammation, cardiovascular disease, and renal context, supporting the concept that the physiological consequences of glucose–lipid metabolic status are not uniform across organ systems. These findings do not suggest that higher TyG or insulin resistance should be therapeutically maintained. Rather, they highlight that intensive glucose‐ and lipid‐lowering strategies should consider hematological status alongside vascular risk, particularly in older, frail, or cardiovascularly compromised individuals. New‐onset or worsening anemia during aggressive metabolic control may therefore warrant evaluation not only for conventional causes but also as a potential marker of diminishing metabolic reserve.

## Author Contributions

GSJ: Conceptualization, CHARLS and ELSA data analysis, and manuscript writing. XXW: NHANES data processing and visualization. LFX, LJZ, and ZW collected the Ganzhou cohort data. WND: Visualization. YYY, MY, ZBC, and LL: Zhaotong cohort data collection. KQT: ELSA data analysis. WJ: Literature search. XXQ: Technical support, data analysis, and visualization. CEQ: Conceptualization, manuscript review, and editing. All the authors have read and approved the final manuscript.

## Funding

This study did not receive any funding.

## Conflicts of Interest

All authors certify that they have no affiliations with or involvement in any organization or entity with any financial or non‐financial interest in the subject matter or materials discussed in this manuscript.

## Ethics approval statement

This Study Was Conducted in Accordance With the Principles of the Declaration of Helsinki. Ethics approval For the CHARLS Cohort Was Granted By the Biomedical Ethics Review Committee of Peking University (No. IRB00001052‐11015), and the ELSA Study Was Approved By the London Multicenter Research Ethics Committee (MREC/01/2/91). The NHANES Protocol Was Reviewed and Approved By the NCHS Research Ethics Review Board. For hospital‐Based Cohorts, the Protocols Were Approved By the Ethics Committees of Yunnan Provincial Diandongbei Central Hospital (No. XSD2026023) and the First Affiliated Hospital of Gannan Medical University (No. LLSL‐2026147).

## Patient consent statement

Written informed consent was obtained from all participants of the CHARLS, ELSA, and NHANES cohorts. Regarding the Hospital‐Based Clinical Analysis, given its retrospective nature, utilizing strictly de‐identified, pre‐existing electronic medical records, with no direct patient contact and re‐identification impossible, the requirement for informed consent was waived by the respective Ethics Committees in accordance with the Declaration of Helsinki.

## Supporting information




**Supporting File**: advs78037‐sup‐0001‐SuppMat.docx.

## Data Availability

This data can be found at CHARLS: https://charls.charlsdata.com/pages/data/111/zh‐cn.html, NHANES: https://wwwn.cdc.gov/nchs/nhanes, and ELSA: https://www.elsa‐project.ac.uk/. Data from the Hospital‐Based Cohorts (Northeast Yunnan Central Hospital and the First Affiliated Hospital of Gannan Medical University) Are Not Publicly Available Due to Patient Privacy Regulations and Ethical restrictions. De‐identified data May Be Available from the Corresponding Author Upon Reasonable Request and May Be Subject to Ethical Approval.
