# Supplementary material for: Context‐Dependent Inverse Association of Glucose–Lipid Metabolic Status With Anemia: Evidence From Multinational Multicohort Analyses
Source: Adv Sci (Weinh). 2026 Sep 29:e78037. Online ahead of print. doi: 10.1002/advs.78037 (PMC13624305; doi:10.1002/advs.78037)
Supplement: Supplementary file 1 [file ADVS-9999-e78037-s001.docx]

**Supplementary Material**

**Title:** Context-Dependent Inverse Association of Glucose–Lipid Metabolic Status with Anemia: Evidence from Multinational Multicohort Analyses

**Authors:** Guosong Jiang^†^, Xinxing Wang^†^, Lianfeng Xie^†^, Wenna Ding, Yingyong Yin, Mei Yao, Zhibo Cui, Kaiqiang Tang, Lijie Zhong, Zhi Wang, Jia Wang, Lei Lei, Xiaoxiao Qu*, Chi-E Qiu*

Supplementary Figure S1. Directed Acyclic Graph (DAG) for covariate selection and exploratory
mediation pathway	4

Detailed definitions and measurement protocols of covariates	5-18

Supplementary Figures S2–S7. Cohort-Specific Model Validation and Diagnostics	19-22

Supplementary Tables S1–S6. Detailed Baseline Characteristics Stratified by TyG Index Quartiles 23-32

Supplementary Tables S7–S12. Stepwise Multivariable Regression Analyses for the Association
Between the TyG Index and Anemia	33-38

**Extended Time-to-Event Analyses in the ELSA Cohort**

Supplementary Figure S8. Flowchart of participant selection and censoring for the extended
time-to-event analysis in the ELSA cohort	39

Supplementary Table S13. Association between the TyG index and incident anemia in the extended
ELSA time-to-event analysis	40

Supplementary Figure S9. Dose-response association between the TyG index and incident anemia in
the extended ELSA time-to-event analysis	41

Supplementary Table S14. Proportional hazards assumption assessment for the interval-censored

proportional hazards model	42

Supplementary Table S15. Complete-follow-up sensitivity analysis of the association between the
TyG index and incident anemia in the ELSA cohort	43

**Stratified Analyses**

Supplementary Figure S10. Stratified analyses of the association between the TyG index and the
risk of prevalent anemia across four cross-sectional cohorts	44

**Exploratory Mediation and Pathway Analyses**

Supplementary Table S16. Exploratory comparison of candidate intermediate markers in the association

between the TyG index and incident anemia in the CHARLS longitudinal cohort	45

**Sensitivity Analyses Stratified by Anemia Morphology (MCV Categories)**

Supplementary Tables S17–S18. Sensitivity analyses of the association between the TyG index and
anemia stratified by MCV categories	46-48

**Sensitivity Analyses by Excluding Specific High-Risk or Morbid Subpopulations**

Supplementary Tables S19–S20. Sensitivity analyses of the association between the TyG index and
incident anemia after excluding specific high-risk or morbid subpopulations	49-50

**Sensitivity Analyses Regarding Data Processing and Outlier Management**

Supplementary Table S21. Sensitivity analysis after excluding extreme TyG values	51

Supplementary Table S22. Sensitivity analysis utilizing a complete-case approach	52

Supplementary Table S23. Sensitivity analysis utilizing tertile categorization of the TyG index	53

**Sensitivity Analyses Utilizing an Alternative Surrogate Marker for Insulin Resistance (METS-IR)**

Supplementary Table S24. Multivariable regression analysis of the association between METS-IR
and incident anemia in the CHARLS longitudinal cohort	54

Supplementary Table S25. Interaction analysis of METS-IR and systemic inflammation (hs-CRP) on
the risk of incident anemia	55

Supplementary Table S26. Exploratory mediation analysis of cystatin C in the association between
METS-IR and incident anemia	55

**Sensitivity Analysis for Unmeasured Confounding**

Supplementary Table S27. E-values for the association between the TyG index and the risk of
anemia across all six independent cohorts	56

**Exploratory Mediation Analysis by Baseline Renal Function**

Supplementary Table S28. Exploratory mediation analysis of cystatin C in the association between
the TyG index and incident anemia stratified by baseline chronic kidney disease status	57

**Sensitivity Analyses with Additional Lifestyle, Nutritional, Frailty, Psychological, and Medication Covariates**

Supplementary Table S29. Sensitivity analyses additionally accounting for sleep and physical
activity in the CHARLS longitudinal cohort	58

Supplementary Table S30. Sensitivity analyses additionally accounting for sleep and physical
activity in the ELSA cohort	59

Supplementary Table S31. Sensitivity analyses additionally accounting for sleep and physical
activity in the NHANES cohort	60

Supplementary Table S32. Sensitivity analysis of the association between the TyG index and
anemia after additional adjustment for frailty	61

Supplementary Table S33. Sensitivity analyses additionally accounting for dietary intake,
nutritional status, and serum albumin in the NHANES cohort	62

Supplementary Table S34. Sensitivity analysis of the association between the TyG index and
incident anemia after additional adjustment for depressive symptoms in the ELSA cohort	63

Supplementary Table S35. Sensitivity analysis of the association between the TyG index and
anemia after additional adjustment for cardiometabolic medications in the CHARLS cohorts	64

Supplementary Figure S1. Directed Acyclic Graph for Covariate Selection and the Exploratory Cystatin C Pathway.

The DAG summarizes the conceptual relationships among the TyG index, anemia, baseline covariates, and cystatin C. TyG was treated as the primary exposure and anemia as the outcome. Sociodemographic, lifestyle, clinical, and laboratory variables were considered candidate covariates and informed cohort-specific multivariable adjustment according to data availability. Cystatin C was represented separately as a candidate renal-function-related intermediate marker and was not included in the primary adjustment models. Its potential role was evaluated only in exploratory pathway analyses. The DAG was used to guide covariate organization and does not establish a causal mediation pathway.

China Health and Retirement Longitudinal Study (CHARLS)

1.Marital status

Marital status is defined as: Married (YES), living with one's spouse or not living with one's spouse; Not Married (No), divorced, widowed, or never married.

2.Educational status

Educational level is defined as follows: Illiterate: Did not complete primary school; Primary school: Graduated from primary school; Junior high school: Graduated from junior high school; High school or above: High school, vocational school, college, university and above.

3.Race

Non-Han: Minority groups / Others; Han

4.Residence

Urban community; Rural village

5.Smoking status

Never (Never smoke): No; Past (Have smoked in the past): Yes and have quit smoking; Current (Currently smoking): Yes and have not quit smoking.

6.Drinking status

Never (Never drink alcohol): No; Past (Have drunk alcohol in the past): Did not drink last year but did drink before; Current (Currently drinking alcohol): Yes.

7.Hypertension

Hypertension diagnosis was based on a self-reported physician-diagnosed, and/or any antihypertensive medication use, and/or an average systolic/diastolic blood pressure (SBP/DBP) ≥ 140/90 mmHg.

8.DM

DM was defined as FPG ≥ 126 mg/dl or HbA1c ≥ 6.5%, and/or a self-reported physician-diagnosed, and/or taking hypoglycemic agents. Pre-DM was characterized by an FPG of 100 to 125 mg/dL or an HbA1c of 5.7–6.4%.

9.Cancer

The diagnosis of cancer was made based on the self-reports provided by the participants.

10.CPD

CPD is defined as "Have you ever been informed by a doctor that you have a chronic lung disease, such as chronic bronchitis or emphysema (excluding asthma and tumors)? "

11.Heart Diseases

The diagnosis of heart diseases was made based on the self-reports provided by the participants. Has a doctor ever told you that you have heart attack, coronary heart disease, angina, congestive heart failure, or other heart problems?"

12.Stroke

Stroke was defined according to self-reported physician diagnosis and was coded as a binary variable (yes/no).

13. Depressive

Depressive Symptoms Depressive symptoms were assessed using the 10-item short form of the Center for Epidemiological Studies Depression Scale (CES-D 10), which has been validated for use in the Chinese population within the CHARLS framework. Participants were asked about the frequency of 10 symptoms (e.g., feeling depressed, fearful, or having restless sleep) experienced during the past week. Responses were recorded on a 4-point scale: rarely or none of the time (<1 day), some or a little of the time (1–2 days), occasionally or a moderate amount of the time (3–4 days), and most or all of the time (5–7 days). After reverse-scoring the two positive affect items ("I felt hopeful about the future" and "I was happy"), a total score was calculated, ranging from 0 to 30. Following established literature, a cut-off score of $\ge 10$ was utilized to define the presence of significant depressive symptoms (0 = no, 1 = yes).

14.Arthritis

Arthritis Arthritis status was determined based on self-reported physician diagnosis. Participants were asked the following question: "Have you ever been diagnosed by a doctor with arthritis or rheumatism (including osteoarthritis but excluding rheumatoid arthritis)?". Responses were coded as a binary variable (1 = yes, 0 = no).

15.Dyslipidemia

Dyslipidemia was diagnosed by a self-reported physician-diagnosed, and/or current use of lipid-lowering drugs, and/or total cholesterol (TC) ≥ 240 mg/dl, triglyceride (TG) ≥ 150 mg/dl, high-density lipoprotein cholesterol (HDL-C) < 40 mg/dl, low-density lipoprotein cholesterol (LDL-C) ≥ 160 mg/dl.

16.Liver Diseases

Liver Diseases The presence of liver diseases was identified through self-reported physician diagnosis. Participants were asked: "Have you been diagnosed with liver diseases (except for tumor or cancer, including fatty liver, hepatitis, and cirrhosis) by a doctor?" Responses were coded as a binary variable (1 = yes, 0 = no). This measure encompasses a broad spectrum of chronic hepatic conditions, including chronic hepatitis, fatty liver, and cirrhosis.

17.CKD

CKD was defined according to self-reported physician diagnosis and was coded as a binary variable (yes/no).

18.Chronic gastritis

Chronic gastritis was defined as a binary variable based on self-reported physician diagnosis. It was derived from the affirmative response to the survey question: “Have you been diagnosed with chronic gastritis (except for tumor or cancer) by a doctor?” (Yes = 1, No = 0).

19.BMI

Body mass index (BMI) was calculated as the following formula: weight/height^2^ (kg/m^2^).

20. Blood-based Biomarkers

Venous blood samples were collected from participants by medically trained staff following standard protocols. Hematological parameters, including White Blood Cell count (WBC), Mean Corpuscular Volume (MCV), and Platelet count (PLT), were analyzed using automated hematology analyzers. For biochemical indicators, Blood Urea Nitrogen (BUN) and Creatinine were measured to assess renal function. High-sensitivity C-reactive Protein (hs-CRP) was utilized as a marker of systemic inflammation. Additionally, Glycated Hemoglobin (HbA1c) was measured to evaluate long-term glycemic control. All laboratory assays were performed at the Centers for Disease Control and Prevention (CDC) in China.

21. Sleep duration

Nighttime sleep duration was assessed using the self-reported question: *“During the past month, how many hours of actual sleep did you get at night (average hours for one night)?”* Responses were recorded as the average number of hours slept per night. This measure reflects actual sleep time rather than time spent in bed.

22. Sleep quality

Sleep quality was derived from the sleep-related item of the CHARLS questionnaire assessing the frequency of restless or poor sleep during the preceding week. Responses followed the four-level frequency structure used in CHARLS: rarely or none of the time (<1 day), some or a little of the time (1–2 days), occasionally or a moderate amount of the time (3–4 days), and most or all of the time (5–7 days), corresponding to scores of 0–3, with higher scores indicating poorer sleep quality

23. Physical activity

Physical activity was assessed using the CHARLS physical activity questionnaire, which follows the structure of the International Physical Activity Questionnaire. Participants reported whether they engaged in vigorous-intensity activity, moderate-intensity activity, or walking/light-intensity activity for at least 10 consecutive minutes during a usual week. For each activity intensity, information on weekly frequency and usual duration was collected. Activity duration was recorded in predefined intervals and converted to minutes using the corresponding interval values. Weekly physical activity volume was derived using standard metabolic equivalent (MET) coefficients of 8.0 for vigorous activity, 4.0 for moderate activity, and 3.3 for walking/light activity.

24. Frailty index

Frailty was quantified using a 32-item deficit-accumulation Frailty Index (FI). Each health deficit was coded on a scale from 0 (absence of deficit) to 1 (maximum deficit), with intermediate values assigned where applicable. The FI was calculated as the sum of observed deficit scores divided by the maximum possible score for the non-missing items:

Participants were required to have valid information for at least 26 of the 32 prespecified deficits (≥80%) for calculation of the FI. Cognitive function was incorporated as a continuous deficit. The cognitive raw score was calculated as the mean of immediate and delayed word-recall scores plus the orientation score, yielding a range of 0–14; the corresponding cognitive deficit was calculated as. Higher FI values indicate greater accumulation of health deficits. The FI was modeled continuously in the sensitivity analyses.

English Longitudinal Study of Ageing (ELSA)

1.Race:

Ethnicity was categorized into White (including British, Irish, and any other White background) and Non-white (including Mixed, Black, Asian, and other groups).

2.Educational status:

Participant’s highest level of education was classified into three levels: High (university or higher degree), Intermediate (A-levels, O-levels, or equivalent), and Low (foreign or no formal qualifications).

3.Marital status:

This was defined as Married/Partnered, Divorced/Separated, Widowed, or Never married.

4.Smoking status:

Participants were classified as never smokers, former smokers, or current smokers based on self-reported history.

5.Drinking status:

Alcohol consumption frequency was assessed and categorized into non-drinkers/rarely, moderate drinkers, and heavy drinkers (e.g., more than 5 days per week).

6.Body Mass Index (BMI):

BMI was calculated as weight in kilograms divided by height in meters squared (BMI = kg/m^2^). It was analyzed either as a continuous variable or categorized (Normal: <25, Overweight: 25–29.9, Obese: ≥30).

7. Cardiovascular diseases:

These include hypertension, stroke, and heart disease (including myocardial infarction, heart failure, or angina pectoris). All were determined based on self-reported medical diagnosis histories by doctors.

8.Diabetes Mellitus (DM) was identified by a doctor’s diagnosis or a glycated hemoglobin (HbA1c) level ≥ 6.5%.

9. Chronic respiratory diseases:

Include chronic lung diseases (such as chronic bronchitis or emphysema). Other conditions: Include cancer (any malignant tumor, excluding skin cancer) and arthritis (osteoarthritis or rheumatoid arthritis). All of these conditions are determined based on the medical diagnosis history recorded by the doctor himself.

10.Medication use was recorded during the nurse interview and identified by the following categories:

Antihypertensive medication:

Use of ACE inhibitors, beta-blockers, or diuretics to control blood pressure.

Drug treatment for diabetes:

Usage of oral hypoglycemic agents or insulin.

Respiratory medications:

Including medication for CPD and medication for Asthma.

Cardiovascular-related drugs:

Including medications that thin the blood (antiplatelets or anticoagulants) and medication to treat high cholesterol (statins).

11.Blood samples were collected by qualified nurses during the home visits.

High-sensitivity C-reactive Protein (hs-CRP):

Measured as a marker of systemic inflammation (mg/L).

White Blood Cell (WBC) count: Used as a leukocyte marker of inflammatory status (10^9^/L).

Glycated Hemoglobin (HbA1c):

Measured to reflect average plasma glucose levels over the previous 8–12 weeks (%).

12. Sleep duration:

Sleep duration was assessed using the open-ended question asking participants how many hours of sleep they obtained on an average weeknight. The number of self-reported hours was used to characterize habitual nighttime sleep duration. Sleep-related questions were introduced at ELSA Wave 4 and subsequently repeated in later waves.

13.Sleep quality:

Sleep quality was assessed using four sleep-related items covering difficulty falling asleep, difficulty remaining asleep, waking up feeling tired, and overall/disturbed sleep during the previous month. Each item was scored from 1 to 4 according to frequency, ranging from no difficulty/not during the past month to three or more times per week. Item scores were summed to yield a sleep-disturbance score ranging from 4 to 16, with higher scores indicating poorer sleep quality or greater sleep disturbance

14. Physical activity

Physical activity was assessed from self-reported frequency of participation in vigorous, moderate, and mild sports or other physical activities. For each intensity, participants reported activity frequency as more than once a week, once a week, one to three times a month, or hardly ever/never. The standard ELSA physical activity measure combines these responses to characterize overall activity level, with higher categories reflecting more frequent and/or more vigorous activity.

15. Frailty index

Frailty was assessed using the same 32-item deficit-accumulation FI framework applied in CHARLS. Each deficit was scored from 0 to 1 and the FI was calculated as the sum of observed deficit scores divided by the number of deficits with valid information. Calculation required valid data for at least 26 of the 32 deficits (≥80%). Cognitive function was incorporated using the mean of immediate and delayed word recall plus orientation, yielding a score from 0 to 14;. Higher FI values indicate greater deficit accumulation. The FI was modeled as a continuous variable.

16. Depressive symptoms

Depressive symptoms were assessed using the 8-item Center for Epidemiologic Studies Depression Scale (CES-D) used in ELSA. The eight items assess whether participants experienced depressive affect, effort, restless sleep, reduced positive affect, loneliness, sadness, and difficulty getting going during the preceding week. Items are coded dichotomously, with positively worded items reverse-coded, and summed to yield a total score ranging from 0 to 8; higher scores indicate a greater depressive symptom burden. ELSA commonly uses a score of ≥4 to identify elevated depressive symptoms; however, in the present sensitivity analysis, the CES-D score was entered as a continuous variable rather than used to define clinical depression.

National Health and Nutrition Examination Survey (NHANES)

1.CKD：

Chronic Kidney Disease (CKD) cases were defined as individuals with an eGFR < 60 mL/ (min*1.73 m^2^) and eGFR was calculated using the CKD Epidemiology Collaboration (CKD-EPI) creatinine equation. The enrolled participants were divided into non-CKD group with eGFR ≥ 60 mL/ (min*1.73 m^2^) and CKD group with eGFR < 60 mL/ (min*1.73 m^2^).

2.COPD：

The diagnosis of COPD was established based on one or more of the following criteria: (1) a post-bronchodilator FEV1/FVC ratio < 0.70; (2) a diagnosis of emphysema confirmed by a physician or other healthcare professional; or (3) for individuals aged 40 years or older, a history of smoking combined with chronic bronchitis and treatment with selective phosphodiesterase-4 (PDE-4) inhibitors, mast cell stabilizers, leukotriene modifiers, or inhaled corticosteroids.

3.arthritis：

The diagnosis of arthritis is derived from the variable MCQ160A: Has a doctor ever told you that you have arthritis?

4. Definitions of dietary folate, dietary iron, and dietary vitamin B12:

(1) All used on the first day (DRXTOT)

(2) The purpose of the dietary interview component is to obtain detailed dietary intake information from the NHANES examination sample. All examinees are eligible to participate in this part. Dietary intake data are used to estimate the total intake of energy, nutrients, and non-nutrient food components from foods and beverages within the 24 hours prior to the interview (midnight to midnight). After the dietary recall, a brief "post-recall" questionnaire is administered; respondents estimate their intake of plain water in the past 24 hours and the frequency of consumption of fish and shellfish in the past 30 days. Two data files are generated from the dietary interview dataset: the total nutrient intake file (DRXTOT), which includes total nutrient intake, plain water intake, and frequency data for fish and shellfish; and the individual food file (DRXIFF), which includes detailed information on individual foods reported by respondents. The nutrient intakes reported in these files do not include nutrients obtained from dietary supplements, medications, or regular drinking water.

5.Drink：

Current drinking status includes: light drinking (≤1 drink per day for women, ≤2 drinks per day for men); moderate drinking (≥2 drinks per day for women, ≥3 drinks per day for men, or binge drinking ≥2 days per month); heavy drinking (≥3 drinks per day for women, ≥4 drinks per day for men, or binge drinking on ≥5 days per month [≥4 drinks per occasion for women, ≥5 drinks per occasion for men]).

6.Race

non-Hispanic White, non-Hispanic Black, mexican American, other Hispanic, other Race-Including Multi-Racial.

7.Marry

(1) Married/ Living with partner, and (2) Never married/Other: widowed, divorced, or separated individuals.

8.Education

Education levels were classified as less than 9th grade, 9-11th Grade (Includes 12th grade with no diploma), high School Grad/GED or equivalent, some College or AA degree and college graduate or above

9.Asthma：

The definition of asthma comes from variable MCQ010. Have you been informed whether you have asthma?

10. Heart disease:

Heart disease was defined as: A self-reported prior diagnosis of coronary heart disease, angina pectoris, myocardial infarction, or congestive heart failure, with the presence of any one of these conditions constituting a confirmed diagnosis.

11.Depression:

The Patient Health Questionnaire-9 (PHQ-9) is a validated 9-item screening tool used to assess the severity of depressive symptoms experienced over the preceding 2 weeks . Numerous studies have confirmed its reliability and validity as a diagnostic instrument for depression , consistent with the criteria for major depressive disorder as outlined in the Diagnostic and Statistical Manual of Mental Disorders, Fourth Edition (DSMIV). Each of the nine items is scored from 0 to 3, based on the participant’s response: “not at all,” “several days,” “more than half the days,” or “nearly every day.” The total PHQ-9 score ranges from 0 to 27, with scores of 0–4 indicating no depression, and scores ≥5 suggesting the presence of depressive symptoms. Depression severity was further classified as follows: mild (5–9), moderate (10–14), and severe (≥15).

12.Hypertension:

The diagnosis is as follows: Satisfying any one of the following conditions is sufficient:(1). Previously informed of having hypertension. (2). Using antihypertensive drugs. (3). Average systolic pressure ≥ 140 mmHg or average diastolic and systolic pressure ≥ 90 mmHg.

The calculation method for average blood pressure:(1) The diastolic reading with zero is not used to calculate the diastolic average. (2) If all diastolic readings are zero, then the average would be zero.(3) If only one blood pressure reading is obtained, that reading is the average.(4) If there are more than one blood pressure reading, the first reading is always excluded from the average.

13.Hyperlipidemia

was defined by self-reported physician diagnosis, antihypertensive medication use, mean SBP ≥140 mmHg, or mean DBP ≥90 mmHg.

14. Sleep quality

Sleep quality was assessed using a derived overall sleep quality index (OSQI) constructed from the NHANES Sleep Disorders Questionnaire. The index incorporated six self-reported sleep-related symptoms during the preceding month: difficulty falling asleep, waking during the night with difficulty returning to sleep, waking too early and being unable to return to sleep, feeling unrested during the day, excessive daytime sleepiness, and feeling that insufficient sleep was obtained. These items correspond to the NHANES variables SLQ080–SLQ130.

For each item, responses were coded according to the original NHANES frequency categories: never (0), rarely—approximately once per month (1), sometimes—2–4 times per month (2), often—5–15 times per month (3), and almost always—16–30 times per month (4). Scores were summed to obtain an overall sleep-quality score ranging from 0 to 24, with higher values indicating poorer sleep quality.

15. Physical activity

Physical activity information was obtained from the NHANES Physical Activity Questionnaire (PAQ). The questionnaire assesses vigorous- and moderate-intensity activity performed in occupational and recreational settings and walking or bicycling for transportation, with information on frequency and duration. Activity volume was quantified using the NHANES-recommended MET values, including 8 METs for vigorous activity and 4 METs for moderate activity; transportation-related walking or bicycling was assigned 4 METs in the relevant NHANES questionnaire cycles.

16. Frailty index

Frailty was assessed using the commonly applied 49-item deficit-accumulation Frailty Index (FI) developed for NHANES. The 49 deficits span seven broad domains, including cognitive function, functional dependence, depressive symptoms, chronic conditions, healthcare utilization, physical/anthropometric measures, and laboratory abnormalities. Individual deficits were coded from 0 to 1 according to deficit severity.

The FI was calculated as:

Participants were required to have valid information for at least 80% of the 49 items (≥40 items). FI values range from 0 to 1, with higher scores indicating greater frailty.

Supplementary Figures S2–S7: Cohort-Specific Model Validation and Diagnostics

**General Note for Figures S2–S7:** Supplementary Figures S2 to S7 present the comprehensive model validation and diagnostic plots for the multivariable regression analyses across the six independent study cohorts. These diagnostics typically encompass the evaluation of multicollinearity (e.g., Variance Inflation Factors [VIF]), residual distributions, and goodness-of-fit assessments. Across all datasets, the diagnostic results consistently confirmed the absence of severe multicollinearity, validated the fundamental statistical assumptions, and demonstrated acceptable model calibration, thereby rigorously supporting the robustness and reliability of our primary epidemiological findings.

Supplementary Figure S2**.** Model validation and diagnostic plots for the **CHARLS longitudinal** cohort.

Supplementary Figure S3. Model validation and diagnostic plots for the ELSA cohort.

 Supplementary Figure S4. Model validation and diagnostic plots for the CHARLS cross-sectional cohort.

Supplementary Figure S5. Model validation and diagnostic plots for the NHANES cohort.

 Supplementary Figure S6. Model validation and diagnostic plots for the Ganzhou cohort.

Supplementary Figure S7. Model validation and diagnostic plots for the Zhaotong cohort.

Supplementary Table S1. Baseline characteristics of participants in the CHARLS longitudinal cohort, stratified by TyG index quartiles.

| Variables | Total  (*N* = 5927) | Q1 (*N* = 1482)  <8.3 | Q2 (*N* = 1481)  8.3-8.6 | Q3 (*N* = 1482)  8.6-9.1 | Q4 (*N* = 1482)  >9.1 | *P* value |
| --- | --- | --- | --- | --- | --- | --- |
| **Demographic** |  |  |  |  |  |  |
| Age, y | 58.8 ± 8.5 | 58.7 ± 8.8 | 59.1 ± 8.5 | 59.0 ± 8.5 | 58.3 ± 8.3 | 0.034 |
| Sex, n (%) |  |  |  |  |  | < 0.001 |
| Female | 3195 (53.9) | 676 (45.6) | 802 (54.2) | 863 (58.2) | 854 (57.6) |  |
| Male | 2732 (46.1) | 806 (54.4) | 679 (45.8) | 619 (41.8) | 628 (42.4) |  |
| Marital status, n (%) |  |  |  |  |  | 0.319 |
| No | 634 (10.7) | 155 (10.5) | 160 (10.8) | 175 (11.8) | 144 (9.7) |  |
| Yes | 5293 (89.3) | 1327 (89.5) | 1321 (89.2) | 1307 (88.2) | 1338 (90.3) |  |
| Educational status, n (%) |  |  |  |  |  | 0.707 |
| Illiterate | 2782 (47.0) | 692 (46.7) | 702 (47.4) | 715 (48.3) | 673 (45.5) |  |
| Primary school | 1343 (22.7) | 336 (22.7) | 347 (23.4) | 316 (21.4) | 344 (23.2) |  |
| Junior high school | 1215 (20.5) | 310 (20.9) | 292 (19.7) | 292 (19.7) | 321 (21.7) |  |
| High school or above | 583 (9.8) | 144 (9.7) | 140 (9.5) | 157 (10.6) | 142 (9.6) |  |
| Residence, n (%) |  |  |  |  |  | < 0.001 |
| Urban community | 973 (16.4) | 197 (13.3) | 232 (15.7) | 257 (17.4) | 287 (19.4) |  |
| Rural village | 4952 (83.6) | 1284 (86.7) | 1249 (84.3) | 1224 (82.6) | 1195 (80.6) |  |
| Race, n (%) |  |  |  |  |  | 0.093 |
| Non-Han | 359 (6.6) | 103 (7.5) | 92 (6.8) | 70 (5.2) | 94 (6.9) |  |
| Han | 5088 (93.4) | 1271 (92.5) | 1265 (93.2) | 1280 (94.8) | 1272 (93.1) |  |
| **Lifestyle** |  |  |  |  |  |  |
| Drinking status, n (%) |  |  |  |  |  | < 0.001 |
| Never | 4349 (73.4) | 1015 (68.5) | 1095 (73.9) | 1126 (76) | 1113 (75.3) |  |
| Past | 357 (6.0) | 85 (5.7) | 81 (5.5) | 96 (6.5) | 95 (6.4) |  |
| Current | 1217 (20.5) | 382 (25.8) | 305 (20.6) | 259 (17.5) | 271 (18.3) |  |
| Smoking status, n (%) |  |  |  |  |  | < 0.001 |
| Never | 3651 (61.6) | 841 (56.7) | 919 (62.1) | 936 (63.2) | 955 (64.4) |  |
| Past | 489 (8.3) | 120 (8.1) | 104 (7.0) | 128 (8.6) | 137 (9.2) |  |
| Current | 1787 (30.2) | 521 (35.2) | 458 (30.9) | 418 (28.2) | 390 (26.3) |  |
| **Health Status** |  |  |  |  |  |  |
| Hypertension, n (%) |  |  |  |  |  | < 0.001 |
| No | 3084 (52.2) | 937 (63.4) | 831 (56.2) | 717 (48.5) | 599 (40.6) |  |
| Yes | 2826 (47.8) | 542 (36.6) | 647 (43.8) | 761 (51.5) | 876 (59.4) |  |
| DM, n (%) |  |  |  |  |  | < 0.001 |
| No | 4971 (84.7) | 1409 (95.7) | 1358 (92.3) | 1273 (87.1) | 931 (63.5) |  |
| Yes | 901 (15.3) | 63 (4.3) | 114 (7.7) | 189 (12.9) | 535 (36.5) |  |
| Cancer, n (%) |  |  |  |  |  | 0.150 |
| No | 5842 (99.2) | 1465 (99.2) | 1463 (99.3) | 1466 (99.5) | 1448 (98.7) |  |
| Yes | 50 (0.8) | 12 (0.8) | 11 (0.7) | 8 (0.5) | 19 (1.3) |  |
| CPD, n (%) |  |  |  |  |  | 0.199 |
| No | 5333 (90.4) | 1333 (90.3) | 1318 (89.2) | 1334 (90.3) | 1348 (91.6) |  |
| Yes | 569 (9.6) | 143 (9.7) | 159 (10.8) | 143 (9.7) | 124 (8.4) |  |
| Heart Diseases, n (%) |  |  |  |  |  | < 0.001 |
| No | 5119 (87.0) | 1337 (90.7) | 1321 (89.6) | 1253 (85.3) | 1208 (82.3) |  |
| Yes | 766 (13.0) | 137 (9.3) | 153 (10.4) | 216 (14.7) | 260 (17.7) |  |
| Stroke, n (%) |  |  |  |  |  | 0.077 |
| No | 5762 (97.5) | 1452 (98.2) | 1434 (97.2) | 1446 (97.8) | 1430 (96.9) |  |
| Yes | 145 (2.5) | 26 (1.8) | 41 (2.8) | 32 (2.2) | 46 (3.1) |  |
| Depression, n (%) |  |  |  |  |  | 0.867 |
| No | 5834 (98.8) | 1461 (98.8) | 1451 (98.8) | 1458 (98.8) | 1464 (99.1) |  |
| Yes | 68 (1.2) | 18 (1.2) | 18 (1.2) | 18 (1.2) | 14 (0.9) |  |
| Arthritis, n (%) |  |  |  |  |  | 0.067 |
| No | 3884 (65.7) | 1008 (68.2) | 975 (65.9) | 939 (63.6) | 962 (65.1) |  |
| Yes | 2030 (34.3) | 471 (31.8) | 505 (34.1) | 538 (36.4) | 516 (34.9) |  |
| Dyslipidemia, n (%) |  |  |  |  |  | < 0.001 |
| No | 5125 (88.3) | 1371 (94) | 1333 (91.7) | 1261 (87.6) | 1160 (80.1) |  |
| Yes | 676 (11.7) | 87 (6) | 121 (8.3) | 179 (12.4) | 289 (19.9) |  |
| Liver Diseases, n (%) |  |  |  |  |  | 0.167 |
| No | 5667 (96.4) | 1408 (95.6) | 1429 (97.1) | 1421 (96.6) | 1409 (96.2) |  |
| Yes | 214 (3.6) | 65 (4.4) | 43 (2.9) | 50 (3.4) | 56 (3.8) |  |
| CKD, n (%) |  |  |  |  |  | 0.631 |
| No | 5542 (94.3) | 1386 (94.1) | 1381 (94.2) | 1380 (93.8) | 1395 (94.9) |  |
| Yes | 338 (5.7) | 87 (5.9) | 85 (5.8) | 91 (6.2) | 75 (5.1) |  |
| Chronic gastritis, n (%) |  |  |  |  |  | 0.292 |
| No | 4573 (77.5) | 1136 (76.9) | 1122 (76.1) | 1160 (78.6) | 1155 (78.3) |  |
| Yes | 1330 (22.5) | 342 (23.1) | 353 (23.9) | 315 (21.4) | 320 (21.7) |  |
| Asthma, n (%) |  |  |  |  |  | 0.693 |
| No | 5617 (95.1) | 1401 (95) | 1402 (94.9) | 1400 (94.9) | 1414 (95.7) |  |
| Yes | 290 (4.9) | 74 (5.0) | 76 (5.1) | 76 (5.1) | 64 (4.3) |  |
| **Anthropometric Measures** |  |  |  |  |  |  |
| BMI, kg/m^2^ | 23.5 (21.2, 26.1) | 22.0 (20.2, 24.2) | 23.0 (21.1, 25.3) | 24.0 (21.6, 26.6) | 25.2 (22.8, 27.8) | < 0.001 |
| WBC, ×10⁹/L | 6.3 ± 1.9 | 6.0 ± 1.8 | 6.2 ± 1.8 | 6.4 ± 1.9 | 6.7 ± 1.9 | < 0.001 |
| MCV, fL | 91.1 ± 7.5 | 91.8 ± 7.3 | 91.0 ± 7.9 | 91.1 ± 7.5 | 90.5 ± 7.3 | < 0.001 |
| PLT, ×10⁹/L | 212.8 ± 74.4 | 207.9 ± 68.9 | 210.0 ± 68.3 | 214.6 ± 87.0 | 218.7 ± 71.6 | < 0.001 |
| BUN, mg/dl | 15.6 ± 4.3 | 16.2 ± 4.7 | 15.5 ± 4.2 | 15.3 ± 4.0 | 15.3 ± 4.1 | < 0.001 |
| Creatinine, mg/dl | 0.8 ± 0.2 | 0.8 ± 0.2 | 0.8 ± 0.2 | 0.8 ± 0.2 | 0.8 ± 0.2 | 0.002 |
| Hs-CRP, mg/L | 1.0 (0.6, 2.1) | 0.8 (0.5, 1.7) | 0.9 (0.5, 2.0) | 1.1 (0.6, 2.1) | 1.3 (0.7, 2.5) | < 0.001 |
| HbA1c, % | 5.3 ± 0.8 | 5.1 ± 0.5 | 5.2 ± 0.5 | 5.2 ± 0.6 | 5.7 ± 1.3 | < 0.001 |
| Cystatin C, mg/dl | 1.0 ± 0.2 | 1.0 ± 0.2 | 1.0 ± 0.2 | 1.0 ± 0.2 | 0.9 ± 0.2 | < 0.001 |
| Anemia, n (%) |  |  |  |  |  | 0.002 |
| No | 4879 (82.3) | 1184 (79.9) | 1202 (81.2) | 1234 (83.3) | 1259 (85.0) |  |
| Yes | 1048 (17.7) | 298 (20.1) | 279 (18.8) | 248 (16.7) | 223 (15.0) |  |

Data are presented as mean ± standard deviation (SD) for normally distributed continuous variables, median (interquartile range, IQR) for non-normally distributed continuous variables, and unweighted number (percentage, %) for categorical variables. Statistical differences across the four TyG index quartiles (Q1–Q4) were evaluated using one-way analysis of variance (ANOVA) or the Kruskal-Wallis H test for continuous variables, and Pearson's chi-square test for categorical variables.

CHARLS, China Health and Retirement Longitudinal Study; TyG, triglyceride-glucose; BMI Body mass index, WBC White blood cell count, MCV Mean corpuscular volume, PLT Platelet count, BUN Blood urea nitrogen, HsCRP High-sensitivity C-reactive protein, HbA1c Glycated hemoglobin, DM Diabetes mellitus, CPD Chronic pulmonary disease, CKD Chronic kidney disease, SD Standard deviation, IQR Interquartile range.

Supplementary Table S2. Baseline characteristics of participants in the CHARLS cross-sectional cohort according to TyG index quartiles.

| Variables | Total  (*N* = 10732) | Q1 (*N* = 2683)  <8.2 | Q2 (*N* = 2682)  8.2-8.6 | Q3 (*N* = 2684)  8.6-9.1 | Q4 (*N* = 2683)  >9.1 | *P* value |
| --- | --- | --- | --- | --- | --- | --- |
| **Demographic** |  |  |  |  |  |  |
| Age, y | 59.5 ± 9.4 | 59.6 ± 9.8 | 59.5 ± 9.5 | 59.7 ± 9.3 | 59.0 ± 9.1 | 0.045 |
| Sex, n (%) |  |  |  |  |  | < 0.001 |
| Female | 5764 (53.7) | 1212 (45.2) | 1438 (53.6) | 1566 (58.3) | 1548 (57.7) |  |
| Male | 4968 (46.3) | 1471 (54.8) | 1244 (46.4) | 1118 (41.7) | 1135 (42.3) |  |
| Marital status, n (%) |  |  |  |  |  | 0.151 |
| No | 1299 (12.1) | 337 (12.6) | 317 (11.8) | 348 (13.0) | 297 (11.1) |  |
| Yes | 9433 (87.9) | 2346 (87.4) | 2365 (88.2) | 2336 (87) | 2386 (88.9) |  |
| Educational status, n (%) |  |  |  |  |  | 0.010 |
| Illiterate | 5164 (48.2) | 1329 (49.6) | 1263 (47.1) | 1337 (49.9) | 1235 (46.1) |  |
| Primary school | 2297 (21.4) | 591 (22.0) | 602 (22.5) | 544 (20.3) | 560 (20.9) |  |
| Junior high school | 2123 (19.8) | 514 (19.2) | 525 (19.6) | 514 (19.2) | 570 (21.3) |  |
| High school or above | 1138 (10.6) | 248 (9.2) | 290 (10.8) | 285 (10.6) | 315 (11.8) |  |
| Race, n (%) |  |  |  |  |  | 0.008 |
| Non-Han | 637 (7.2) | 180 (8.2) | 176 (8.0) | 128 (5.8) | 153 (7.0) |  |
| Han | 8182 (92.8) | 2025 (91.8) | 2035 (92) | 2093 (94.2) | 2029 (93) |  |
| Residence, n (%) |  |  |  |  |  | < 0.001 |
| Urban community | 1966 (18.3) | 386 (14.4) | 442 (16.5) | 509 (19.0) | 629 (23.5) |  |
| Rural village | 8762 (81.7) | 2296 (85.6) | 2240 (83.5) | 2173 (81.0) | 2053 (76.5) |  |
| **Lifestyle** |  |  |  |  |  |  |
| Smoking status, n (%) |  |  |  |  |  | < 0.001 |
| Never | 6605 (61.5) | 1504 (56.1) | 1662 (62.0) | 1701 (63.4) | 1738 (64.8) |  |
| Past | 948 (8.8) | 245 (9.1) | 205 (7.6) | 243 (9.1) | 255 (9.5) |  |
| Current | 3179 (29.6) | 934 (34.8) | 815 (30.4) | 740 (27.6) | 690 (25.7) |  |
| Drinking status, n (%) |  |  |  |  |  | < 0.001 |
| Never | 7891 (73.6) | 1837 (68.5) | 1993 (74.3) | 2035 (75.8) | 2026 (75.6) |  |
| Past | 663 (6.2) | 172 (6.4) | 147 (5.5) | 171 (6.4) | 173 (6.5) |  |
| Current | 2172 (20.2) | 673 (25.1) | 541 (20.2) | 477 (17.8) | 481 (17.9) |  |
| **Health Status** |  |  |  |  |  |  |
| Hypertension, n (%) |  |  |  |  |  | < 0.001 |
| No | 5696 (53.3) | 1677 (62.7) | 1556 (58.1) | 1313 (49.2) | 1150 (43.0) |  |
| Yes | 4999 (46.7) | 996 (37.3) | 1122 (41.9) | 1356 (50.8) | 1525 (57.0) |  |
| DM, n (%) |  |  |  |  |  | < 0.001 |
| No | 8977 (84.4) | 2547 (95.8) | 2455 (92.1) | 2285 (86.1) | 1690 (63.6) |  |
| Yes | 1658 (15.6) | 111 (4.2) | 210 (7.9) | 369 (13.9) | 968 (36.4) |  |
| Cancer, n (%) |  |  |  |  |  | 0.105 |
| No | 10574 (99.1) | 2649 (99.3) | 2650 (99.1) | 2648 (99.2) | 2627 (98.7) |  |
| Yes | 99 (0.9) | 19 (0.7) | 23 (0.9) | 22 (0.8) | 35 (1.3) |  |
| CPD, n (%) |  |  |  |  |  | 0.063 |
| No | 9620 (90.0) | 2395 (89.6) | 2388 (89.3) | 2402 (90.0) | 2435 (91.3) |  |
| Yes | 1064 (10.0) | 279 (10.4) | 286 (10.7) | 268 (10) | 231 (8.7) |  |
| Heart Diseases, n (%) |  |  |  |  |  | < 0.001 |
| No | 9333 (87.5) | 2420 (90.7) | 2381 (89.0) | 2327 (87.3) | 2205 (82.8) |  |
| Yes | 1335 (12.5) | 248 (9.3) | 293 (11.0) | 337 (12.7) | 457 (17.2) |  |
| Stroke, n (%) |  |  |  |  |  | 0.005 |
| No | 10405 (97.3) | 2619 (98.0) | 2602 (97.3) | 2609 (97.4) | 2575 (96.4) |  |
| Yes | 292 (2.7) | 54 (2) | 73 (2.7) | 69 (2.6) | 96 (3.6) |  |
| Depression, n (%) |  |  |  |  |  | 0.707 |
| No | 10535 (98.6) | 2638 (98.6) | 2623 (98.5) | 2632 (98.5) | 2642 (98.8) |  |
| Yes | 148 (1.4) | 38 (1.4) | 40 (1.5) | 39 (1.5) | 31 (1.2) |  |
| Arthritis, n (%) |  |  |  |  |  | 0.010 |
| No | 6970 (65.1) | 1792 (67) | 1762 (65.8) | 1678 (62.7) | 1738 (65.0) |  |
| Yes | 3733 (34.9) | 884 (33) | 916 (34.2) | 997 (37.3) | 936 (35.0) |  |
| Dyslipidemia, n (%) |  |  |  |  |  | < 0.001 |
| No | 9414 (89.6) | 2503 (95) | 2444 (92.5) | 2324 (89.2) | 2143 (81.6) |  |
| Yes | 1095 (10.4) | 133 (5) | 199 (7.5) | 281 (10.8) | 482 (18.4) |  |
| Liver Diseases, n (%) |  |  |  |  |  | 0.083 |
| No | 10263 (96.4) | 2554 (95.8) | 2587 (97) | 2571 (96.6) | 2551 (96.2) |  |
| Yes | 385 (3.6) | 113 (4.2) | 80 (3) | 90 (3.4) | 102 (3.8) |  |
| CKD, n (%) |  |  |  |  |  | 0.741 |
| No | 10006 (93.9) | 2502 (93.8) | 2491 (93.5) | 2505 (94) | 2508 (94.2) |  |
| Yes | 652 (6.1) | 165 (6.2) | 173 (6.5) | 160 (6.0) | 154 (5.8) |  |
| Chronic gastritis, n (%) |  |  |  |  |  | 0.010 |
| No | 8275 (77.4) | 2045 (76.4) | 2024 (75.7) | 2109 (78.9) | 2097 (78.5) |  |
| Yes | 2420 (22.6) | 631 (23.6) | 651 (24.3) | 563 (21.1) | 575 (21.5) |  |
| Asthma, n (%) |  |  |  |  |  | 0.742 |
| No | 10197 (95.4) | 2552 (95.5) | 2543 (95.2) | 2548 (95.2) | 2554 (95.7) |  |
| Yes | 491 (4.6) | 120 (4.5) | 129 (4.8) | 128 (4.8) | 114 (4.3) |  |
| **Anthropometric Measures** |  |  |  |  |  |  |
| BMI, kg/m^2^ | 23.1 (20.8, 25.8) | 21.7 (19.9, 23.9) | 22.6 (20.6, 25.0) | 23.6 (21.2, 26.3) | 24.8 (22.5, 27.5) | < 0.001 |
| WBC, ×10⁹ cells/L | 6.2 ± 2.1 | 5.9 ± 1.8 | 6.1 ± 1.8 | 6.3 ± 2.8 | 6.6 ± 1.9 | < 0.001 |
| MCV, fL | 90.6 ± 8.6 | 90.8 ± 9.1 | 90.3 ± 8.9 | 90.7 ± 8.3 | 90.4 ± 7.9 | 0.091 |
| PLT, ×10⁹ cel/L | 211.7 ± 74.9 | 208.4 ± 72.8 | 208.3 ± 72.6 | 213.8 ± 81.0 | 216.3 ± 72.5 | < 0.001 |
| BUN, mg/dl | 15.7 ± 4.6 | 16.3 ± 5.0 | 15.8 ± 4.7 | 15.4 ± 4.3 | 15.5 ± 4.4 | < 0.001 |
| Creatinine, mg/dl | 0.8 ± 0.2 | 0.8 ± 0.2 | 0.8 ± 0.3 | 0.8 ± 0.2 | 0.8 ± 0.2 | 0.008 |
| Hs-CRP, mg/L | 1.0 (0.6, 2.2) | 0.8 (0.5, 1.9) | 0.9 (0.5, 2.0) | 1.1 (0.6, 2.2) | 1.3 (0.7, 2.7) | < 0.001 |
| HbA1c, % | 5.3 ± 0.8 | 5.1 ± 0.5 | 5.1 ± 0.5 | 5.2 ± 0.6 | 5.7 ± 1.3 | < 0.001 |
| Anemia, n (%) |  |  |  |  |  | < 0.001 |
| No | 9009 (83.9) | 2093 (78) | 2223 (82.9) | 2311 (86.1) | 2382 (88.8) |  |
| Yes | 1723 (16.1) | 590 (22) | 459 (17.1) | 373 (13.9) | 301 (11.2) |  |

Data are expressed as mean ± standard deviation (SD), median (interquartile range, IQR), or frequency (percentage, %), as appropriate. Comparisons among the TyG index quartiles were performed using one-way analysis of variance (ANOVA) or the Kruskal-Wallis H test for continuous variables, and Pearson's chi-square test for categorical parameters.

CHARLS, China Health and Retirement Longitudinal Study; TyG, triglyceride-glucose; BMI Body mass index, WBC White blood cell count, MCV Mean corpuscular volume, PLT Platelet count, BUN Blood urea nitrogen, HsCRP High-sensitivity C-reactive protein, HbA1c Glycated hemoglobin, DM Diabetes mellitus, CPD Chronic pulmonary disease, CKD Chronic kidney disease, SD Standard deviation, IQR Interquartile range.

Supplementary Table S3. Baseline characteristics of participants in the ELSA cohort according to TyG index quartiles.

| Variables | Total  (*N* = 2459) | Q1 (*N* = 612)  <8.2 | Q2 (*N* = 612)  8.2-8.5 | Q3 (*N* = 613)  8.5-8.9 | Q4 (*N* = 622)  >8.9 | *P* value |
| --- | --- | --- | --- | --- | --- | --- |
| **Demographic** |  |  |  |  |  |  |
| Age, y | 63.1 ± 7.0 | 62.3 ± 7.0 | 63.6 ± 7.0 | 63.5 ± 6.9 | 63.1 ± 6.9 | 0.005 |
| Sex, n (%) |  |  |  |  |  | < 0.001 |
| Female | 1367 (55.6) | 375 (61.3) | 367 (60.0) | 329 (53.7) | 296 (47.6) |  |
| Male | 1092 (44.4) | 237 (38.7) | 245 (40.0) | 284 (46.3) | 326 (52.4) |  |
| Marital status, n (%) |  |  |  |  |  | < 0.001 |
| No | 707(28.7) | 435 (71.1) | 441 (71.1) | 424 (69.2) | 452 (72.7) |  |
| Yes | 1752 (71.3） | 177 (28.9) | 171 (27.9) | 189 (30.8) | 170 (27.3) |  |
| Educational status, n (%) |  |  |  |  |  | < 0.001 |
| Illiterate | 668 (29.4) | 142 (24.8) | 155 (27.5) | 161 (28.7) | 210 (36.7) |  |
| Primary school | 511 (22.5) | 127 (22.2) | 133 (23.6) | 123 (21.9) | 128 (22.4) |  |
| Junior high school | 602 (26.5) | 154 (26.9) | 150 (26.6) | 168 (29.9) | 130 (22.7) |  |
| High school or above | 489 (21.5) | 150 (26.2) | 126 (22.3) | 109 (19.4) | 104 (18.2) |  |
| Race, n (%) |  |  |  |  |  | 0.926 |
| Non-White | 37 (1.5) | 9 (1.5) | 9 (1.5) | 8 (1.3) | 11 (1.8) |  |
| White | 2422 (98.5) | 603 (98.5) | 603 (98.5) | 605 (98.7) | 611 (98.2) |  |
| **Lifestyle** |  |  |  |  |  |  |
| Smoking status, n (%) |  |  |  |  |  | 0.023 |
| Never | 1093 (44.4) | 302 (49.3) | 279 (45.6) | 257 (41.9) | 255 (41) |  |
| Past | 1066 (43.4) | 241 (39.4) | 272 (44.4) | 275 (44.9) | 278 (44.7) |  |
| Current | 300 (12.2) | 69 (11.3) | 61 (10) | 81 (13.2) | 89 (14.3) |  |
| Drinking status, n (%) |  |  |  |  |  | 0.492 |
| Never | 169 (7.3) | 36 (6.3) | 40 (7) | 43 (7.4) | 50 (8.6) |  |
| Ever | 2136 (92.7) | 538 (93.7) | 531 (93) | 535 (92.6) | 532 (91.4) |  |
| **Health Status** |  |  |  |  |  |  |
| Hypertension, n (%) |  |  |  |  |  | < 0.001 |
| No | 1667 (67.8) | 454 (74.2) | 423 (69.1) | 418 (68.2) | 372 (59.8) |  |
| Yes | 792 (32.2) | 158 (25.8) | 189 (30.9) | 195 (31.8) | 250 (40.2) |  |
| DM, n (%) |  |  |  |  |  | < 0.001 |
| No | 2397 (97.5) | 607 (99.2) | 599 (97.9) | 600 (97.9) | 591 (95) |  |
| Yes | 62 (2.5) | 5 (0.8) | 13 (2.1) | 13 (2.1) | 31 (5) |  |
| Cancer, n (%) |  |  |  |  |  | 0.397 |
| No | 2308 (93.9) | 578 (94.4) | 578 (94.4) | 577 (94.1) | 575 (92.4) |  |
| Yes | 151 (6.1) | 34 (5.6) | 34 (5.6) | 36 (5.9) | 47 (7.6) |  |
| CPD, n (%) |  |  |  |  |  | 0.088 |
| No | 2382 (96.9) | 584 (95.4) | 595 (97.2) | 600 (97.9) | 603 (96.9) |  |
| Yes | 77 (3.1) | 28 (4.6) | 17 (2.8) | 13 (2.1) | 19 (3.1) |  |
| Heart Diseases, n (%) |  |  |  |  |  | 0.185 |
| No | 2181 (88.8) | 546 (89.2) | 533 (87.2) | 556 (90.8) | 546 (87.8) |  |
| Yes | 276 (11.2) | 66 (10.8) | 78 (12.8) | 56 (9.2) | 76 (12.2) |  |
| Stroke, n (%) |  |  |  |  |  | 0.388 |
| No | 2416 (98.3) | 605 (98.9) | 603 (98.5) | 599 (97.7) | 609 (97.9) |  |
| Yes | 43 (1.7) | 7 (1.1) | 9 (1.5) | 14 (2.3) | 13 (2.1) |  |
| Arthritis, n (%) |  |  |  |  |  | 0.389 |
| No | 1696 (69.0) | 439 (71.7) | 418 (68.3) | 414 (67.5) | 425 (68.3) |  |
| Yes | 763 (31.0) | 173 (28.3) | 194 (31.7) | 199 (32.5) | 197 (31.7) |  |
| Antihypertensives, n (%) |  |  |  |  |  | < 0.001 |
| No | 1848 (75.2) | 494 (80.7) | 467 (76.3) | 450 (73.4) | 437 (70.3) |  |
| Yes | 611 (24.8) | 118 (19.3) | 145 (23.7) | 163 (26.6) | 185 (29.7) |  |
| Antidiabetics, n (%) |  |  |  |  |  | < 0.001 |
| No | 2409 (98.0) | 608 (99.3) | 600 (98) | 606 (98.9) | 595 (95.7) |  |
| Yes | 50 (2.0) | 4 (0.7) | 12 (2) | 7 (1.1) | 27 (4.3) |  |
| Respiratory drugs, n (%) |  |  |  |  |  | 0.015 |
| No | 2427 (98.8) | 596 (97.5) | 607 (99.2) | 608 (99.3) | 616 (99) |  |
| Yes | 30 (1.2) | 15 (2.5) | 5 (0.8) | 4 (0.7) | 6 (1) |  |
| Antithrombotic, n (%) |  |  |  |  |  | 0.015 |
| No | 2319 (94.4) | 591 (96.6) | 579 (94.8) | 574 (93.8) | 575 (92.4) |  |
| Yes | 138 (5.6) | 21 (3.4) | 32 (5.2) | 38 (6.2) | 47 (7.6) |  |
| Lipid-lowering drugs, n (%) |  |  |  |  |  | < 0.001 |
| No | 1995 (81.2) | 543 (88.7) | 490 (80.3) | 485 (79.2) | 477 (76.7) |  |
| Yes | 461 (18.8) | 69 (11.3) | 120 (19.7) | 127 (20.8) | 145 (23.3) |  |
| **Anthropometric Measures** |  |  |  |  |  |  |
| BMI, kg/m^2^ | 27.9 ± 4.9 | 25.9 ± 4.0 | 26.9 ± 4.4 | 28.7 ± 4.9 | 30.0 ± 5.2 | < 0.001 |
| WBC, ×10⁹/L | 6.1 ± 1.9 | 5.7 ± 1.7 | 6.0 ± 1.8 | 6.3 ± 1.8 | 6.5 ± 2.1 | < 0.001 |
| Hs-CRP, mg/L | 1.7 (0.8, 3.5) | 1.1 (0.6, 2.3) | 1.6 (0.8, 3.2) | 1.9 (1.0, 3.8) | 2.4 (1.3, 5.0) | < 0.001 |
| HbA1c, % | 5.8 ± 0.5 | 5.6 ± 0.3 | 5.7 ± 0.3 | 5.8 ± 0.4 | 5.9 ± 0.7 | < 0.001 |

Continuous variables are presented as mean ± standard deviation (SD) or median (interquartile range, IQR) based on their distribution, and categorical variables are presented as count (percentage, %). Statistical significance across quartiles was assessed using one-way ANOVA or the Kruskal-Wallis H test for continuous variables, and Pearson's chi-square test for categorical variables.

ELSA, English Longitudinal Study of Ageing; TyG, triglyceride-glucose; DM Diabetes mellitus, CPD Chronic pulmonary disease, BMI Body mass index, WBC White blood cell count, Hs-CRP High-sensitivity C-reactive protein, HbA1c Glycated hemoglobin (Glycosylated hemoglobin), SD Standard deviation, IQR Interquartile range.

Supplementary Table S4. Baseline characteristics of participants in the NHANES cross-sectional cohort according to TyG index quartiles.

| **Characteristic** | **Total** | **Q1 (≤8.32)** | **Q2(8.32-8.72)** | **Q3(8.72-9.15)** | **Q4(≥9.15)** | ***P*-value ^a^** |
| --- | --- | --- | --- | --- | --- | --- |
| Weighted Population. *N* | 94,477,535 | 24,294,548 | 23,829,832 | 24,066,655 | 22,286,500 |  |
| Unweighted Population. *N* | 14559 | 3639 | 3640 | 3640 | 3640 |  |
| **Demographic** | | | | | | |
| Age, , y | 60.19 (0.14) | 59.26 (0.27) | 60.34 (0.27) | 60.68 (0.23) | 60.52 (0.24) | <0.001 |
| Sex, n (%) |  |  |  |  |  | <0.001 |
| Male | 7,187 (47.05%) | 1,661 (41.16%) | 1,782 (46.45%) | 1,799 (48.10%) | 1,945 (52.97%) |  |
| Female | 7,372 (52.95%) | 1,978 (58.84%) | 1,858 (53.55%) | 1,841 (51.90%) | 1,695 (47.03%) |  |
| Race, n (%) |  |  |  |  |  | <0.001 |
| Non-Hispanic White | 6,979 (74.66%) | 1,624 (72.51%) | 1,746 (74.33%) | 1,838 (76.23%) | 1,771 (75.66%) |  |
| Non-Hispanic Black | 2,901 (9.75%) | 1,161 (15.59%) | 780 (10.55%) | 524 (6.75%) | 436 (5.77%) |  |
| Mexican American | 2,211 (5.17%) | 294 (3.13%) | 492 (4.75%) | 620 (5.59%) | 805 (7.39%) |  |
| Other Hispanic | 1,266 (4.44%) | 247 (3.39%) | 320 (4.35%) | 352 (5.04%) | 347 (5.04%) |  |
| Other Race ^b^ | 1,202 (5.98%) | 313 (5.39%) | 302 (6.01%) | 306 (6.39%) | 281 (6.14%) |  |
| Marital status ^c^, n (%) |  |  |  |  |  | 0.520 |
| No | 5,471 (31.58%) | 1,438 (30.98%) | 1,401 (32.96%) | 1,285 (30.95%) | 1,347 (31.46%) |  |
| Yes | 8,971 (68.42%) | 2,180 (69.02%) | 2,214 (67.04%) | 2,319 (69.05%) | 2,258 (68.54%) |  |
| Education level, n (%) |  |  |  |  |  | <0.001 |
| Less than 9th grade | 2,239 (7.56%) | 362 (5.10%) | 524 (7.04%) | 621 (8.56%) | 732 (9.73%) |  |
| 9-11th Grade | 2,101 (10.90%) | 489 (9.61%) | 473 (9.76%) | 548 (11.48%) | 591 (12.91%) |  |
| High school or equivalent | 3,366 (24.67%) | 797 (21.03%) | 862 (24.16%) | 825 (25.35%) | 882 (28.43%) |  |
| Some College or AA degree | 3,779 (28.90%) | 994 (27.64%) | 989 (30.46%) | 899 (27.89%) | 897 (29.70%) |  |
| College graduate or above | 3,051 (27.97%) | 992 (36.62%) | 791 (28.58%) | 738 (26.73%) | 530 (19.23%) |  |
| PIR ^d^,n(%) |  |  |  |  |  | <0.001 |
| ≦1.30 | 3,635 (17.26%) | 773 (14.56%) | 899 (17.08%) | 906 (16.94%) | 1,057 (20.73%) |  |
| 1.30-3.50 | 5,101 (35.46%) | 1,237 (31.94%) | 1,261 (34.17%) | 1,312 (37.76%) | 1,291 (38.12%) |  |
| >3.50 | 4,344 (47.28%) | 1,252 (53.49%) | 1,123 (48.75%) | 1,050 (45.30%) | 919 (41.15%) |  |
| **Lifestyle** | | | | | | |
| Smoking status, n (%) |  |  |  |  |  | <0.001 |
| Never ^e^ | 7,143 (48.96%) | 1,922 (53.11%) | 1,844 (51.06%) | 1,762 (48.07%) | 1,615 (43.16%) |  |
| Former ^f^ | 4,816 (33.42%) | 1,116 (31.28%) | 1,141 (32.67%) | 1,222 (32.73%) | 1,337 (37.28%) |  |
| Now ^g^ | 2,600 (17.62%) | 601 (15.61%) | 655 (16.27%) | 656 (19.19%) | 688 (19.56%) |  |
| Alcohol intake, n (%) |  |  |  |  |  | <0.001 |
| Never ^h^ | 1,985 (12.22%) | 458 (11.06%) | 469 (11.75%) | 528 (12.72%) | 530 (13.43%) |  |
| Former ^i^ | 2,909 (19.81%) | 602 (17.34%) | 698 (18.29%) | 761 (20.30%) | 848 (23.61%) |  |
| Current ^j^ | 8,074 (67.97%) | 2,137 (71.60%) | 2,065 (69.96%) | 2,009 (66.98%) | 1,863 (62.96%) |  |
| **Health Status** | | | | | | |
| Dyslipidemia, n (%) | 11,906 (82.78%) | 2,278 (63.42%) | 2,806 (79.37%) | 3,252 (91.02%) | 3,570 (98.62%) | <0.001 |
| Hypertension, n (%) | 8,711 (53.83%) | 1,925 (43.51%) | 2,071 (50.86%) | 2,260 (56.04%) | 2,455 (65.89%) | <0.001 |
| DM ^k^, n (%) | 4,220 (22.22%) | 455 (8.51%) | 671 (13.19%) | 1,091 (23.21%) | 2,003 (45.77%) | <0.001 |
| CKD ^L^, n (%) | 3,873 (20.65%) | 771 (16.47%) | 849 (18.24%) | 975 (20.47%) | 1,278 (27.99%) | <0.001 |
| COPD ^m^, n (%) | 825 (7.16%) | 194 (6.80%) | 215 (7.44%) | 201 (7.45%) | 215 (6.94%) | 0.840 |
| Cancer, n (%) | 2,093 (15.06%) | 522 (14.59%) | 500 (14.76%) | 542 (15.31%) | 529 (15.61%) | 0.800 |
| Depression, n (%) | 2,503 (22.41%) | 603 (18.77%) | 569 (19.53%) | 636 (23.77%) | 695 (28.91%) | <0.001 |
| Heart disease ^n^, n (%) | 1,717 (10.06%) | 333 (7.40%) | 373 (8.51%) | 453 (10.96%) | 558 (13.66%) | <0.001 |
| Asthma, n (%) | 1,848 (13.19%) | 479 (12.96%) | 434 (12.51%) | 459 (13.02%) | 476 (14.35%) | 0.430 |
| Arthritis, n (%) | 6,033 (40.40%) | 1,396 (36.46%) | 1,469 (39.34%) | 1,562 (41.20%) | 1,606 (44.96%) | <0.001 |
| **Anthropometric Measures** | | | | | | |
| WBC, ×10⁹ /L | 6.40 (5.40, 7.70) | 5.70 (4.86, 7.00) | 6.20 (5.30, 7.50) | 6.60 (5.60, 7.96) | 7.10 (6.00, 8.40) | <0.001 |
| RDW, | 12.90 (12.40, 13.60) | 13.10 (12.50, 13.70) | 13.00 (12.50, 13.60) | 12.90 (12.40, 13.50) | 12.90 (12.40, 13.60) | <0.001 |
| PLT, ×10⁹ /L | 246.08 (0.98) | 236.07 (1.63) | 246.92 (1.66) | 251.05 (1.78) | 250.73 (1.51) | <0.001 |
| Creatinine, μmol/L | 76.02 (63.65, 88.40) | 74.26 (61.90, 88.40) | 76.91 (63.65, 88.40) | 76.91 (63.65, 88.40) | 79.56 (64.53, 90.17) | <0.001 |
| BUN, mmol/L | 5.00 (3.93, 6.07) | 5.00 (3.93, 6.07) | 5.00 (3.93, 6.07) | 5.00 (3.93, 6.07) | 5.00 (4.28, 6.43) | <0.001 |
| MCV, fL | 90.64 (0.09) | 91.05 (0.16) | 90.67 (0.14) | 90.64 (0.13) | 90.15 (0.11) | <0.001 |
| BMI, kg/m^2^ | 28.10 (24.67, 32.33) | 25.77 (22.80, 29.42) | 27.40 (24.23, 31.21) | 29.20 (25.75, 33.23) | 30.25 (27.13, 34.67) | <0.001 |
| **Dietary Intake** | | | | | | |
| VB12, mg | 354.98  (264.20, 485.60) | 384.50  (280.36, 529.01) | 355.50  (267.63, 481.19) | 350.87  (260.51, 472.32) | 338.74  (251.66, 456.82) | <0.001 |
| Iron, mg | 12.92 (9.17, 18.13) | 12.86 (9.01, 18.37) | 13.04 (9.21, 18.18) | 12.64 (9.15, 17.37) | 13.26 (9.32, 18.67) | 0.047 |
| Total folate, mcg | 343.00  (235.00, 487.00) | 351.00  (245.00, 508.00) | 343.00  (235.00, 483.99) | 335.00  (230.00, 479.00) | 342.00  (233.90, 479.38) | 0.062 |
| Anemia, n (%) | 1,716 (8.01%) | 601 (11.05%) | 447 (8.51%) | 338 (6.60%) | 330 (5.70%) | <0.001 |

Continuous variables are presented as survey-weighted mean (SE) or median (IQR), as appropriate. Counts are unweighted, whereas percentages are survey weighted

^a^ Wilcoxon rank-sum test for complex survey samples; chi-squared test with Rao & Scott's second-order correction.

^b^ Including Multi-Racial

^c^ NO: Never married/Other: widowed, divorced, or separated individuals, YES: Married/ Living with partner

^d^ Categorized into the following 3 levels based on the family poverty income ratio: <1.3, ≥1.3 to 3.5, and >3.5

^e^ Defined as individuals who have smoked fewer than 100 cigarettes in their lifetime

^f^ Defined as individuals who have smoked more than 100 cigarettes in their lifetime and do not smoke at all now

^g^Defined as individuals who have smoked more than 100 cigarettes in their lifetime and smoke some days or every day

^h^ Defined as individuals who have had fewer than 12 drinks in their lifetime.

^i^ Defined as individuals who have had 12 or more drinks in a year but did not drink in the past year, or who did not drink in the past year but have had 12 or more drinks at some point in their lifetime.

^j^ Current drinking status includes: light drinking (≤1 drink per day for women, ≤2 drinks per day for men); moderate drinking (≥2 drinks per day for women, ≥3 drinks per day for men, or binge drinking ≥2 days per month); heavy drinking (≥3 drinks per day for women, ≥4 drinks per day for men, or binge drinking on ≥5 days per month [≥4 drinks per occasion for women, ≥5 drinks per occasion for men]).

^k^ Defined as: Diabetes, Diabetes is diagnosed as follows. One of the following conditions must be met: doctor told you have diabetes, glycohemoglobin HbA1c (%) >= 6.5, fasting glucose (mmol/l) >= 7.0, random blood glucose (mmol/l) >= 11.1, two-hour OGTT blood glucose (mmol/l) >= 11.1, Use of diabetes medication or insulin

^L^ Defined as: Chronic Kidney Disease (CKD) cases were defined as individuals with an eGFR < 60 mL/(min*1.73 m2) and eGFR was calculated using the CKD Epidemiology Collaboration (CKD-EPI) creatinine equation. The enrolled participants were divided into non-CKD group with eGFR ≥ 60 mL/(min*1.73 m2) and CKD group with eGFR < 60 mL/(min*1.73 m^2^).

^m^ The diagnosis of Chronic Obstructive Pulmonary Disease(COPD), was established based on one or more of the following criteria: (1) a post-bronchodilator FEV1/FVC ratio < 0.70; (2) a diagnosis of emphysema confirmed by a physician or other healthcare professional; or (3) for individuals aged 40 years or older, a history of smoking combined with chronic bronchitis and treatment with selective phosphodiesterase-4 (PDE-4) inhibitors, mast cell stabilizers, leukotriene modifiers, or inhaled corticosteroids.

^n^ Heart disease: self-reported previous diagnosis of coronary heart disease, angina, heart attack, or congestive heart failure, with any one of these conditions being sufficient for diagnosis.

NHANES, National Health and Nutrition Examination Survey, WBC: white blood cell, RDW: red blood cell distribution width, PLT: blood platelet, Cr: creatinine, BUN: blood urea nitrogen, MCV: Mean Corpuscular Volume, BMI: Body Mass Index, VB12: vitamin B12, TyG: triglyceride glucose index, SE: Standard Error

Supplementary Table S5. Baseline characteristics of participants in the Ganzhou clinical registry cohort according to TyG index quartiles.

| Variables | Total  (*N* = 32395) | Q1(*N*= 8097)  <8.25 | Q2(*N*= 8096)  8.25-8.62 | Q3(*N*= 8102)  8.62-9.02 | Q4(*N* = 8100)  >9.02 | *P* value |
| --- | --- | --- | --- | --- | --- | --- |
| **Demographic** |  |  |  |  |  |  |
| Age, y | 55.3 ± 8.0 | 54.9 ± 8.2 | 55.7 ± 8.1 | 55.6 ± 7.9 | 55.0 ± 7.6 | <0.001 |
| Sex, n (%) |  |  |  |  |  | <0.001 |
| Male | 17947 (55.4) | 3683 (45.5) | 4106 (50.7) | 4664 (57.6) | 5494 (67.8) |  |
| Female | 14448 (44.6) | 4414 (54.5) | 3990 (49.3) | 3438 (42.4) | 2606 (32.2) |  |
| **Anthropometric Measures** |  |  |  |  |  |  |
| BMI, kg/m^2^ | 24.1  (22.1, 26.1) | 22.7  (20.9, 24.6) | 23.7  (21.8, 25.7) | 24.6  (22.7, 26.5) | 25.3  (23.4, 27.2) | <0.001 |
| WBC, ×10^9^/L | 6.0 ± 1.7 | 5.5 ± 1.6 | 5.8 ± 1.5 | 6.2 ± 1.6 | 6.6 ± 1.7 | <0.001 |
| PLT, ×10^9^/L | 246.0 ± 61.3 | 238.0 ± 61.8 | 245.0 ± 60.5 | 249.7 ± 59.5 | 251.2 ± 62.4 | <0.001 |
| Creatinine, μmol/L | 75.2 ± 22.5 | 71.9 ± 24.2 | 74.2 ± 21.0 | 76.5 ± 22.1 | 78.2 ± 22.0 | <0.001 |
| BUN, mmol/l | 5.0 ± 1.4 | 5.1 ± 1.5 | 5.0 ± 1.3 | 4.9 ± 1.3 | 4.9 ± 1.4 | <0.001 |
| MCV, fl | 88.7 ± 7.6 | 88.5 ± 8.4 | 88.8 ± 7.7 | 89.0 ± 7.3 | 88.5 ± 7.1 | <0.001 |
| Anemia, n (%) |  |  |  |  |  | <0.001 |
| No | 29765 (91.9) | 7017 (86.7) | 7415 (91.6) | 7590 (93.7) | 7743 (95.6) |  |
| Yes | 2630 (8.1) | 1080 (13.3) | 681 (8.4) | 512 (6.3) | 357 (4.4) |  |

Baseline descriptive statistics are presented as mean ± standard deviation (SD) for continuous variables with a normal distribution, median (interquartile range, IQR) for skewed continuous variables, and number (percentage, %) for categorical variables. Differences between TyG quartiles were analyzed by one-way ANOVA, the Kruskal-Wallis H test, or Pearson's chi-square test, as appropriate.

TyG, triglyceride-glucose, BMI Body mass index, WBC White blood cell count, PLT Platelet count, BUN Blood urea nitrogen, MCV Mean corpuscular volume, SD Standard deviation, IQR Interquartile range.

Supplementary Table S6. Baseline characteristics of participants in the Zhaotong clinical registry cohort according to TyG index quartiles.

| Variables | Total  (*N* = 10205) | Q1(*N* = 2551)  <8.45 | Q2(*N*= 2551)  8.45-8.86 | Q3(*N*= 2551)  8.86-9.30 | Q4 (*N* = 2552)  >9.30 | *P* value |
| --- | --- | --- | --- | --- | --- | --- |
| **Demographic** |  |  |  |  |  |  |
| Age,y | 54.9 ± 7.7 | 54.4 ± 8.1 | 55.0 ± 7.8 | 55.2 ± 7.6 | 54.9 ± 7.1 | 0.002 |
| Sex, n (%) |  |  |  |  |  | <0.001 |
| Male | 6352 (62.2) | 1182 (46.3) | 1441 (56.5) | 1699 (66.6) | 2030 (79.5) |  |
| Female | 3853 (37.8) | 1369 (53.7) | 1110 (43.5) | 852 (33.4) | 522 (20.5) |  |
| Marital status, n (%) |  |  |  |  |  | 0.845 |
| No | 36 (0.4) | 9 (0.4) | 7 (0.3) | 9 (0.4) | 11 (0.5) |  |
| Yes | 8212 (99.6) | 2015 (99.6) | 2060 (99.7) | 2043 (99.6) | 2094 (99.5) |  |
| **Health Status** |  |  |  |  |  |  |
| Hypertension, n (%) |  |  |  |  |  | <0.001 |
| No | 7836 (76.8) | 2188 (85.8) | 2041 (80) | 1930 (75.7) | 1677 (65.7) |  |
| Yes | 2369 (23.2) | 363 (14.2) | 510 (20) | 621 (24.3) | 875 (34.3) |  |
| DM, n (%) |  |  |  |  |  | <0.001 |
| No | 9418 (92.3) | 2491 (97.6) | 2455 (96.2) | 2359 (92.5) | 2113 (82.8) |  |
| Yes | 787 (7.7) | 60 (2.4) | 96 (3.8) | 192 (7.5) | 439 (17.2) |  |
| **Anthropometric Measures** |  |  |  |  |  |  |
| BMI, kg/m^2^ | 24.6  (22.5, 26.8) | 22.7  (20.9, 24.7) | 24.3  (22.3, 26.5) | 25.2  (23.3, 27.3) | 25.9  (24.0, 27.9) | <0.001 |
| WBC, ×10^9^/L | 5.9 ± 1.6 | 5.4 ± 1.5 | 5.8 ± 1.5 | 6.1 ± 1.6 | 6.3 ± 1.7 | <0.001 |
| PLT, ×10^9^/L | 232.3 ± 58.5 | 232.8 ± 59.8 | 236.6 ± 61.0 | 234.2 ± 57.5 | 225.5 ± 55.0 | <0.001 |
| MCV, fL | 92.6 ± 6.3 | 92.4 ± 6.8 | 92.5 ± 6.2 | 92.7 ± 6.0 | 92.9 ± 6.0 | 0.010 |
| RDW-SD, fL | 43.5 ± 2.8 | 43.5 ± 3.0 | 43.5 ± 2.8 | 43.5 ± 2.7 | 43.4 ± 2.7 | 0.648 |
| Creatinine, μmol/l | 72.3 ± 24.2 | 68.7 ± 18.3 | 71.4 ± 18.4 | 73.4 ± 18.1 | 75.6 ± 36.2 | <0.001 |
| BUN, mmol/l | 5.6 ± 1.6 | 5.6 ± 1.5 | 5.5 ± 1.5 | 5.5 ± 1.4 | 5.6 ± 1.7 | 0.062 |
| Anemia, n (%) |  |  |  |  |  | <0.001 |
| No | 9358 (91.7) | 2186 (85.7) | 2311 (90.6) | 2413 (94.6) | 2448 (95.9) |  |
| Yes | 847 (8.3) | 365 (14.3) | 240 (9.4) | 138 (5.4) | 104 (4.1) |  |

Baseline descriptive statistics are presented as mean ± standard deviation (SD) for continuous variables with a normal distribution, median (interquartile range, IQR) for skewed continuous variables, and number (percentage, %) for categorical variables. Differences between TyG quartiles were analyzed by one-way ANOVA, the Kruskal-Wallis H test, or Pearson's chi-square test, as appropriate.

TyG, triglyceride-glucose, BMI Body mass index, WBC White blood cell count, PLT Platelet count, MCV Mean corpuscular volume, RDW Red cell distribution width, BUN Blood urea nitrogen, DM Diabetes mellitus, SD Standard deviation, IQR Interquartile range.

Supplementary Table S7. Multivariable regression models (Models 1–4) assessing the association between the TyG index and the risk of incident anemia in the CHARLS longitudinal cohort.

| Categories | Model 1 | | Model 2 | | Model 3 | | Model 4 | |
| --- | --- | --- | --- | --- | --- | --- | --- | --- |
|  | OR (95%CI) | *P* value | OR (95%CI) | *P* value | OR (95%CI) | *P* value | OR (95%CI) | *P* value |
| TyG | 0.82 (0.74-0.91) | <0.001 | 0.86 (0.77-0.96) | 0.009 | 0.90 (0.80-1.01) | 0.081 | 0.87 (0.77-0.98) | 0.025 |
| TyG Quartile |  |  |  |  |  |  |  |  |
| Q1 (<8.3) | 1(Ref) |  | 1(Ref) |  | 1(Ref) |  | 1(Ref) |  |
| Q2 (8.3-8.6) | 0.92 (0.77-1.11) | 0.383 | 0.93 (0.77-1.12) | 0.416 | 0.93 (0.77-1.13) | 0.485 | 0.92 (0.76-1.11) | 0.395 |
| Q3 (8.6-9.1) | 0.80 (0.66-0.96) | 0.018 | 0.81 (0.67-0.98) | 0.034 | 0.85 (0.70-1.03) | 0.108 | 0.83 (0.68-1.01) | 0.047 |
| Q4 (>9.1) | 0.70 (0.58-0.85) | <0.001 | 0.75 (0.62-0.93) | 0.007 | 0.81 (0.66-1.01) | 0.068 | 0.77 (0.62-0.96) | 0.022 |
| *P* for trend |  | <0.001 |  | 0.005 |  | 0.037 |  | 0.014 |

CHARLS, China Health and Retirement Longitudinal Study, TyG, triglyceride-glucose, BMI: body mass index, WBC: white blood cell count, PLT: platelet count, MCV: mean corpuscular volume, hs-CRP: high-sensitivity C-reactive protein, HbA1c: glycated hemoglobin, BUN: blood urea nitrogen, DM: diabetes mellitus, CPD: chronic pulmonary disease, CKD: chronic kidney disease, OR: odds ratio, CI: confidence interval.

Model 1: Unadjusted

Model 2: adjusted for sex, age, race, marital status, residence, educational status, smoking status, drinking status, BMI

Model 3: adjusted for Model 2, additionally adjusted for Hypertension, DM, Cancer, CPD, Heart Diseases, Stroke, Depression, Arthritis, Dyslipidemia, Liver Diseases, CKD Chronic. gastritis, Asthma

Model 4: adjusted for Model 3, additionally adjusted for MCV, WBC, PLT, hs-CRP, HbA1c, BUN, and Creatinine

Supplementary Table S8. Multivariable regression models (Models 1–4) assessing the association between the TyG index and the risk of prevalent anemia in the CHARLS cross-sectional cohort.

| Categories | Model 1 | | Model 2 | | Model 3 | | Model 4 | |
| --- | --- | --- | --- | --- | --- | --- | --- | --- |
|  | OR (95%CI) | *P* value | OR (95%CI) | *P* value | OR (95%CI) | *P* value | OR (95%CI) | *P* value |
| TyG | 0.63 (0.58-0.69) | <0.001 | 0.70 (0.64-0.77) | <0.001 | 0.68 (0.62-0.75) | <0.001 | 0.76 (0.69-0.84) | <0.001 |
| TyG Quartile |  |  |  |  |  |  |  |  |
| Q1 (<8.3) | 1(Ref) |  | 1(Ref) |  | 1(Ref) |  | 1(Ref) |  |
| Q2 (8.3-8.6) | 0.73 (0.64-0.84) | <0.001 | 0.77 (0.67-0.88) | <0.001 | 0.76 (0.66-0.88) | <0.001 | 0.77 (0.67-0.89) | <0.001 |
| Q3 (8.6-9.1) | 0.57 (0.50-0.66) | <0.001 | 0.63 (0.54-0.73) | <0.001 | 0.62 (0.53-0.72) | <0.001 | 0.67 (0.57-0.78) | <0.001 |
| Q4 (>9.1) | 0.45 (0.39-0.52) | <0.001 | 0.54 (0.46-0.64) | <0.001 | 0.52 (0.43-0.62) | <0.001 | 0.63 (0.53-0.75) | <0.001 |
| *P* for trend |  | <0.001 |  | <0.001 |  | 0.001 |  | 0.003 |

CHARLS, China Health and Retirement Longitudinal Study, TyG, triglyceride-glucose, BMI: body mass index, WBC: white blood cell count, PLT: platelet count, MCV: mean corpuscular volume, hs-CRP: high-sensitivity C-reactive protein, HbA1c: glycated hemoglobin, BUN: blood urea nitrogen, DM: diabetes mellitus, CPD: chronic pulmonary disease, CKD: chronic kidney disease, OR: odds ratio, CI: confidence interval.

Model 1: Unadjusted

Model 2: adjusted for sex, age, race, marital status, residence, educational status, smoking status, drinking status, BMI

Model 3: adjusted for Model 2, additionally adjusted for Hypertension, DM, Cancer, CPD, Heart Diseases, Stroke, Depression, Arthritis, Dyslipidemia, Liver Diseases, CKD Chronic. gastritis, Asthma

Model 4: adjusted for Model 3, additionally adjusted for MCV, WBC, PLT, hs-CRP, HbA1c, BUN, and Creatinine

Supplementary Table S9. Multivariable regression models (Models 1–4) assessing the association between the TyG index and the risk of incident anemia in the ELSA cohort.

| Categories | Model 1 | | Model 2 | | Model 3 | | Model 4 | |
| --- | --- | --- | --- | --- | --- | --- | --- | --- |
|  | OR (95%CI) | *P* value | OR (95%CI) | *P* value | OR (95%CI) | *P* value | OR (95%CI) | *P* value |
| TyG | 0.84 (0.64-1.12) | 0.234 | 0.75 (0.55-1.02) | 0.065 | 0.73 (0.53-0.99) | 0.044 | 0.62 (0.45-0.86) | 0.005 |
| TyG Quartile |  |  |  |  |  |  |  |  |
| Q1 (<8.2) | 1(Ref) |  | 1(Ref) |  | 1(Ref) |  | 1(Ref) |  |
| Q2 (8.2-8.5) | 0.98 (0.68-1.42) | 0.925 | 0.88 (0.60-1.28) | 0.503 | 0.87 (0.59-1.27) | 0.464 | 0.86 (0.59-1.26) | 0.442 |
| Q3 (8.5-8.9) | 0.69 (0.47-1.03) | 0.072 | 0.58 (0.39-0.88) | 0.010 | 0.59 (0.39-0.90) | 0.014 | 0.58 (0.38-0.88) | 0.011 |
| Q4 (>8.9) | 0.80 (0.54-1.17) | 0.246 | 0.68 (0.45-1.02) | 0.065 | 0.66 (0.43-1.00) | 0.049 | 0.58 (0.37-0.89) | 0.014 |
| *P* for trend |  | 0.100 |  | 0.019 |  | 0.016 |  | 0.004 |

ELSA, English Longitudinal Study of Ageing; TyG, triglyceride-glucose; BMI: body mass index, WBC: white blood cell count, hs-CRP: high-sensitivity C-reactive protein, HbA1c: glycated hemoglobin, DM: diabetes mellitus, CPD: chronic pulmonary disease, CKD: chronic kidney disease, OR: odds ratio, CI: confidence interval.

Model 1: Unadjusted

Model 2: adjusted for age, sex, race, marital status, educational status, smoking status, drinking status, and BMI

Model 3: adjusted for Model 2 and additionally adjusted for Hypertension, DM, Cancer, CPD, Heart Diseases, Stroke, Arthritis

Model 4: adjusted for Model 3 and additionally adjusted for WBC, hs-CRP, HbA1c, antihypertensives, antidiabetics, respiratory drugs, antithrombotic drugs, and lipid-lowering drugs

Supplementary Table S10. Multivariable regression models (Models 1–4) assessing the association between the TyG index and the risk of prevalent anemia in the NHANES cohort.

| Categories | Model 1 | | Model 2 | | Model 3 | | Model 4 | |
| --- | --- | --- | --- | --- | --- | --- | --- | --- |
|  | OR (95%CI) | *P* value | OR (95%CI) | *P* value | OR (95%CI) | *P* value | OR (95%CI) | *P* value |
| TyG | 0.64 (0.57-0.72) | <0.001 | 0.62 (0.54-0.71) | <0.001 | 0.56 (0.48-0.64) | <0.001 | 0.61 (0.52-0.71) | <0.001 |
| TyG Quartile |  |  |  |  |  |  |  |  |
| Q1 (5.65-8.32) | 1(Ref) |  | 1(Ref) |  | 1(Ref) |  | 1(Ref) |  |
| Q2 (8.32-8.72) | 0.75 (0.62-0.91) | 0.003 | 0.72 (0.59-0.89) | 0.002 | 0.74 (0.60-0.91) | 0.005 | 0.80 (0.63-1.00) | 0.054 |
| Q3 (8.72-9.15) | 0.57 (0.46-0.70) | <0.001 | 0.55 (0.44-0.70) | <0.001 | 0.54 (0.43-0.68) | <0.001 | 0.64 (0.49-0.82) | <0.001 |
| Q4 (9.15-13.40) | 0.49 (0.40-0.59) | <0.001 | 0.46 (0.37-0.57) | <0.001 | 0.38 (0.30-0.49) | <0.001 | 0.44 (0.34-0.58) | <0.001 |
| *P* for trend |  | <0.001 |  | <0.001 |  | <0.001 |  | <0.001 |

NHANES, National Health and Nutrition Examination Survey, TyG, triglyceride-glucose; BMI: body mass index, PIR: poverty income ratio, DM: Diabetes Mellitus, CKD: Chronic Kidney Disease, COPD: Chronic Obstructive Pulmonary Disease, BUN: blood urea nitrogen, MCV: Mean Corpuscular Volume, VB12: vitamin B12, WBC: white blood cell, RDW: red blood cell distribution width, PLT: blood platelet, OR: odds ratio; CI: confidence interval

Model 1: not adjusted
Model 2: adjusted for Age, Sex, Race, marital status, educational status, smoking status, drinking status, BMI, PIR

Model 3: adjusted for model 2, additionally adjusted for Hypertension, Hyperlipidemia, DM, CKD, COPD, Cancer, Depression, Asthma, Arthritis, Heart disease

Model 4: adjusted for model 3, additionally adjusted for creatinine, BUN, MCV, VB12, iron, Total folate, WBC, RDW, PLT

Supplementary Table S11. Multivariable regression models (Models 1–4) assessing the association between the TyG index and the risk of prevalent anemia in the Ganzhou cohort.

| Categories | Model 1 | | Model 2 | | Model 3 | | Model 4 | |
| --- | --- | --- | --- | --- | --- | --- | --- | --- |
|  | OR (95%CI) | *P* value | OR (95%CI) | *P* value | OR (95%CI) | *P* value | OR (95%CI) | *P* value |
| TyG | 0.44 (0.41-0.48) | <0.001 | 0.56 (0.52-0.66) | <0.001 | 0.55 (0.51-0.60) | <0.001 | 0.53 (0.48-0.58) | <0.001 |
| TyG Quartile |  |  |  |  |  |  |  |  |
| Q1 (<8.25) | 1(Ref) |  | 1(Ref) |  | 1(Ref) |  | 1(Ref) |  |
| Q2 (8.25-8.62) | 0.60 (0.54-0.66) | <0.001 | 0.65 (0.59-0.73) | <0.001 | 0.65 (0.58-0.72) | <0.001 | 0.62 (0.54-0.70) | <0.001 |
| Q3 (8.62-9.02) | 0.44 (0.39-0.49) | <0.001 | 0.54 (0.48-0.61) | <0.001 | 0.53 (0.47-0.60) | <0.001 | 0.51 (0.45-0.59) | <0.001 |
| Q4 (>9.02) | 0.30 (0.26-0.34) | <0.001 | 0.43 (0.38-0.49) | <0.001 | 0.42 (0.37-0.48) | <0.001 | 0.39 (0.34-0.46) | <0.001 |
| *P* for trend |  | <0.001 |  | <0.001 |  | <0.001 |  | <0.001 |

TyG: triglyceride-glucose index, BMI: body mass index, BUN: blood urea nitrogen, WBC: white blood cell count, PLT: platelet count, MCV: mean corpuscular volume, OR: odds ratio, CI: confidence interval, Ref: reference.

Model 1: Unadjusted

Model 2: adjusted for sex, age, and BMI

Model 3: adjusted for Model 2 and additionally adjusted for creatinine and BUN

Model 4: adjusted for Model 3 and additionally adjusted for WBC, PLT, and MCV

Supplementary Table S12. Multivariable regression models (Models 1–4) assessing the association between the TyG index and the risk of prevalent anemia in the Zhaotong cohort.

| Categories | Model 1 | | Model 2 | | Model 3 | | Model 4 | |
| --- | --- | --- | --- | --- | --- | --- | --- | --- |
|  | OR (95%CI) | *P* value | OR (95%CI) | *P* value | OR (95%CI) | *P* value | OR (95%CI) | *P* value |
| TyG | 0.40 (0.35-0.46) | <0.001 | 0.58 (0.50-0.66) | <0.001 | 0.56 (0.48-0.64) | <0.001 | 0.58 (0.49-0.68) | <0.001 |
| TyG Quartile |  |  |  |  |  |  |  |  |
| Q1 (<8.45) | 1(Ref) |  | 1(Ref) |  | 1(Ref) |  | 1(Ref) |  |
| Q2 (8.45-8.86) | 0.62 (0.52-0.74) | <0.001 | 0.76 (0.63-0.91) | <0.001 | 0.75 (0.63-0.90) | <0.001 | 0.75 (0.60-0.93) | <0.001 |
| Q3 (8.86-9.30) | 0.34 (0.28-0.42) | <0.001 | 0.49 (0.40-0.61) | <0.001 | 0.48 (0.39-0.60) | <0.001 | 0.47 (0.37-0.61) | <0.001 |
| Q4 (>9.30) | 0.25 (0.20-0.32) | <0.001 | 0.47 (0.37-0.60) | <0.001 | 0.43 (0.33-0.56) | <0.001 | 0.46 (0.34-0.62) | <0.001 |
| *P* for trend |  | <0.001 |  | <0.001 |  | <0.001 |  | <0.001 |

TyG: triglyceride-glucose index, BMI: body mass index, DM: diabetes mellitus, BUN: blood urea nitrogen, WBC: white blood cell count, PLT: platelet count, MCV: mean corpuscular volume, RDW: red cell distribution width, OR: odds ratio, CI: confidence interval, Ref: reference.

Model 1: Unadjusted

Model 2: adjusted for sex, age, marital status, smoking status, drinking status, and BMI

Model 3: adjusted for Model 2 and additionally adjusted for hypertension, DM, Creatinine, and BUN

Model 4: adjusted for Model 3 and additionally adjusted for WBC, PLT, MCV, and RDW

Supplementary Figure S8. Flowchart of participant selection and censoring for the extended time-to-event analysis in the ELSA cohort

Supplementary Table S13. Association between the TyG index and incident anemia in the extended ELSA time-to-event analysis

| **Exposure** | **Participants, n** | **Events, n**  **(%)** | **Model 1** | | **Model 2** | | **Model 3** | | | **Model 4** | |
| --- | --- | --- | --- | --- | --- | --- | --- | --- | --- | --- | --- |
|  |  |  | **HR**  **(95% CI)** | ***P-*value** | **HR**  **(95% CI)** | ***P-*value** | **HR**  **(95% CI)** | ***P-*value** | | **HR**  **(95% CI)** | ***P-*value** |
| TyG | 2459 | 266 (10.8%) | 0.83  (0.64–1.09) | 0.183 | 0.71  (0.53–0.96) | 0.024 | 0.70  (0.51–0.95) | | 0.023 | 0.61 (0.43–0.87) | 0.006 |
| TyG Quartile |  |  |  |  |  |  |  | |  |  |  |
| Q1(≤8.225) | 627 | 79 (12.6%) | 1.00 (Ref.) |  | 1.00 (Ref.) |  | 1.00 (Ref.) | |  | 1.00 (Ref.) |  |
| Q2(8.225-8.544) | 608 | 72 (11.8%) | 0.93 (0.67–1.30) | 0.678 | 0.75  (0.54–1.04) | 0.080 | 0.73  (0.48–1.11) | | 0.141 | 0.71  (0.46–1.09) | 0.119 |
| Q3(8.544-8.892) | 611 | 53 (8.7%) | 0.66 (0.46–0.94) | 0.023 | 0.56  (0.37–0.85) | 0.007 | 0.57  (0.37–0.88) | | 0.011 | 0.55 (0.32–0.94) | 0.030 |
| Q4>8.892 | 613 | 62 (10.1%) | 0.78  (0.57–1.07) | 0.118 | 0.62  (0.44–0.88) | 0.007 | 0.60  (0.38–0.92) | | 0.021 | 0.53 (0.33–0.84) | 0.007 |
| *P* for trend |  |  |  | 0.082 |  | 0.005 |  | | 0.009 |  | 0.004 |

ELSA, English Longitudinal Study of Ageing; TyG, triglyceride-glucose; BMI: body mass index, WBC: white blood cell count, hs-CRP: high-sensitivity C-reactive protein, HbA1c: glycated hemoglobin, DM: diabetes mellitus, CPD: chronic pulmonary disease, CKD: chronic kidney disease, HR, hazard ratio, CI: confidence interval.

Model 1: Unadjusted

Model 2: adjusted for age, sex, race, marital status, educational status, smoking status, drinking status, and BMI

Model 3: adjusted for Model 2 and additionally adjusted for Hypertension, DM, Cancer, CPD, Heart Diseases, Stroke, Arthritis

Model 4: adjusted for Model 3 and additionally adjusted for WBC, hs-CRP, HbA1c, antihypertensives, antidiabetics, respiratory drugs, antithrombotic drugs, and lipid-lowering drugs

Hazard ratios (HRs) and 95% confidence intervals (CIs) were estimated using interval-censored proportional hazards models, because incident anemia was ascertained only at discrete study waves and the exact event time was therefore unknown.

Supplementary Figure S9. Dose–response association between the TyG index and incident anemia in the extended ELSA time-to-event analysis.

Restricted cubic spline analysis was based on the fully adjusted interval-censored proportional hazards model. The solid line and shaded area represent hazard ratios and 95% confidence intervals, respectively, and the histogram shows the distribution of the TyG index. The reference value was TyG = 8.538. The overall association was statistically supported (*P* for overall = 0.013), with no evidence of nonlinearity (*P* for non-linearity = 0.249).

Supplementary Table S14. Proportional hazards assumption assessment for the interval-censored proportional hazards model

| **Variable** | **chisq** | **df** | ***P* value** |
| --- | --- | --- | --- |
| Age | 1.120 | 1.0 | 0.290 |
| Sex | 1.323 | 1.0 | 0.250 |
| Marital status | 0.000 | 1.0 | 0.983 |
| Educational status | 0.002 | 1.0 | 0.962 |
| Race | 0.325 | 1.0 | 0.568 |
| Smoking status | 0.010 | 1.0 | 0.922 |
| Drinking status | 0.015 | 1.0 | 0.901 |
| Hypertension | 0.388 | 1.0 | 0.534 |
| DM | 1.173 | 1.0 | 0.279 |
| Cancer | 1.821 | 1.0 | 0.177 |
| CPD | 1.650 | 1.0 | 0.199 |
| Stroke | 1.340 | 1.0 | 0.247 |
| Arthritis | 0.167 | 1.0 | 0.683 |
| Heart disease | 0.068 | 1.0 | 0.794 |
| Antihypertensives | 0.023 | 1.0 | 0.880 |
| Antidiabetics | 2.793 | 1.0 | 0.095 |
| Respiratory drugs | 0.441 | 1.0 | 0.506 |
| Antithrombotics | 0.391 | 1.0 | 0.532 |
| Lipid-lowering drugs | 2.277 | 1.0 | 0.131 |
| WBC | 0.016 | 1.0 | 0.899 |
| BMI | 0.133 | 1.0 | 0.716 |
| HsCRP | 0.821 | 1.0 | 0.365 |
| Hba1c | 0.068 | 1.0 | 0.795 |
| GLOBAL | 25.957 | 29.0 | 0.628 |

The proportional hazards assumption for the interval-censored proportional hazards model was assessed using Schoenfeld residuals. No evidence of violation was observed for individual covariates or for the model globally (global *P* = 0.628).

DM, diabetes mellitus; CPD, chronic pulmonary disease; hs-CRP, high-sensitivity C-reactive protein; WBC, white blood cell count; HbA1c, glycated hemoglobin; chisq, chi-square statistic.

| Categories | Model 1 | | Model 2 | | Model 3 | | Model 4 | |
| --- | --- | --- | --- | --- | --- | --- | --- | --- |
|  | HR (95%CI) | *P* value | HR (95%CI) | *P* value | HR (95%CI) | *P* value | HR (95%CI) | *P* value |
| TyG | 0.80(0.61~1.05) | 0.110 | 0.70 (0.51~0.95) | 0.020 | 0.68 (0.50~0.93) | 0.014 | 0.64 (0.47~0.88) | 0.006 |
| TyG Quartile |  |  |  |  |  |  |  |  |
| Q1 | 1(Ref) |  | 1(Ref) |  | 1(Ref) |  | 1(Ref) |  |
| Q2 | 0.86 (0.60~1.22) | 0.388 | 0.78 (0.54~1.12) | 0.173 | 0.76 (0.53~1.09) | 0.133 | 0.75 (0.52~1.09) | 0.134 |
| Q3 | 0.61 (0.41~0.90) | 0.014 | 0.51 (0.34~0.76) | 0.001 | 0.53 (0.35~0.80) | 0.002 | 0.52 (0.34~0.78) | 0.002 |
| Q4 | 0.76 (0.52~1.10) | 0.140 | 0.64 (0.43~0.96) | 0.029 | 0.61 (0.40~0.91) | 0.015 | 0.58 (0.38~0.88) | 0.010 |
| *P* for trend |  | 0.051 |  | 0.007 |  | 0.005 |  | 0.003 |

Supplementary Table S15. Complete-follow-up sensitivity analysis of the association between the TyG index and incident anemia in the ELSA cohort

Data are presented as hazard ratios (HRs) with 95% confidence intervals (CIs). This sensitivity analysis was restricted to the complete extended-follow-up cohort, including participants who developed incident anemia during follow-up or had valid anemia ascertainment through Wave 8. Participants who remained anemia-free at Wave 6 but lacked a valid Wave 8 hemoglobin measurement were excluded. Interval-censored proportional hazards models used the same sequential adjustment strategy as the primary extended time-to-event analysis. Q1 was the reference category for quartile analyses.

ELSA, English Longitudinal Study of Ageing; TyG, triglyceride-glucose; BMI: body mass index, WBC: white blood cell count, hs-CRP: high-sensitivity C-reactive protein, HbA1c: glycated hemoglobin, DM: diabetes mellitus, CPD: chronic pulmonary disease, CKD: chronic kidney disease, HR: hazard ratio, CI: confidence interval.

Model 1: Unadjusted

Model 2: adjusted for age, sex, race, marital status, educational status, smoking status, drinking status, and BMI

Model 3: adjusted for Model 2 and additionally adjusted for Hypertension, DM, Cancer, CPD, Heart Diseases, Stroke, Arthritis

Model 4: adjusted for Model 3 and additionally adjusted for WBC, hs-CRP, HbA1c, antihypertensives, antidiabetics, respiratory drugs, antithrombotic drugs, and lipid-lowering drugs

Supplementary Figure S10. Stratified associations between the TyG index and prevalent anemia across four cross-sectional cohorts.

Forest plots show the adjusted associations of the TyG index with prevalent anemia across demographic and clinical strata in the CHARLS cross-sectional cohort (A), NHANES (B), Ganzhou (C), and Zhaotong (D). Effect estimates are presented as ORs with 95% CIs per 1-unit increase in the TyG index. Models were adjusted for the covariates included in the fully adjusted cohort-specific models, excluding the corresponding stratification variable where applicable. Between-stratum heterogeneity was assessed using likelihood-ratio tests comparing models with and without the corresponding TyG-by-subgroup term. NHANES analyses additionally accounted for the complex survey design.
BMI, body mass index; CHARLS, China Health and Retirement Longitudinal Study; CI, confidence interval; CKD, chronic kidney disease; MCV, mean corpuscular volume; NHANES, National Health and Nutrition Examination Survey; OR, odds ratio; TyG, triglyceride–glucose index.

| Supplementary Table S16. Exploratory comparison of candidate intermediate markers in the association between the TyG index and incident anemia in the CHARLS longitudinal cohort. | | | | | | |
| --- | --- | --- | --- | --- | --- | --- |
| Candidate intermediate marker | Total association | Direct association | Indirect association | 95% CI for indirect association | Proportion statistically accounted for | Statistical support |
| 1. Cystatin C | -0.033 | -0.028 | -0.0053 | (-0.0098, -0.0011) | 16.33% | Significant |
| *(Renal Function)* |  |  |  | *(Does not cross 0)* |  |  |
| 2. hs-CRP | -0.0307 | -0.0306 | -0.0001 | (-0.0012, 0.0008) | 0.30% | *Null* |
| *(Inflammation)* |  |  |  | *(Crosses 0)* |  |  |
| 3. WBC | -0.0276 | -0.03 | -0.0023 | (-0.0005, 0.0051) | 8.46% | *Null* |
| *(Infection/Inflammation)* |  |  |  | *(Crosses 0)* |  |  |
| 4. CKD Status | -0.0283 | -0.0264 | -0.002 | (-0.0066, 0.0007) | 6.91% | *Null* |
| *(Disease Diagnosis)* |  |  |  | *(Crosses 0)* |  |  |

Exploratory mediation models were fitted separately for each candidate intermediate marker. Total, direct, and indirect associations were estimated, with 95% confidence intervals for indirect associations obtained using 1,000 bootstrap resamples. Statistical support for an indirect association was defined by a 95% confidence interval excluding zero. Because the TyG index and candidate intermediate markers were measured concurrently at baseline, these analyses do not establish temporal or causal mediation and should be interpreted as exploratory pathway analyses. The proportion statistically accounted for was considered descriptive and was not used to infer causal contribution.

CHARLS, China Health and Retirement Longitudinal Study; TyG, triglyceride-glucose; hs-CRP, high-sensitivity C-reactive protein; WBC, white blood cell count; CKD, chronic kidney disease; CI, confidence interval; TE, Total Effect; DE, Direct Effect; IE, Indirect Effect.

Sensitivity Analysis Stratified by Anemia Morphology (MCV Categories)

General Note for Tables S17–S18: Sensitivity analyses were conducted according to MCV-defined erythrocyte morphology: microcytic (MCV <80 fL), normocytic (MCV 80–100 fL), and macrocytic (MCV >100 fL). Associations between the TyG index and anemia were estimated using the same sequential multivariable regression framework as in the primary analyses. The inverse association was observed in the microcytic and normocytic categories in both CHARLS and NHANES, whereas estimates for macrocytic anemia were attenuated and not statistically supported. These analyses were intended to characterize heterogeneity according to erythrocyte morphology and do not establish anemia etiology or underlying mechanisms.

Supplementary Table S17. Sensitivity analysis of the association between the TyG index and prevalent anemia, stratified by MCV categories in the CHARLS cross-sectional cohort.

|  | Categories | Model 1 | | Model 2 | | Model 3 | | Model 4 | |
| --- | --- | --- | --- | --- | --- | --- | --- | --- | --- |
|  |  | OR (95%CI) | *P* value | OR (95%CI) | *P* value | OR (95%CI) | *P* value | OR (95%CI) | *P* value |
| MCV (<80 fL) | TyG | 0.53 (0.43~0.66) | <0.001 | 0.58 (0.47~0.73) | <0.001 | 0.59 (0.46~0.75) | <0.001 | 0.63 (0.49~0.80) | <0.001 |
|  | TyG quartiles |  |  |  |  |  |  |  |  |
|  | Q1 (<8.20) | 1(Ref) |  | 1(Ref) |  | 1(Ref) |  | 1(Ref) |  |
|  | Q2 (8.20-8.55) | 0.59 (0.41~0.84) | 0.003 | 0.63 (0.44~0.91) | 0.014 | 0.62 (0.42~0.90) | 0.013 | 0.65 (0.44~0.96) | 0.03 |
|  | Q3 (8.55-9.00) | 0.46 (0.32~0.66) | <0.001 | 0.49 (0.34~0.72) | <0.001 | 0.50 (0.34~0.75) | 0.001 | 0.51 (0.34~0.76) | 0.001 |
|  | Q4(>9.00) | 0.28 (0.19~0.41) | <0.001 | 0.33 (0.22~0.49) | <0.001 | 0.33 (0.21~0.52) | <0.001 | 0.37 (0.23~0.57) | <0.001 |
|  | *P* for trend |  | <0.001 |  | <0.001 |  | <0.001 |  | <0.001 |
| MCV (80-100 fL) | TyG | 0.64 (0.58~0.7) | <0.001 | 0.71 (0.64~0.79) | <0.001 | 0.69 (0.62~0.77) | <0.001 | 0.77 (0.69~0.87) | <0.001 |
|  | TYG quartiles |  |  |  |  |  |  |  |  |
|  | Q1 (<8.24) | 1(Ref) |  | 1(Ref) |  | 1(Ref) |  | 1(Ref) |  |
|  | Q2 (8.24-8.63) | 0.73 (0.62~0.85) | <0.001 | 0.76 (0.64~0.89) | 0.001 | 0.75 (0.64~0.89) | 0.001 | 0.77 (0.65~0.91) | 0.002 |
|  | Q3 (8.63-9.08) | 0.57 (0.48~0.67) | <0.001 | 0.62 (0.52~0.74) | <0.001 | 0.62 (0.52~0.74) | <0.001 | 0.67 (0.56~0.80) | <0.001 |
|  | Q4(>9.08) | 0.46 (0.39~0.56) | <0.001 | 0.56 (0.47~0.68) | <0.001 | 0.55 (0.45~0.67) | <0.001 | 0.65 (0.53~0.79) | <0.001 |
|  | *P* for trend |  | <0.001 |  | <0.001 |  | <0.001 |  | <0.001 |
| MCV（>100 fL） | TyG | 0.86 (0.66~1.11) | 0.249 | 1.02 (0.78~1.34) | 0.878 | 1.06 (0.78~1.43) | 0.712 | 1.07 (0.79~1.45) | 0.672 |
|  | TyG quartiles |  |  |  |  |  |  |  |  |
|  | Q1 (<8.17) | 1(Ref) |  | 1(Ref) |  | 1(Ref) |  | 1(Ref) |  |
|  | Q2 (8.17-8.58) | 0.73 (0.45~1.17) | 0.192 | 0.8 (0.49~1.3) | 0.363 | 0.81 (0.49~1.33) | 0.4 | 0.76 (0.45~1.26) | 0.285 |
|  | Q3 (8.58-9.01) | 0.88 (0.55~1.39) | 0.571 | 0.99 (0.62~1.6) | 0.974 | 1.03 (0.63~1.68) | 0.902 | 1.04 (0.63~1.72) | 0.867 |
|  | Q4(>9.01) | 0.75 (0.47~1.20) | 0.23 | 1.00 (0.60~1.64) | 0.986 | 1.05 (0.62~1.80) | 0.852 | 1.04 (0.60~1.81) | 0.885 |
|  | *P* for trend |  | 0.355 |  | 0.827 |  | 0.684 |  | 0.656 |

MCV: mean corpuscular volume, BMI: body mass index, WBC: white blood cell count, PLT: platelet count, hs-CRP: high-sensitivity C-reactive protein, HbA1c: glycated hemoglobin, BUN: blood urea nitrogen, Creatinine: creatinine, DM: diabetes mellitus, CPD: chronic pulmonary disease, CKD: chronic kidney disease, OR: odds ratio, CI: confidence interval.

Model 1: Unadjusted

Model 2: adjusted for sex, age, race, marital status, residence, educational status, smoking status, drinking status, BMI

Model 3: adjusted for Model 2, additionally adjusted for Hypertension, DM, Cancer, CPD, Heart Diseases, Stroke, Depression, Arthritis, Dyslipidemia, Liver Diseases, CKD Chronic. gastritis, Asthma

Model 4: adjusted for Model 3, additionally adjusted for WBC, PLT, hs-CRP, HbA1c, BUN, and Creatinine

TyG quartile cut points were derived separately within each MCV category.

Supplementary Table S18. Sensitivity analysis of the association between the TyG index and prevalent anemia, stratified by MCV categories in the NHANES cohort.

|  | Categories | Model 1 | | Model 2 | | Model 3 | | Model 4 | |
| --- | --- | --- | --- | --- | --- | --- | --- | --- | --- |
|  |  | OR (95%CI) | *P* value | OR (95%CI) | *P* value | OR (95%CI) | *P* value | OR (95%CI) | *P* value |
| MCV (<80 fL) | TyG | 0.47 (0.34~0.65) | <0.001 | 0.44 (0.30~0.63) | <0.001 | 0.48 (0.32~0.71) | <0.001 | 0.45 (0.29~0.69) | <0.001 |
|  | TyG quartiles |  |  |  |  |  |  |  |  |
|  | Q1 (<8.18) | 1(Ref) |  | 1(Ref) |  | 1(Ref) |  | 1(Ref) |  |
|  | Q2 (8.18-8.55) | 0.93 (0.49~1.76) | 0.829 | 0.78 (0.42~1.48) | 0.448 | 0.85 (0.45~1.61) | 0.611 | 1.08 (0.51~2.30) | 0.843 |
|  | Q3 (8.55-8.99) | 0.59 (0.30~1.16) | 0.126 | 0.51 (0.26~1.01) | 0.053 | 0.61 (0.31~1.20) | 0.151 | 0.71 (0.34~1.46) | 0.343 |
|  | Q4(>8.99) | 0.32 (0.17~0.59) | <0.001 | 0.27 (0.14~0.52) | <0.001 | 0.34 (0.16~0.70) | 0.004 | 0.39 (0.17~0.88) | 0.025 |
|  | *P* for trend |  | <0.001 |  | <0.001 |  | 0.004 |  | 0.018 |
| MCV (80-100 fL) | TyG | 0.69 (0.60~0.79) | <0.001 | 0.64 (0.50~0.81) | <0.001 | 0.56 (0.47~0.66) | <0.001 | 0.65 (0.55~0.77) | <0.001 |
|  | TyG quartiles |  |  |  |  |  |  |  |  |
|  | Q1 (<8.33) | 1(Ref) |  | 1(Ref) |  | 1(Ref) |  | 1(Ref) |  |
|  | Q2 (8.33-8.73) | 0.66 (0.53~0.83) | <0.001 | 0.64 (0.50~0.81) | <0.001 | 0.63 (0.49~0.80) | <0.001 | 0.71 (0.55~0.91) | 0.008 |
|  | Q3 (8.73-9.16) | 0.62 (0.49~0.78) | <0.001 | 0.60 (0.46~0.76) | <0.001 | 0.55 (0.43~0.71) | <0.001 | 0.68 (0.52~0.89) | 0.006 |
|  | Q4(>9.16) | 0.54 (0.43~0.67) | <0.001 | 0.51 (0.40~0.66) | <0.001 | 0.39 (0.30~0.50) | <0.001 | 0.48 (0.36~0.64) | <0.001 |
|  | *P* for trend |  | <0.001 |  | <0.001 |  | <0.001 |  | <0.001 |
| MCV (>100 fL) | TyG | 0.92 (0.67~1.28) | 0.629 | 0.89 (0.56~1.42) | 0.629 | 0.78 (0.49~1.24) | 0.289 | 1.10 (0.55~2.21) | 0.777 |
|  | TyG quartiles |  |  |  |  |  |  |  |  |
|  | Q1 (<8.16) | 1(Ref) |  | 1(Ref) |  | 1(Ref) |  | 1(Ref) |  |
|  | Q2 (8.16-8.63) | 2.12 (0.88~5.09) | 0.092 | 2.03 (0.64~6.39) | 0.221 | 1.21 (0.37~4.02) | 0.747 | 0.78 (0.20~2.96) | 0.706 |
|  | Q3 (8.63-9.04) | 1.50 (0.55~4.08) | 0.426 | 1.57 (0.43~5.64) | 0.378 | 0.96 (0.32~2.95) | 0.949 | 1.19 (0.31~4.48) | 0.796 |
|  | Q4(>9.04) | 0.87 (0.36~2.08) | 0.745 | 0.79 (0.29~2.16) | 0.644 | 0.48 (0.15~1.47) | 0.192 | 0.61 (0.18~2.08) | 0.418 |
|  | *P* for trend |  | 0.687 |  | 0.700 |  | 0.180 |  | 0.672 |

BMI: body mass index, PIR: poverty income ratio, DM: Diabetes, CKD: Chronic Kidney Disease, COPD: Chronic Obstructive Pulmonary Disease, Cr: creatinine, BUN: blood urea nitrogen, MCV: Mean Corpuscular Volume, VB12: vitamin B12, WBC: white blood cell, RDW: red blood cell distribution width, PLT: blood platelet, OR: odds ratio; CI: confidence interval

Model 1: not adjusted
Model 2: adjusted for Age, Sex, Race, marital status, educational status, smoking status, drinking status, BMI, PIR

Model 3: adjusted for model 2, additionally adjusted for Hypertension, Hyperlipidemia, DM, CKD, COPD, Cancer, Depression, Asthma, Arthritis, Heart disease

Model 4: adjusted for model 3, additionally adjusted for Cr, BUN, VB12, iron, Total folate, WBC, RDW, PLT

TyG quartile cut points were derived separately within each MCV category.

All NHANES regression analyses accounted for survey weights, strata, and primary sampling units.

Sensitivity Analysis by Excluding Specific High-Risk or Morbid Subpopulations

General Note for Tables S19–S20: Sensitivity analyses were performed in the CHARLS and ELSA longitudinal cohorts after excluding selected subpopulations with baseline conditions that could potentially influence both metabolic status and anemia risk. These analyses included exclusions based on underweight status, poor self-rated health, and selected baseline comorbidities available in each cohort. Associations were re-estimated using the fully adjusted cohort-specific models. The resulting estimates were broadly similar to those observed in the primary longitudinal analyses.

Supplementary Table S19. Sensitivity analysis of the association between the TyG index and incident anemia after excluding specific subpopulations in the CHARLS longitudinal cohort.

| Variables | Excluding individuals with CPD | Excluding participants with poor self-rated health | Excluding individuals with Stroke | Exclude individuals with a BMI less than 18.5 |
| --- | --- | --- | --- | --- |
| Total N/ | 5358/941 (17.60) | 5599/995 (17.80) | 5782/1019 (17.60) | 5614/960 (17.10) |
| Events (%) |  |  |  |  |
| TyG Continuous | | | | |
| Model 4  (Fully adjusted) | 0.86 (0.76-0.98) | 0.86 (0.76-0.98) | 0.87 (0.77-0.98) | 0.88 (0.77-0.98) |
| *P* | 0.024 | 0.024 | 0.028 | 0.041 |
| TyG Quartiles | | | | |
| Model 4  (Fully adjusted) | OR (95%CI) | OR (95%CI) | OR (95%CI) | OR (95%CI) |
| Q1(Ref) | 1 | 1 | 1 | 1 |
| Q2 | 0.89 (0.73-1.09) | 0.95 (0.78-1.16) | 0.97 (0.80-1.17) | 0.87 (0.72-1.06) |
| Q3 | 0.80 (0.65-0.99) | 0.87 (0.71-1.07) | 0.85 (0.70-1.04) | 0.80 (0.65-0.98) |
| Q4 | 0.77 (0.62-0.97) | 0.78 (0.62-0.98) | 0.80 (0.64-0.99) | 0.78 (0.63-0.99) |
| *P* for trend | 0.015 | 0.025 | 0.027 | 0.03 |

Data are presented as Odds Ratios (OR) and 95% Confidence Intervals (CI). Model 4 represents the fully adjusted model; covariates were strictly selected based on Directed Acyclic Graphs (DAGs) and tailored to each cohort, broadly encompassing sociodemographic factors, lifestyle habits, and comprehensive clinical comorbidities.

TyG, Triglyceride-Glucose; OR, Odds Ratio; CI, Confidence Interval; CPD, Chronic Pulmonary Disease; BMI, Body Mass Index; Ref, Reference; CHARLS, China Health and Retirement Longitudinal Study

Supplementary Table S20. Sensitivity analysis of the association between the TyG index and incident anemia after excluding specific subpopulations in the ELSA cohort.

| Variables | Excluding individuals with diabetes | Excluding participants with poor self-rated health | Excluding individuals with arthritis | Exclude individuals with a BMI less than 18.5 |
| --- | --- | --- | --- | --- |
| Total N/ | 2397/214 (8.90) | 2391/210 (8.80) | 1696/140 (8.30) | 2443/222 (9.10) |
| Events (%) |  |  |  |  |
| TyG Continuous | | | | |
| Model 4 | 0.60 (0.43-0.84) | 0.62 (0.44-0.87) | 0.55 (0.36-0.84) | 0.62 (0.45-0.87) |
| (Fully adjusted) |  |  |  |  |
| *P* | 0.003 | 0.006 | 0.005 | 0.005 |
| TyG Quartiles | | | | |
| Model 4 | OR (95%CI) | OR (95%CI) | OR (95%CI) | OR (95%CI) |
| (Fully adjusted) |  |  |  |  |
| Q1(Ref) | 1 | 1 | 1 | 1 |
| Q2 | 0.82 (0.55-1.12) | 0.91 (0.61-1.35) | 0.66 (0.40-1.07) | 0.86 (0.58-1.26) |
| Q3 | 0.60 (0.39-0.91) | 0.67 (0.43-1.02) | 0.62 (0.37-1.04) | 0.59 (0.38-0.90) |
| Q4 | 0.57 (0.37-0.90) | 0.58 (0.36-0.91) | 0.52 (0.30-0.90) | 0.57 (0.37-0.88) |
| *P* for trend | 0.006 | 0.008 | 0.021 | 0.004 |

Data are presented as Odds Ratios (OR) and 95% Confidence Intervals (CI). Model 4 represents the fully adjusted model; covariates were strictly selected based on Directed Acyclic Graphs (DAGs) and tailored to each cohort, broadly encompassing sociodemographic factors, lifestyle habits, and comprehensive clinical comorbidities.

TyG, Triglyceride-Glucose; OR, Odds Ratio; CI, Confidence Interval; BMI, Body Mass Index; Ref, Reference; ELSA, English Longitudinal Study of Ageing

Sensitivity Analyses Regarding Data Processing and Outlier Management

| Supplementary Table S21. Sensitivity analysis of the association between the TyG index and anemia across six cohorts after excluding extreme TyG values. | | | | | | |
| --- | --- | --- | --- | --- | --- | --- |
| Variables | CHARLS cross | CHARLS long | NHANES | ELSA | Ganzhou | Zhaotong |
| Total N /  Events% | 10624/1705  (16.00) | 5867/1039  (17.70) | 14414/1692  (7.95*) | 2433/224  (9.20) | 32071/2594  (8.10) | 10101/832  (8.20) |
| TyG Continuous | | | | | | |
| Model 4  (Fully Adj) | 0.75 (0.67-0.83) | 0.85 (0.75-0.97) | 0.63 (0.53-0.74) | 0.63 (0.45-0.89) | 0.51 (0.46-0.56) | 0.58 (0.49-0.69) |
| *P* value | <0.001 | 0.015 | <0.001 | 0.008 | <0.001 | <0.001 |
| TyG Quartiles | | | | | | |
| Model 4  (Fully Adj) | OR (95%CI) | OR (95%CI) | OR (95%CI) | OR (95%CI) | OR (95%CI) | OR (95%CI) |
| Q1(Ref) | 1.00 | 1.00 | 1.00 | 1.00 | 1.00 | 1.00 |
| Q2 | 0.75 (0.65-0.87) | 0.89 (0.73-1.07) | 0.82 (0.65-1.03) | 0.86 (0.58-1.26) | 0.62 (0.55-0.70) | 0.76 (0.61-0.94) |
| Q3 | 0.67 (0.58-0.78) | 0.81 (0.66-0.99) | 0.65 (0.50-0.84) | 0.56 (0.37-0.86) | 0.51 (0.45-0.59) | 0.48 (0.37-0.62) |
| Q4 | 0.62 (0.52-0.73) | 0.78 (0.63-0.97) | 0.46 (0.35-0.60) | 0.57 (0.37-0.89) | 0.40 (0.34-0.46) | 0.47 (0.35-0.64) |
| *P* for trend | 0.003 | 0.016 | <0.001 | 0.003 | <0.001 | <0.001 |

*In the descriptive row 'Total N / Events N (%)', the percentage for the NHANES cohort represents the weighted prevalence of anemia, whereas percentages for other cohorts are unweighted crude rates.

To minimize the potential influence of extreme outliers, a sensitivity analysis was performed by excluding participants with TyG index values falling in the top 0.5% and bottom 0.5% of the distribution. Data are presented as multivariable-adjusted odds ratios (ORs) and 95% confidence intervals (CIs) from the fully adjusted model (Model 4).

TyG, Triglyceride-Glucose; OR, Odds Ratio; CI, Confidence Interval; Ref, Reference; CHARLS, China Health and Retirement Longitudinal Study; NHANES, National Health and Nutrition Examination Survey; ELSA, English Longitudinal Study of Ageing.

| Supplementary Table S22. Sensitivity analysis of the association between the TyG index and the risk of anemia utilizing a complete-case analysis approach. | | | | | | |
| --- | --- | --- | --- | --- | --- | --- |
| Variables | CHARLS cross | CHARLS long | NHANES | ELSA | Ganzhou | Zhaotong |
| Total *N*/  Events *N* (%) | 7344/1161 (15.80) | 4609/797 (17.30) | 9916/1057 (7.53*) | 2081/182 (8.70) | 32259/2620 (8.10) | 8232/683 (8.30) |
| TyG Continuous | | | | | | |
| Model 4  (Fully adjusted) | 0.82 (0.73-0.92) | 0.87 (0.76-0.99) | 0.61 (0.42-0.87) | 0.57 (0.40-0.83) | 0.53 (0.48-0.58) | 0.48 (0.40-0.57) |
| *P* | 0.001 | 0.041 | 0.012 | 0.003 | <0.001 | <0.001 |
| TyG Quartiles | | | | | | |
| Model 4  (Fully adjusted) | OR (95%CI) | OR (95%CI) | OR (95%CI) | OR (95%CI) | OR (95%CI) | OR (95%CI) |
| Q1(Ref) | 1.00 | 1.00 | 1.00 | 1.00 | 1.00 | 1.00 |
| Q2 | 0.81 (0.68-0.97) | 0.92 (0.74-1.14) | 0.97 (0.64-1.46) | 0.69 (0.45-1.05) | 0.62 (0.55-0.71) | 0.70 (0.56-0.88) |
| Q3 | 0.82 (0.68-0.98) | 0.85 (0.68-1.06) | 0.83 (0.45-1.51) | 0.53 (0.33-0.83) | 0.52 (0.45-0.59) | 0.39 (0.29-0.51) |
| Q4 | 0.67 (0.54-0.83) | 0.77 (0.60-0.97) | 0.36 (0.18-0.70) | 0.49 (0.30-0.79) | 0.40 (0.34-0.46) | 0.36 (0.26-0.49) |
| *P* for trend | <0.001 | 0.034 | 0.006 | 0.002 | <0.001 | <0.001 |

*In the descriptive row 'Total N / Events N (%)', the percentage for the NHANES cohort represents the weighted prevalence of anemia, whereas percentages for other cohorts are unweighted crude rates.

To assess the robustness of the findings against potential biases introduced by missing data imputation, a strict complete-case analysis was conducted. Data are presented as odds ratios (ORs) and 95% confidence intervals (CIs) derived from the fully adjusted multivariable models (Model 4).

TyG, Triglyceride-Glucose; OR, Odds Ratio; CI, Confidence Interval; Ref, Reference; CHARLS, China Health and Retirement Longitudinal Study; NHANES, National Health and Nutrition Examination Survey; ELSA, English Longitudinal Study of Ageing

| Supplementary Table S23. Sensitivity analysis of the association between the TyG index and the risk of anemia across six cohorts, utilizing tertile categorization. | | | | | | |
| --- | --- | --- | --- | --- | --- | --- |
| Variables | CHARLS cross | CHARLS long | NHANES | ELSA | Ganzhou | Zhaotong |
| Model 4  (Fully Adj) | OR (95%CI) | OR (95%CI) | OR (95%CI) | OR (95%CI) | OR (95%CI) | OR (95%CI) |
| T1(Ref) | 1.00 | 1.00 | 1.00 | 1.00 | 1.00 | 1.00 |
| T2 | 0.72 (0.63-0.82) | 0.92 (0.78-1.09) | 0.70 (0.57-0.87) | 0.68 (0.48-0.96) | 0.59 (0.52-0.66) | 0.65 (0.53-0.80) |
| T3 | 0.67 (0.55-0.74) | 0.78 (0.65-0.94) | 0.58 (0.47-0.71) | 0.59 (0.40-0.86) | 0.45 (0.39-0.51) | 0.48 (0.37-0.62) |
| *P* for trend | <0.001 | 0.011 | <0.001 | 0.006 | <0.001 | <0.001 |

To rigorously verify that the observed protective associations were not artifacts of the chosen categorization strategy (i.e., quartiles), a sensitivity analysis was conducted by re-stratifying the continuous TyG index into tertiles (T1–T3). Data are presented as multivariable-adjusted odds ratios (ORs) and 95% confidence intervals (CIs) derived from the fully adjusted models (Model 4). The lowest tertile (T1) served as the reference group.

TyG, Triglyceride-Glucose; OR, Odds Ratio; CI, Confidence Interval; Ref, Reference; CHARLS, China Health and Retirement Longitudinal Study; NHANES, National Health and Nutrition Examination Survey; ELSA, English Longitudinal Study of Ageing

Sensitivity Analyses Utilizing an Alternative Surrogate Marker for Insulin Resistance

Supplementary Table S24. Multivariable regression analysis of the association between METS-IR and the risk of incident anemia in the CHARLS longitudinal cohort.

| Categories | Model 1 | | Model 2 | | Model 3 | | Model 4 | |
| --- | --- | --- | --- | --- | --- | --- | --- | --- |
|  | OR (95%CI) | *P* value | OR (95%CI) | *P* value | OR (95%CI) | *P* value | OR (95%CI) | *P* value |
| METS-IR | 0.97 (0.96-0.98) | <0.001 | 0.97 (0.96-0.98) | <0.001 | 0.98 (0.97-0.99) | 0.001 | 0.98 (0.97-0.99) | 0.001 |
| METS-IR Quartile |  |  |  |  |  |  |  |  |
| Q1 | 1(Ref) |  | 1(Ref) |  | 1(Ref) |  | 1(Ref) |  |
| Q2 | 0.55 (0.46-0.67) | <0.001 | 0.56 (0.46-0.68) | <0.001 | 0.57 (0.47-0.69) | <0.001 | 0.56 (0.46-0.68) | <0.001 |
| Q3 | 0.51 (0.42-0.62) | <0.001 | 0.53 (0.43-0.64) | <0.001 | 0.56 (0.45-0.68) | <0.001 | 0.55 (0.45-0.67) | <0.001 |
| Q4 | 0.35 (0.29-0.43) | <0.001 | 0.36 (0.29-0.45) | <0.001 | 0.40 (0.32-0.50) | <0.001 | 0.39 (0.31-0.49) | <0.001 |
| *P* for trend |  | <0.001 |  | <0.001 |  | <0.001 |  | <0.001 |

METS-IR: metabolic score for insulin resistance, BMI: body mass index, DM: diabetes mellitus, CPD: chronic pulmonary disease, CKD: chronic kidney disease, MCV: mean corpuscular volume, WBC: white blood cell count, PLT: platelet count, hs-CRP: high-sensitivity C-reactive protein, HbA1c: glycated hemoglobin, BUN: blood urea nitrogen, OR: odds ratio, CI: confidence interval.

Model 1: Unadjusted

Model 2: adjusted for sex, age, race, marital status, residence, educational status, smoking status, drinking status

Model 3: adjusted for Model 2, additionally adjusted for Hypertension, DM, Cancer, CPD, Heart disease, Stroke, Depression, Arthritis, Dyslipidemia, Liver Diseases, CKD, chronic gastritis, Asthma

Model 4: adjusted for Model 3, additionally adjusted for MCV, WBC, PLT, hs-CRP, HbA1c, BUN, and Creatinine

Supplementary Table S25. Interaction analysis of METS-IR and systemic inflammation (hs-CRP) on the risk of incident anemia.

| CHARLS | |
| --- | --- |
|  | hs-CRP |
| Multiplicative interaction |  |
| Multiplicative scale | 1.16(0.69~1.94) |
| *P* | 0.57 |
|  |  |
| Additive interaction |  |
| RERI (95% CI) | 0.10 (-0.35-0.55) |
| AP (95% CI) | 0.14 (-0.45-0.72) |
| SI (95% CI) | NA |

This table evaluates the potential effect modification of systemic inflammation (hs-CRP) on the association between METS-IR and incident anemia. In contrast to the TyG index, both multiplicative interaction (*P*=0.57) and additive interaction metrics (RERI and AP with 95% CIs crossing zero) were not statistically significant for METS-IR. No statistically supported interaction between METS-IR and hs-CRP was observed on either the multiplicative or additive scale. Thus, the inflammation-related interaction pattern observed for the TyG index was not reproduced when METS-IR was used as the alternative insulin-resistance surrogate.

METS-IR, metabolic score for insulin resistance; hs-CRP, high-sensitivity C-reactive protein; RERI, relative excess risk due to interaction; AP, attributable proportion due to interaction; CI, confidence interval; CHARLS, China Health and Retirement Longitudinal Study.

Supplementary Table S26. Exploratory mediation analysis of cystatin C in the association between METS-IR and incident anemia in the CHARLS longitudinal cohort.

| Candidate intermediate marker | Total association | Direct association | Indirect association | 95% CI for indirect association | Proportion statistically accounted for | Statistical support |
| --- | --- | --- | --- | --- | --- | --- |
| Cystatin C | -0.0037 | -0.0037 | 0 | (-0.1198, 0.0516) | 0.26% | *Null* |

This exploratory pathway analysis evaluated whether cystatin C showed an indirect association in the relationship between METS-IR and incident anemia. The estimated indirect association was 0.0000 (95% CI, −0.1198 to 0.0516), and no statistically supported cystatin C-related indirect association was observed. The descriptively estimated proportion statistically accounted for was 0.26%. This contrasts with the cystatin C-related indirect association observed for the TyG index. Because METS-IR and cystatin C were measured concurrently at baseline, these findings should be interpreted as an exploratory statistical decomposition and do not establish temporal or causal mediation or mechanistic specificity.

METS-IR, metabolic score for insulin resistance; TE, Total Effect; DE, Direct Effect; IE, Indirect Effect; CI, confidence interval.

Sensitivity Analysis for Unmeasured Confounding (E-value Analysis)

Supplementary Table S27. E-values for the association between the TyG index and the risk of anemia across all six independent cohorts.

| Cohort | Study Design | Comparison | Adjusted OR (95% CI) | E-value (Point Estimate) |
| --- | --- | --- | --- | --- |
| CHARLS | Longitudinal | Q4 vs Q1 | 0.77 (0.62-0.96) | 1.54 |
| ELSA | Longitudinal | Q4 vs Q1 | 0.58 (0.37-0.89) | 2.84 |
| CHARLS | Cross-sectional | Q4 vs Q1 | 0.63 (0.53-0.75) | 1.83 |
| NHANES | Cross-sectional | Q4 vs Q1 | 0.44 (0.34-0.58) | 3.97 |
| Ganzhou | Cross-sectional | Q4 vs Q1 | 0.39 (0.34-0.46) | 4.57 |
| Zhaotong | Cross-sectional | Q4 vs Q1 | 0.46 (0.34-0.62) | 3.77 |

To quantitatively assess the robustness of our primary findings against potential unmeasured or residual confounding, we calculated the E-value for the point estimate of the fully adjusted odds ratio (OR) comparing the highest quartile (Q4) to the lowest quartile (Q1) of the TyG index. The E-value indicates the minimum strength of association, on the risk ratio scale, that an unmeasured confounder would need to have with both the exposure (TyG index) and the outcome (anemia)—above and beyond the measured covariates—to completely explain away the observed inverse association. Across the six cohorts, the computed E-values ranged from 1.54 to 4.57. For instance, in the Ganzhou cohort, an unmeasured confounder would need to be associated with both the TyG index and anemia by a magnitude of at least 4.57-fold to negate the observed association. E-values were calculated for the point estimates of the fully adjusted associations comparing the highest with the lowest TyG quartile. For inverse associations, reciprocal effect estimates were used for calculation. Larger E-values indicate that stronger associations of an unmeasured confounder with both TyG and anemia would be required to fully explain the observed association, conditional on the measured covariates. E-values quantify sensitivity to unmeasured confounding but do not address other sources of bias or establish causality.

TyG, triglyceride-glucose index; OR, odds ratio; CI, confidence interval; CHARLS, China Health and Retirement Longitudinal Study; ELSA, English Longitudinal Study of Ageing; NHANES, National Health and Nutrition Examination Survey.

Exploratory Cystatin C Pathway Analysis by Baseline CKD Status

Supplementary Table S28. Exploratory mediation analysis of cystatin C in the association between the TyG index and incident anemia, stratified by baseline CKD status.

| Baseline renal context | Total association | Direct association | Indirect association | 95% CI for indirect association | Proportion statistically accounted for | Statistical support |
| --- | --- | --- | --- | --- | --- | --- |
| 1. Cystatin C | -0.033 | -0.028 | -0.0053 | (-0.0098, -0.0011) | 16.33% | Indirect association supported |
| *(Renal Function)* |  |  |  | *(Does not cross 0)* |  |  |
| 2. Cystatin C | -0.035 | -0.0298 | -0.0052 | (-0.0094, -0.0016) | 15.12% | Indirect association supported |
| *(No CKD)* |  |  |  | *(Does not cross 0)* |  |  |
| 3. Cystatin C | -0.0013 | 0.0068 | -0.0082 | (-0.0262, 0.0004) | Not interpreted | *No statistical evidence of an indirect association* |
| *(CKD)* |  |  |  | *(Crosses 0)* |  |  |

Indirect associations were estimated using bootstrap mediation analysis with 1,000 resamples. Statistical support was evaluated using the bootstrap 95% CI for the indirect effect; an interval excluding zero was considered supportive of an indirect association. Because TyG and cystatin C were measured concurrently at baseline, these estimates should be interpreted as exploratory and do not establish temporal or causal mediation. The proportion mediated was not interpreted in the CKD subgroup because the total effect was close to null.

CHARLS, China Health and Retirement Longitudinal Study; TyG, triglyceride-glucose; CKD, chronic kidney disease; TE, Total Effect; DE, Direct Effect; IE, Indirect Effect; CI, confidence interval

Sensitivity Analyses with Additional Lifestyle, Nutritional, Frailty, Psychological, and Medication Covariates

Supplementary Table S29. Sensitivity analyses additionally accounting for sleep and physical activity in the CHARLS longitudinal cohort

| Exposure | Primary analysis*, OR (95% CI) | + Sleep | + Physical activity | + Sleep + Physical activity |
| --- | --- | --- | --- | --- |
| TyG, continuous | 0.87 (0.77-0.98) | 0.88 (0.78~1.00) | 0.86 (0.76~0.98) | 0.88 (0.77~0.99) |
| *P* value | 0.025 | 0.045 | 0.019 | 0.041 |
| TyG, categorical |  |  |  |  |
| Q1 | Reference | Reference | Reference | Reference |
| Q2 | 0.92 (0.76-1.11) | 0.92 (0.76~1.11) | 0.91 (0.75~1.10) | 0.91 (0.75~1.10) |
| Q3 | 0.83 (0.68-1.01) | 0.84 (0.68~1.02) | 0.81 (0.67~0.99) | 0.82 (0.67~1.00) |
| Q4 | 0.77 (0.62-0.96) | 0.79 (0.64~0.99) | 0.77 (0.62~0.96) | 0.78 (0.63~0.98) |
| *P* for trend | 0.014 | 0.024 | 0.010 | 0.017 |

*The primary analysis estimate is shown for reference and was obtained from the original analytic dataset; the three sensitivity models were estimated after multiple imputation.

Values are presented as ORs (95% CIs). Missing covariate data were handled using multiple imputation. All sensitivity models included the covariates from the original fully adjusted model. The + Sleep model additionally included sleep duration and sleep quality; the + Physical activity model additionally included physical activity; and the + Sleep + Physical activity model additionally included sleep duration, sleep quality, and physical activity. *P* for trend was calculated by modeling TyG quartiles as an ordinal variable.

Original fully adjusted model: adjusted for sex, age, race, marital status, residence, educational status, smoking status, drinking status, BMI, Hypertension, DM, Cancer, CPD, Heart Diseases, Stroke, Depression, Arthritis, Dyslipidemia, Liver Diseases, CKD Chronic. gastritis, Asthma,MCV, WBC, PLT, hs-CRP, HbA1c, BUN, and Creatinine

CHARLS, China Health and Retirement Longitudinal Study, TyG, triglyceride-glucose, BMI: body mass index, WBC: white blood cell count, PLT: platelet count, MCV: mean corpuscular volume, hs-CRP: high-sensitivity C-reactive protein, HbA1c: glycated hemoglobin, BUN: blood urea nitrogen, DM: diabetes mellitus, CPD: chronic pulmonary disease, CKD: chronic kidney disease, OR: odds ratio, CI: confidence interval.

Supplementary Table S30. Sensitivity analyses additionally accounting for sleep and physical activity in the ELSA cohort

| Exposure | Primary analysis*, OR (95% CI) | + Sleep | + Physical activity | + Sleep + Physical activity |
| --- | --- | --- | --- | --- |
| TyG, continuous | 0.62 (0.45-0.86) | 0.64 (0.47~0.88) | 0.61 (0.45~0.85) | 0.62 (0.45~0.85) |
| *P* value | 0.005 | 0.006 | 0.003 | 0.003 |
| TyG, categorical |  |  |  |  |
| Q1 | Reference | Reference | Reference | Reference |
| Q2 | 0.86 (0.59-1.26) | 0.87 (0.59~1.27) | 0.86 (0.59~1.26) | 0.86 (0.59~1.26) |
| Q3 | 0.58 (0.38-0.88) | 0.59 (0.39~0.90) | 0.56 (0.37~0.86) | 0.57 (0.37~0.86) |
| Q4 | 0.58 (0.37-0.89) | 0.60 (0.39~0.91) | 0.57 (0.37~0.87) | 0.57 (0.37~0.87) |
| *P* for trend | 0.004 | 0.005 | 0.002 | 0.002 |

*The primary analysis estimate is shown for reference and was obtained from the original analytic dataset; the three sensitivity models were estimated after multiple imputation.

Values are presented as ORs (95% CIs). Missing covariate data were handled using multiple imputation. All sensitivity models included the covariates from the original fully adjusted model. The + Sleep model additionally included sleep duration and sleep quality; the + Physical activity model additionally included physical activity; and the + Sleep + Physical activity model additionally included sleep duration, sleep quality, and physical activity. *P* for trend was calculated by modeling TyG quartiles as an ordinal variable.

Original fully adjusted model: adjusted for age, sex, race, marital status, educational status, smoking status, drinking status, BMI, Hypertension, DM, Cancer, CPD, Heart Diseases, Stroke, Arthritis, WBC, hs-CRP, HbA1c, antihypertensives, antidiabetics, respiratory drugs, antithrombotic drugs, and lipid-lowering drugs.

ELSA, English Longitudinal Study of Ageing; TyG, triglyceride-glucose; BMI: body mass index, WBC: white blood cell count, hs-CRP: high-sensitivity C-reactive protein, HbA1c: glycated hemoglobin, DM: diabetes mellitus, CPD: chronic pulmonary disease, CKD: chronic kidney disease, OR: odds ratio, CI: confidence interval.

Supplementary Table S31. Sensitivity analyses additionally accounting for sleep and physical activity in the NHANES cohort

| Exposure | Primary analysis*, OR (95% CI) | + Sleep | + Physical activity | + Sleep + Physical activity |
| --- | --- | --- | --- | --- |
| TyG, continuous | 0.61 (0.52~0.71) | 0.60 (0.51~0.70) | 0.61 (0.52~0.71) | 0.59 (0.51~0.70) |
| *P* value | <0.001 | <0.001 | <0.001 | <0.001 |
| TyG, categorical |  |  |  |  |
| Q1 | Reference | Reference | Reference | Reference |
| Q2 | 0.80 (0.63~1.00) | 0.79 (0.63~1.00) | 0.80 (0.63~1.01) | 0.79 (0.63~1.00) |
| Q3 | 0.64 (0.49~0.82) | 0.63 (0.49~0.82) | 0.63 (0.49~0.82) | 0.62 (0.48~0.81) |
| Q4 | 0.44 (0.34~0.58) | 0.44 (0.33~0.58) | 0.44 (0.34~0.59) | 0.43 (0.33~0.57) |
| *P* for trend | <0.001 | <0.001 | <0.001 | <0.001 |

*The primary analysis estimate is shown for reference and was obtained from the original analytic dataset; the three sensitivity models were estimated after multiple imputation.

Values are presented as ORs (95% CIs). Missing covariate data were handled using multiple imputation. All sensitivity models included the covariates from the original fully adjusted model. The + Sleep model additionally included sleep duration and sleep quality; the + Physical activity model additionally included physical activity; and the + Sleep + Physical activity model additionally included sleep duration, sleep quality, and physical activity. *P* for trend was calculated by modeling TyG quartiles as an ordinal variable.

Original fully adjusted model: adjusted for age, sex, race/ethnicity, marital status, educational status, smoking status, drinking status, BMI, PIR, hypertension, dyslipidemia, diabetes mellitus, CKD, COPD, cancer, depression, asthma, arthritis, heart disease, creatinine, BUN, MCV, vitamin B12, iron, total folate, WBC, RDW, and PLT.

CHARLS, China Health and Retirement Longitudinal Study, TyG, triglyceride-glucose, BMI: body mass index, WBC: white blood cell count, PLT: platelet count, MCV: mean corpuscular volume, hs-CRP: high-sensitivity C-reactive protein, HbA1c: glycated hemoglobin, BUN: blood urea nitrogen, DM: diabetes mellitus, CPD: chronic pulmonary disease, CKD: chronic kidney disease, OR: odds ratio, CI: confidence interval.

All NHANES analyses accounted for survey weights, strata, and primary sampling units.

Supplementary Table S32. Sensitivity analysis of the association between the TyG index and anemia after additional adjustment for the continuous frailty index.

| Variables | CHARLS cross | CHARLS long | NHANES | ELSA |
| --- | --- | --- | --- | --- |
| Total *N*/  Events *N* (%) | 10732/1723 (16.10) | 5927/1048 (17.70) | 14559/1716 (8.01*) | 2459/226 (9.20) |
| Model 4  (Fully adjusted + FI) | 0.77 (0.7~0.85) | 0.87 (0.77~0.99) | 0.54 (0.46~0.64) | 0.61 (0.44~0.84) |
| *P* | <0.001 | 0.031 | <0.001 | 0.003 |
| Model 4  (Fully adjusted + FI) | OR (95%CI) | OR (95%CI) |  | OR (95%CI) |
| Q1(Ref) | 1.00 | 1.00 | 1.00 | 1.00 |
| Q2 | 0.77 (0.67~0.89) | 0.93 (0.77~1.12) | 0.75 (0.58~0.95) | 0.87 (0.59~1.27) |
| Q3 | 0.67 (0.58~0.78) | 0.83 (0.68~1.02) | 0.59 (0.45~0.77) | 0.57 (0.38~0.87) |
| Q4 | 0.63 (0.53~0.75) | 0.79 (0.63~0.98) | 0.36 (0.26~0.49) | 0.56 (0.37~0.87) |
| *P* for trend | <0.001 | 0.020 | <0.001 | 0.002 |

*In the descriptive row 'Total N / Events N (%)', the percentage for the NHANES cohort represents the weighted prevalence of anemia, whereas percentages for other cohorts are unweighted crude rates.

Data are presented as odds ratios (ORs) with 95% confidence intervals (CIs). All analyses were based on the cohort-specific fully adjusted Model 4, with additional adjustment for the frailty index (FI). TyG was examined both as a continuous variable and in cohort-specific quartiles, with the lowest quartile (Q1) serving as the reference group. The FI was modeled as a continuous variable.

NHANES estimates accounted for the complex survey design.

CHARLS, China Health and Retirement Longitudinal Study; ELSA, English Longitudinal Study of Ageing; NHANES, National Health and Nutrition Examination Survey; TyG, triglyceride-glucose index; FI, frailty index; OR, odds ratio; CI, confidence interval.

Supplementary Table S33. Sensitivity analyses additionally accounting for dietary intake, nutritional status, and serum albumin in the NHANES cohort

| Exposure | Primary analysis*, OR (95% CI) | + dietary intake | + albumin | + dietary intake+ albumin |
| --- | --- | --- | --- | --- |
| TyG, continuous | 0.61 (0.52~0.71) | 0.61 (0.53~0.72) | 0.62 (0.53~0.74) | 0.61 (0.51~0.73) |
| *P* value | <0.001 | <0.001 | <0.001 | <0.001 |
| TyG, categorical |  |  |  |  |
| Q1 | Reference | Reference | Reference | Reference |
| Q2 | 0.80 (0.63~1.00) | 0.81 (0.64~1.01) | 0.83 (0.66~1.05) | 0.81 (0.64~1.03) |
| Q3 | 0.64 (0.49~0.82) | 0.64 (0.50~0.84) | 0.67 (0.52~0.87) | 0.66 (0.50~0.85) |
| Q4 | 0.44 (0.34~0.58) | 0.45 (0.34~0.59) | 0.46 (0.34~0.62) | 0.45 (0.33~0.61) |
| *P* for trend | <0.001 | <0.001 | <0.001 | <0.001 |

*The primary analysis estimate is shown for reference and was obtained from the original analytic dataset; the three sensitivity models were estimated after multiple imputation.

Values are presented as ORs (95% CIs). Missing covariate data were handled using multiple imputation. All sensitivity models included the covariates from the original fully adjusted model. The + dietary intake model additionally accounted for available dietary intake information, and the + dietary intake + albumin model further adjusted for serum albumin. *P* for trend was calculated by modeling TyG quartiles as an ordinal variable.

Original fully adjusted model: adjusted for Age, Sex, Race, marital status, educational status, smoking status, drinking status, BMI, PIR, Hypertension, Hyperlipidemia, DM, CKD, COPD, Cancer, Depression, Asthma, Arthritis, heart disease, creatinine, BUN, MCV, VB12, iron, Total folate, WBC, RDW, PLT.

NHANES: National Health and Nutrition Examination Survey, BMI: body mass index, PIR: poverty income ratio, DM: Diabetes Mellitus, CKD: Chronic Kidney Disease, COPD: Chronic Obstructive Pulmonary Disease, CHD: Coronary Heart Disease, BUN: blood urea nitrogen, MCV: Mean Corpuscular Volume, VB12: vitamin B12, WBC: white blood cell, RDW: red blood cell distribution width, PLT: blood platelet, OR: odds ratio, CI: confidence interval.

Supplementary Table S34. Sensitivity analysis of the association between the TyG index and incident anemia after additional adjustment for depressive symptoms in the ELSA cohort

| Categories | Model 1 | | Model 2 | | Model 3 | | Model 4 | |
| --- | --- | --- | --- | --- | --- | --- | --- | --- |
|  | OR (95%CI) | *P* value | OR (95%CI) | *P* value | OR (95%CI) | *P* value | OR (95%CI) | *P* value |
| TyG | 0.84 (0.64-1.12) | 0.234 | 0.75 (0.55-1.02) | 0.065 | 0.73 (0.53-0.99) | 0.044 | 0.66 (0.48~0.90) | 0.009 |
| TyG Quartile |  |  |  |  |  |  |  |  |
| Q1 (<8.23) | 1(Ref) |  | 1(Ref) |  | 1(Ref) |  | 1(Ref) |  |
| Q2 (8.23-8.54) | 0.98 (0.68-1.42) | 0.925 | 0.88 (0.60-1.28) | 0.503 | 0.87 (0.59-1.27) | 0.464 | 0.87 (0.59~1.28) | 0.476 |
| Q3 (8.54-8.89) | 0.69 (0.47-1.03) | 0.072 | 0.58 (0.39-0.88) | 0.010 | 0.59 (0.39-0.90) | 0.014 | 0.61 (0.41~0.93) | 0.022 |
| Q4 (>8.89) | 0.80 (0.54-1.17) | 0.246 | 0.68 (0.45-1.02) | 0.065 | 0.66 (0.43-1.00) | 0.049 | 0.61 (0.40~0.93) | 0.023 |
| *P* for trend |  | 0.100 |  | 0.019 |  | 0.016 |  | 0.008 |

ELSA, English Longitudinal Study of Ageing; TyG, triglyceride-glucose; BMI: body mass index, WBC: white blood cell count, hs-CRP: high-sensitivity C-reactive protein, HbA1c: glycated hemoglobin, DM: diabetes mellitus, CPD: chronic pulmonary disease, CKD: chronic kidney disease, OR: odds ratio, CI: confidence interval.

Model 1: Unadjusted

Model 2: adjusted for age, sex, race, marital status, educational status, smoking status, drinking status, and BMI

Model 3: adjusted for Model 2 and additionally adjusted for Hypertension, DM, Cancer, CPD, Heart Diseases, Stroke, Arthritis

Model 4: adjusted for Model 3 and additionally adjusted for WBC, hs-CRP, HbA1c, antihypertensives, antidiabetics, respiratory drugs, antithrombotic drugs, and lipid-lowering drugs, CES-D score

Supplementary Table S35. Sensitivity analysis of the association between the TyG index and anemia after additional adjustment for cardiometabolic medication use in the CHARLS cohorts.

| Variables | CHARLS cross | CHARLS long |
| --- | --- | --- |
| Total *N*/  Events *N* (%) | 10732/1723 (16.10) | 5927/1048 (17.70) |
| Model 4  (Fully adjusted + cardiometabolic medications) | 0.77 (0.70~0.85) | 0.86 (0.76~0.98) |
| *P* | <0.001 | 0.020 |
| Model 4  (Fully adjusted + cardiometabolic medications) | OR (95%CI) | OR (95%CI) |
| Q1(Ref) | 1.00 | 1.00 |
| Q2 | 0.77 (0.67~0.89) | 0.92 (0.77~1.11) |
| Q3 | 0.68 (0.58~0.79) | 0.82 (0.68~1.00) |
| Q4 | 0.64 (0.53~0.76) | 0.77 (0.62~0.96) |
| *P* for trend | <0.001 | 0.011 |

Data are presented as odds ratios (ORs) with 95% confidence intervals (CIs). The fully adjusted models included age, sex, race, marital status, residence, educational status, smoking status, drinking status, BMI, hypertension, DM, cancer, CPD, heart disease, stroke, depressive symptoms, arthritis, dyslipidemia, liver disease, CKD, chronic gastritis, asthma, MCV, WBC, PLT, hs-CRP, HbA1c, BUN, and creatinine, with additional adjustment for the use of antihypertensive, glucose-lowering, and lipid-lowering medications. TyG was modeled both continuously and in quartiles, with the lowest quartile (Q1) serving as the reference group.

CHARLS, China Health and Retirement Longitudinal Study; TyG, triglyceride-glucose index; OR, odds ratio; CI, confidence interval; BMI, body mass index; DM, diabetes mellitus; CPD, chronic pulmonary disease; CKD, chronic kidney disease; MCV, mean corpuscular volume; WBC, white blood cell count; PLT, platelet count; hs-CRP, high-sensitivity C-reactive protein; HbA1c, glycated hemoglobin; BUN, blood urea nitrogen.
